# Phase Change Materials in Residential Buildings: Challenges, Opportunities, and Performance

**DOI:** 10.3390/ma18092063

**Published:** 2025-04-30

**Authors:** José Pereira, Reinaldo Souza, Jeferson Oliveira, Ana Moita

**Affiliations:** 1 IN+ Center for Innovation, Technology and Policy Research, Instituto Superior Técnico, Universidade de Lisboa, Avenida Rovisco Pais, 1049-001 Lisboa, Portugal; reinaldo.souza@tecnico.ulisboa.pt (R.S.); anamoita@tecnico.ulisboa.pt (A.M.); 2 Metrics, Mechanical Engineering Department, University of Minho, Campus de Azurém, 4800-058 Guimarães, Portugal; 3 SISEA—Renewable and Alternative Energy Systems Laboratory, Polytechnic School of the University of São Paulo, São Paulo 05508-010, Brazil; jef.diehl@usp.br; 4 CINAMIL, Centro de Investigação Desenvolvimento e Inovação da Academia Militar, Academia Militar, Instituto Universitário Militar, Rua Gomes Freire, 1169-203 Lisboa, Portugal

**Keywords:** PCMs, thermal management, residential buildings

## Abstract

Phase change materials (PCMs) have emerged as promising solutions for improving thermal management in residential buildings by enhancing thermal storage capacity and reducing energy consumption. This article aims to provide a comprehensive analysis of the application of PCMs in residential construction, focusing on their thermal properties, benefits, and limitations. A systematic literature review was conducted following PRISMA guidelines, primarily covering studies published between 2015 and 2025. However, key studies published outside this period were also considered due to their relevance and significant contribution to the understanding of PCM performance and application. This analysis explores key parameters affecting PCM performance, including phase transition temperature, thermal conductivity, and material stability. The results highlight that optimized PCM integration can reduce energy consumption by up to 30% and improve indoor thermal comfort. However, challenges such as low thermal conductivity and phase separation still limit their large-scale adoption. The findings provide insights into the advantages and barriers associated with PCM-based systems and propose strategies to enhance their performance, including the use of nanocomposites and improved encapsulation techniques.

## 1. Introduction

In recent decades, there has been a significant rise in sustainable and clean energy technologies due to ever-increasing global energy demands and environmental challenges. Additionally, environmental agreements highlight the urgent need to provide green and affordable energy worldwide, emphasizing the adoption of renewable and energy-efficient resources. Buildings are major energy consumers, accounting for up to 45% of global energy consumption [1]. Therefore, enhancing building energy efficiency is essential to reduce global energy use, promote environmental sustainability, and improve the availability of energy supplies. The change in living standards and the increasing demand for heating and cooling in cold and hot climates are the main causes of the rising energy consumption. Thermal energy remains an abundant energy source, which is widely present in nature and available as a by-product of various energy conversion processes.

The concept of thermal energy storage (TES) presents a promising technique to reuse abundant thermal energy and improve energy efficiency. Thermal energy storage systems should be developed and implemented to collect, store, and reuse energy in the form of heat whenever needed. Furthermore, to adequately assess the thermodynamic behavior of buildings and systems under dynamic working conditions, it is essential to understand the thermal response characteristics of buildings and the interactions at the system and subsystem levels. This can be achieved through numerical simulations and/or experimental investigations. With the aid of these simulations, advanced control strategies can be developed for effective thermal load management, optimizing the benefits of using nano-enhanced materials for thermal control in buildings. The selection of control strategies should consider the utility rate structure, the thermophysical properties of the materials used, local climatic conditions, and the load profile of the buildings.

In this context, PCMs emerge as a technological solution capable of maximizing the utilization of thermal energy in buildings, whilst reducing energy consumption [2,3]. These materials can store or release large amounts of heat during phase transition, mitigating the need for traditional heating or cooling systems.

**Figure 1:**
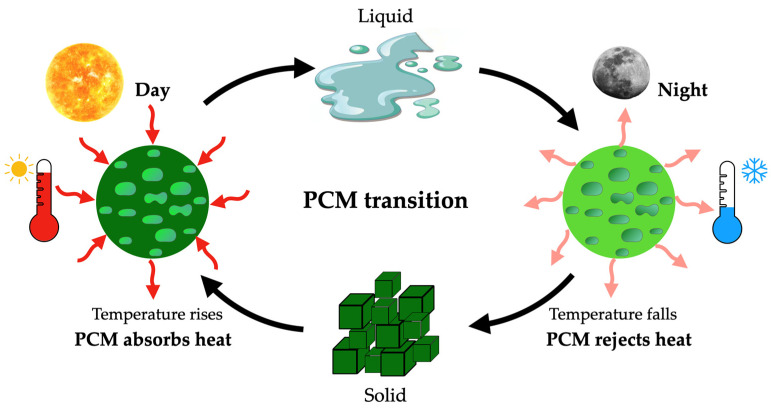
The phase transition of PCMs considering ambient conditions during the day and night.

The versatility of PCMs’ usage is another important aspect to consider. They can be incorporated into various structural elements of a residence, such as walls, ceilings, and floors, among others [4,5]. Depending on ambient temperature variations, they can regulate indoor thermal comfort by absorbing or releasing heat. Also, the design and implementation of PCM-based solutions should always consider PCM transition considering the different ambient conditions occurring during the day and night and act accordingly to obtain the most moderate indoor ambience, as is illustrated in Figure 1.

Despite the benefits of PCMs, significant challenges still need to be overcome, such as thermal stability in time, encapsulation capability, compatibility with traditional construction materials, and overall production costs [6]. There is also a growing interest from the research community in designing and implementing certain composite solutions using PCMs. In this sense, recent studies have explored the incorporation of nanoparticles or other particulates to enhance the thermal conductivity of PCMs [7,8,9]. Furthermore, with advances in numerical modeling and experimental techniques, the potential to improve the understanding of the thermal behavior of these materials in residential buildings is very promising [10,11].

This study aims to explore the application of PCM-assisted solutions in the thermal management of residential buildings, with a focus on recent advancements, existing challenges, and strategies to optimize their thermal performance. The mechanisms of energy storage and release within PCMs, as well as thermal enhancement techniques and future trends for their integration into energy-efficient buildings, will be discussed. Additionally, this work aims to compare the performance of PCMs in different building configurations, highlighting the improvements in thermal comfort and energy efficiency over conventional solutions.

## 2. Methodology

A systematic literature review was conducted following the Preferred Reporting Items for Systematic Reviews and Meta-Analyses (PRISMA) guidelines to evaluate the thermal performance of PCMs in residential buildings and identify the associated opportunities and challenges.

### 2.1. Data Sources and Search Strategy

The literature search was performed using the databases Scopus and Web of Science. These databases were selected due to their extensive coverage of peer-reviewed journals in materials science, building technology, and energy efficiency, ensuring access to relevant studies. The search focused primarily on studies published between 2015 and 2025 to capture recent advancements in the PCM technology field, given the rapid development in this field over the past decade. Nonetheless, studies published prior to 2015 were also included if they provided valuable insights into PCM science and behavior.

The keywords were selected based on the research objectives that included understanding PCM properties, thermal performance, and challenges in residential buildings. An iterative process was employed to refine the keywords, starting with broad terms like “phase change materials” and “residential buildings” and, after that, adding specific terms like “thermal energy storage”, “encapsulation”, and “thermal conductivity” based on preliminary search results. The Boolean search strings were built as follows:

- Scopus: (“phase change material” OR “PCM”) AND (“residential building” OR “housing” OR “domestic building”) AND (“thermal performance” OR “energy efficiency” OR “thermal storage” OR “heat transfer” OR “encapsulation”).
- Web of Science: (“phase change material” OR PCM) AND (“residential building” OR housing OR “domestic building”) AND (“thermal performance” OR “energy efficiency” OR “thermal conductivity” OR “latent heat”).

### 2.2. Inclusion/Exclusion Criteria

The studies were included in the review if they addressed the following:

- PCM integration into residential buildings in walls, ceilings, floors, and windows, among other elements.
- Thermal performance metrics exposing heat flux, thermal conductivity, latent heat, and indoor thermal comfort.
- Quantitative/qualitative data comparing PCM-integrated systems with traditional building materials.
- Publication in peer-reviewed journals or conference proceedings.
- The papers were excluded if they met the following criteria:
- They focused exclusively on industrial, commercial, or non-building applications of PCMs.
- They did not provide quantitative/qualitative data on PCM performance.
- They were not peer-reviewed, technical reports, or non-academic publications.

### 2.3. Study Selection Process

**Figure 2:**
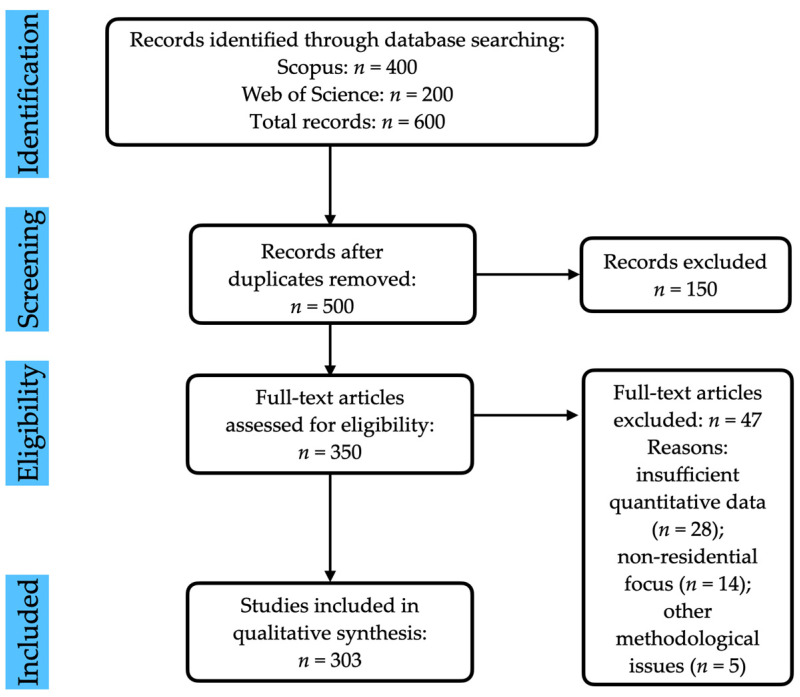
PRISMA workflow diagram for literature search and selection.

The study selection process followed the PRISMA framework, as illustrated in Figure 2. The initial search across Scopus and Web of Science yielded 600 records (400 from Scopus and 200 from Web of Science). After removing duplicates (*n* = 100), 500 unique records were screened based on titles and abstracts. Of these, 150 were excluded for not meeting the inclusion criteria (e.g., irrelevant applications, lack of focus on residential buildings, or non-peer-reviewed status). The remaining 350 full-text articles were assessed for eligibility, with 47 excluded due to insufficient quantitative data (*n* = 28), non-residential focus (*n* = 14), or other methodological issues (*n* = 5). Ultimately, 303 studies were included in the qualitative synthesis, covering PCM types, properties, integration methods, and performance outcomes.

### 2.4. Data Extraction and Analysis

From the selected scientific papers, data were extracted based on the following factors:

- PCM properties (e.g., phase transition temperature, latent heat, thermal conductivity).
- Types of building elements with PCM integration (walls, ceilings, floors, windows, among others).
- Thermal performance outcomes, including energy savings, temperature regulation, and indoor comfort improvement.
- Technical challenges, such as phase separation, low thermal conductivity, leakage, and overall investment cost.

The results are summarized in Table 1, grouping studies by PCM type and application method, facilitating a critical discussion of their effectiveness and limitations.

## 3. Properties and Types of PCMs

**Figure 3:**
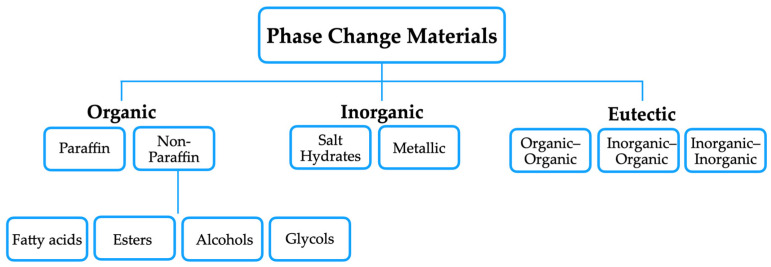
Main types of PCMs.

PCMs can be classified into three main categories: organic (such as paraffin and non-paraffin), inorganic (such as metallic salts and salt hydrates), and eutectic mixtures (organic–organic, inorganic–inorganic, and inorganic–organic). An ideal PCM should exhibit a high latent heat of fusion, anti-corrosive properties, enhanced thermal stability, a melting temperature between 20 °C and 100 °C, and cost effectiveness. These properties are essential to ensure that the material can absorb and release large amounts of energy during phase transition, while maintaining its thermal stability [6,38]. Figure 3 summarizes the main types of PCMs.

The most noticeable attribute of PCMs is that they have the ability to take in and emit huge amounts of latent heat while undergoing phase change. The latent heat of fusion, or the amount of energy required to change a material from solid to liquid, is a key parameter to determine PCM performance. For example, paraffin wax, a typical organic PCM, has a latent heat of fusion between 150 and 250 kJ/kg [39]. It is this high potential for energy storage that makes PCMs appropriate for application in areas where proper thermal management is required.

Latent Heat Energy Storage (LHES) systems and their derivatives have already demonstrated an enhanced energy storage density capability. Additionally, these systems have a well-defined operating temperature range, making PCMs a suitable choice for LHES systems.

In the case of organic PCMs, such as paraffins, which possess high latent heat, these stand out as excellent practical agents for thermal energy storage. However, one of the main challenges associated with their use is their poor intrinsic thermal conductivity. This characteristic results in relatively high interfacial thermal resistance between the base PCM and the dispersed materials, such as nanoparticles and nanofillers, which may compromise the charging efficiency of the material during the thermal energy storage process.

The phase change temperature of a PCM is another significant property. The melting point of a PCM must be compatible with the operating temperature range of the application in which it is to be employed. For instance, for application in building energy efficiency, PCMs with melting temperatures between 18 °C and 30 °C are suitable because they can be utilized to maintain indoor temperatures within the comfort zone by absorbing excess heat during the day and releasing it at night [40].

On the other hand, inorganic PCMs, such as salt hydrates, offer superior thermal conductivity but face challenges like supercooling [6], which may limit their application under certain conditions. To address this issue, researchers have explored various methods to enhance thermal conductivity, such as incorporating metal foams, nanoparticles, or graphite into the PCM matrix [41]. These enhancements can significantly improve the efficiency of PCM-based systems.

**Figure 4:**
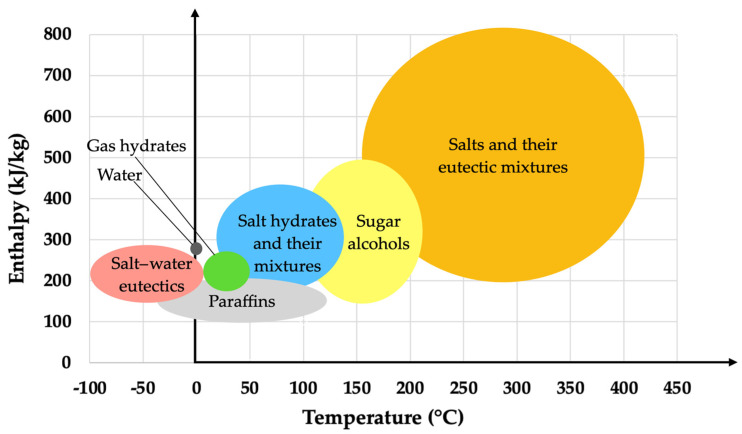
Plot of working temperature vs. enthalpy of main existing PCMs.

Eutectic PCMs, formed by combining two or more compounds, can have their thermophysical properties enhanced. This is because a eutectic mixture can be designed to optimize the melting point and improve the thermal conductivity and stability. By combining materials with complementary characteristics, it is possible to obtain a PCM with improved heat transfer performance and thermal stability, even after multiple fusion and solidification cycles [42]. These materials offer advantages over pure PCMs, as the phase transition occurs at a more stable temperature with less variation, resulting in a more efficient and long-lasting performance. However, the main drawback of these compounds is their cost, which can be two to three times higher compared to organic or inorganic PCMs [38]. Figure 4 shows the working temperature against enthalpy of the main existing PCMs.

In a study conducted by Min et al. [12], a new composite PCM (FCPCM) was developed using binary paraffin (BP), obtained by fusing solid paraffin (SP) and liquid paraffin (LP), with polypropylene (PP) and waste cellular concrete (WCC) as support materials. The FCPCM was synthesized through fusion and vacuum adsorption to meet the energy storage demands of buildings. In addition to the good chemical compatibility and morphological stability verified through thermal and structural analyses, the encapsulated material exhibited a 14.08% increase in melting temperature and a 42.94% reduction in the latent heat of phase change while maintaining its thermal properties unchanged even after multiple cycles. The authors also highlighted its thermal stability up to 150 °C and a leakage rate below 1% after 24 h, which was 44% lower compared to FCPCM using only PP as a support material. Thermogravimetric analysis (TGA) revealed a 25.59% increase in thermal stability. The thermal performance tests on energy storage concrete demonstrated a higher thermal storage density and improved temperature control capability compared to conventional concrete, contributing to greater energy efficiency in buildings.

PCMs must retain steady performance after numerous cycles of solidification and melting in order to be applied to long-term usage. With time, some PCMs might decay in terms of thermal performance or experience phase separation, consequently decreasing their effectiveness. Encapsulation procedures, whereby PCM is shielded with an outer coating, have been proven to improve cycling stability and leak resistance [43].

In general, those materials typically undergo volume change during phase change, expanding on melting and contracting on solidification. Water/ice PCMs, for example, expand by approximately 9% on freezing [44]. This feature needs to be handled with care in system design to avoid structural damage or inefficiencies.

For construction and use, PCMs must be chemically stable, non-toxic, and non-flammable. Organic PCMs such as paraffins and fatty acids are generally stable and safe for use in buildings and electronics. In contrast, inorganic PCMs such as salt hydrates may require special care due to the risk of corrosion or subcooling [45].

**Figure 5:**
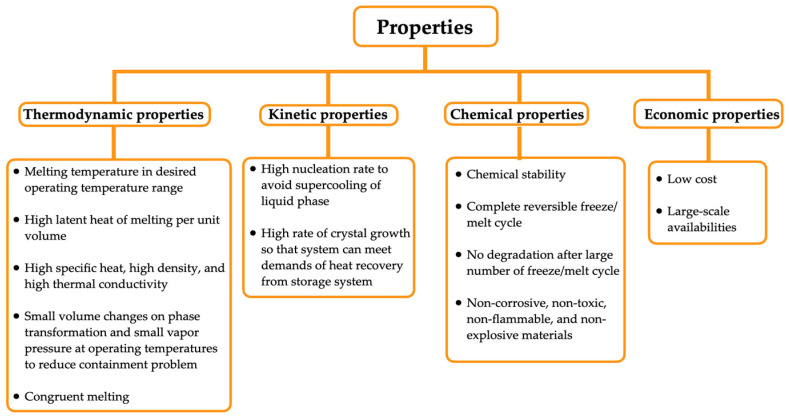
Classification criteria and key properties for PCM selection.

In addition to the classification of PCMs being related to the material properties and their changes with temperature, other parameters such as thermodynamic, kinetic, and chemical properties, as well as economic aspects, are also considered. A list of these parameters is presented in Figure 5 below.

Within this set of properties, shown in Figure 5, for choosing the type of PCM, thermodynamic properties stand out the most. For example, organic PCMs, such as esters, exhibit a wide range of melting temperatures and minimal superheating, which increases their thermal stability [46]. On the other hand, nitrate-based PCMs have demonstrated promising thermal properties, including high heat storage capacity and stability at high temperatures [47].

Another important aspect is kinetic properties, which are related to the melting and crystallization processes of PCMs. These processes have been optimized through the incorporation of nanoparticles, as demonstrated in the work [9], in addition to increasing the thermal conductivity of these composite materials. Understanding the kinetic behavior of PCMs is crucial for their application in thermal energy systems, influencing the design and efficiency of heat exchangers, as highlighted in [48].

Regarding chemical properties, organic PCMs are often biodegradable and exhibit low corrosivity, making them environmentally friendly alternatives [46]. The chemical stability of PCMs, such as those derived from the lost-wax casting industry, has been shown to be comparable to or even superior to commercial options, as demonstrated in [49].

The economic aspect has shown that the cost–benefit ratio in the use of PCMs is a significant factor. Materials such as NaOH and CaCl_2_·6H_2_O offer low storage costs, making them attractive options for applications requiring energy storage, as highlighted in [48]. Additionally, using industrial by-products as PCMs can reduce costs and promote sustainability, providing not only economic benefits but also environmental advantages, as mentioned in [49].

## 4. PCM Incorporation Methods

PCMs can be incorporated into building envelope elements through various methods. These methods aim to enhance the thermal performance of a structure by improving its ability to regulate temperature. The following section explores the key techniques used to incorporate PCMs:

(a) Direct incorporation.
(b) Immersion.
(c) Encapsulation (macroencapsulation, microencapsulation, nanoencapsulation).
(d) Shape-stabilized PCM.
(e) Form-stabilized PCM.

### 4.1. Direct Incorporation

In the direct incorporation technique, PCMs, whether in powder or liquid form, are blended directly into construction materials such as gypsum mortar, cement mortar, or concrete mixes. This approach is considered both straightforward and cost-effective, as it does not demand specialized skills and allows for simple integration into the building matrix [50]. However, a significant drawback is the risk of PCM leakage during its phase transition, particularly during melting. This can lead to material incompatibilities and, in the case of flammable PCMs, elevate fire hazards. Additionally, when PCMs are introduced in liquid form, they tend to reduce the water content of the mixture, which may impair the mechanical strength of the final construction elements, especially under high temperatures [51].

Feldman et al. [52], for instance, applied this method to embed organic PCMs (referred to as BS) into gypsum boards at concentrations of 21% and 22%. To improve the incorporation process, they used supporting materials that facilitated the integration. The results demonstrated that the PCM-enhanced gypsum stored thermal energy at levels up to ten times greater than those of standard gypsum wallboards.

Cunha et al. [53] emphasized that one of the advantages of direct incorporation is that it eliminates the need for complex encapsulation processes, reducing the overall cost of the material. In the study conducted by the researchers, four different mortar compositions with the direct incorporation of nonencapsulated PCM were developed, and their physical, mechanical, and thermal properties were evaluated. Thermal tests, conducted based on temperature laws representative of the northern region of Portugal, showed a decrease in water absorption due to the partial occupation of the mortar pores by PCM. Although there was a slight reduction in mechanical properties due to the higher liquid–binder ratio, the thermal results demonstrated a significant decrease in extreme temperatures and heating and cooling needs. The mortar with 20% PCM proved to be the most effective in regulating interior temperature, contributing to the energy efficiency of buildings.

### 4.2. Immersion

In the immersion method, a porous construction material is immersed into liquid PCM, and it is absorbed due to capillarity. The main drawbacks of this method are leakage, construction incompatibility, and the corrosion of reinforced steel when incorporated with concrete elements, thereby affecting its service life [54]. Studies have shown that the immersion method consists of submerging PCMs to penetrate the materials’ porous structure through capillary action [55]. However, similarly to direct incorporation, this technique also faces the issue of PCM leakage. Such leakage can negatively affect both the mechanical strength and long-term durability of the construction elements. In both approaches, the PCM is directly integrated into the building material matrix.

Due to the corrosion issues affecting steel structures embedded in concrete used in civil construction, several studies assess the impact of PCMs on this process. In a study conducted by Cabeza et al. [22], the corrosion resistance of five commercial metals in contact with PCMs with melting temperatures between 5 and 29 °C was evaluated. The PCM used was the commercial TH29 (CaCl_2_·6H_2_O) and its mixture with MgCl_2_·6H_2_O (2:1 by weight), resulting in a new PCM with a melting point of approximately 23 °C. The objective was to test these materials for up to 500 days to assess the compatibility of the metals with PCMs. The results indicated that aluminum and steel should not be used with these PCMs due to corrosion issues.

### 4.3. Encapsulation

The encapsulation method may help prevent the leakage issues of PCMs and enhance their compatibility with the building structure. Encapsulation involves covering PCM with a shell to protect it from the external environment and prevent leakage. This method also plays a crucial role in increasing the heat transfer area and, consequently, improving the thermal conductivity of the PCM, ensuring its effective thermal storage performance [56].

PCMs can be encapsulated in two primary ways: microencapsulation, which involves enclosing the material in containers such as shells, tubes, channels, or thin plates; and microencapsulation, where tiny PCM particles are coated with a specialized polymeric layer [57]. For both techniques, the encapsulating material must meet essential criteria: it should effectively prevent leakage, retain the PCM’s thermal performance, remain chemically stable and non-reactive, be compatible with the PCM and its intended application, and offer structural integrity and safety during handling [58]. Additionally, it should accommodate the PCM’s volume changes during phase shifts, protect against environmental degradation, and possess high thermal conductivity and mechanical strength to maintain performance over time [59]. Materials like aluminum, copper, and stainless steel are frequently used for microencapsulation due to their excellent thermal conductivity, compatibility, and contribution to structural robustness of building components [60].

Due to the frequent presence of PCMs in their liquid state, it is essential to encapsulate these materials. The capsules must be corrosion-resistant, flexible, and mechanically robust [61]. Encapsulation prevents PCM leakage, increases the surface area-to-volume ratio—leading to improved heat transfer efficiency—and facilitates handling. There are three types of encapsulations: macroencapsulation, microencapsulation, and nanoencapsulation.

#### 4.3.1. Macroencapsulation

Macroencapsulation consists of enclosing specific amounts of PCMs, with masses ranging from a few grams to several kilograms, within larger containers such as spheres, panels, balls, or tubes. These containers not only store PCM but also offer mechanical support. Among the commonly used and cost-efficient options are plastic bottles, tin-coated metal cans, and mild steel containers. However, when mild steel is used, the inadequate application of internal or external protective coatings can result in significant corrosion problems, compromising both the container’s integrity and the PCM’s performance.

The macroencapsulation of PCMs in construction applications significantly contributes to more sustainable practices. It is important to highlight that the performance of macroencapsulated PCMs is influenced by various factors including geometry, thermal properties, and innovative designs.

Regarding geometry, the shape of the macrocapsules directly affects the heat transfer efficiency. Optimized designs can reduce phase change times and improve thermal regulation (Gonçalves et al. [23]). Additionally, Gonçalves et al. [23] emphasize that experimental studies indicate that increasing the contact surface of macrocapsules enhances heat transfer, while double-layer configurations may compromise performance.

To improve heat transfer rates in PCM applications, Laasri et al. [24] highlighted that the use of topology optimization techniques can lead to the development of more efficient structures, mitigating this limitation. Furthermore, the authors pointed out that macroencapsulation can be integrated into construction materials, such as bricks and wall panels, enhancing their thermal properties and energy storage capacity.

**Figure 6:**
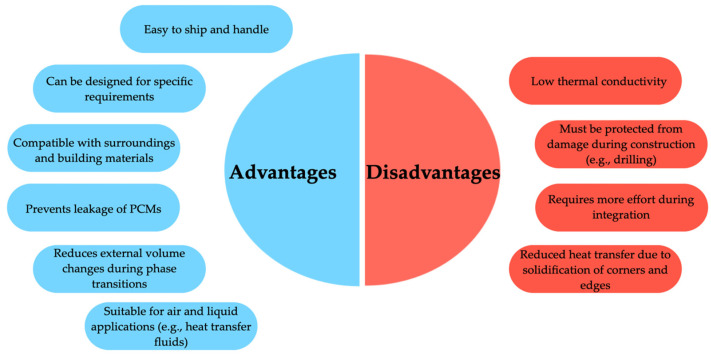
Principal advantages and disadvantages of the macroencapsulation technique.

The diagram shown in Figure 6 illustrates macroencapsulation and highlights the following advantages and disadvantages associated with this technique.

Castell et al. [62] applied the macroencapsulation technique using two different PCMs and examined their thermal behavior in combination with two types of building materials. The first setup used traditional clay bricks with PCM R-27, while the second involved alveolar (hollow) bricks combined with PCM BS-25. The results demonstrated a significant peak temperature reduction of up to 18 °C. Additionally, during the summer of 2008, the use of PCM-enhanced structures led to a 15% decrease in electrical energy consumption. This energy efficiency translated into a reduction in CO_2_ emissions by approximately 1000 to 1500 g/year/m^2^. Rathore et al. [63] outlined critical performance requirements for PCM containers used in macroencapsulation. These containers should possess high thermal conductivity, mechanical strength, and flexibility. Moreover, they must be non-toxic, resistant to corrosion and fire, and maintain both chemical and physical stability. Regarding the PCMs themselves, ideal candidates should have melting points aligned with the intended operational temperature range and must endure at least 5000 thermal cycles—comprising both charging and discharging—without significant degradation. A low vapor pressure within the application temperature range is also essential. Furthermore, PCMs should undergo minimal volume change during phase transitions, as significant expansion or contraction would necessitate larger, more complex containers.

In a related study, Al-Yasiri et al. [64] explored the application of microencapsulated PCMs in a layered roof structure composed of 4 mm of Isogam, 50 mm of concrete, and 8 mm of gypsum board. The researchers tested four configurations—Models A through D—with Model A serving as the control. In Model B, PCM was placed between the Isogam and concrete layers; in Model C, it was embedded within the concrete layer; and in Model D, it was located between the concrete and the gypsum board. The most effective thermal performance was observed in Model B, which exhibited reduced temperature fluctuations and a maximum indoor temperature drop of 9 °C compared to the reference model.

#### 4.3.2. Microencapsulation

Microencapsulation refers to the incorporation of small particles of PCMs, typically ranging in size from 1 μm to 1 mm, into various applications requiring temperature regulation—generally within a range of 10 to 80 °C. This technique offers several benefits: high thermal conductivity, the effective containment of PCM during phase transitions, ease of integration into construction materials, resistance to volume changes, and good chemical and thermal stability. The efficiency of heat transfer is enhanced due to the increased surface area relative to the unit volume of PCM. Despite these advantages, microencapsulation has some drawbacks. These include the high cost of production, potential negative impact on the mechanical properties of building materials, and limited convective heat transfer due to the structural rigidity of the encapsulated particles [65]. Microencapsulated PCMs are commonly found in spherical or irregular forms. There are two main approaches for producing microencapsulated PCMs. Chemical methods include techniques such as the following:

- Suspension polymerization, where particle coalescence, break-up, secondary nucleation, and monomer diffusion collectively influence the morphology and surface characteristic of the microcapsules [66];
- Dispersion polymerization, which requires careful control of factors such as the initiator, monomer, stabilizer concentration, and reaction time to achieve desired particle properties.

Other chemical processes include emulsions, in situ polymerization, interfacial polymerization, and polycondensation [67].

Physical methods are divided into the following:

- Physicochemical approaches, such as simple and complex coacervation, sol–gel encapsulation, and supercritical CO_2_-assisted techniques;
- Physicomechanical methods, including spray-drying, electrostatic encapsulation, and one-step processes.

Schossig et al. [13] evaluated the performance of microencapsulated PCMs in building materials such as plaster and walls. Their findings indicated that temperatures exceeding 26 °C could be effectively reduced. Similarly, Cabeza et al. [14] observed that in rooms without natural ventilation, the inclusion of microencapsulated PCM led to a 2 °C reduction compared to that without PCM.

A persistent challenge in microencapsulation has been the mechanical fragility of the PCM shell. Beyhan et al. [68] addressed this by developing robust microencapsulated PCMs (MPCMs) composed of a capric acid–myristic acid blend with two different copolymers of methyl and methacrylate. Two types of MPCMs (MPCM-1 and MPCM-2) were produced, with latent heats of 91.9 J/g and 97.3 J/g and melting ranges of 17.1–25.3 °C and 13.4–25.0 °C, respectively. Particle sizes ranged from 400 to 850 nm and 250 to 475 nm. The MPCMs retained thermal and chemical stability after 200 and 1000 thermal cycles, respectively. These materials were tested in geopolymer concrete walls to examine their response to factors such as solar radiation, wall thickness, and PCM concentration [69]. Results showed that increasing wall thickness and PCM content led to lower energy consumption and better thermal efficiency. However, under higher solar radiation, energy use increased, and efficiency declined. To optimize performance, MPCMs with melting temperatures near the average between indoor and outdoor temperatures are recommended. Testing in cities like Oslo and Madrid revealed better performance in Madrid, highlighting the influence of geographic location and wall orientation on efficiency.

Zhang et al. [15] explored MPCMs composed of gypsum and silica as the encapsulating material, with paraffin serving as the PCM. At a pH of 2.5, the MPCMs exhibited excellent morphology and dispersion. However, increasing MPCM concentrations led to reduced thermal conductivity and compressive strength due to incompatibilities between the organic PCM and inorganic gypsum. Acceptable mechanical performance was maintained at MPCM concentrations of around 10%.

Balapour et al. [70] examined how mixing speeds affect thermal efficiency. At low mixing speeds (55 rpm), thermal efficiency dropped by 5.5–7.5%, while at high speeds (165 rpm), it fell by up to 50%. The study also tested cement mixtures with MPCM particles of two different scales (10^−2^ m and 10^−3^ m), showing thermal efficiency reductions between 32% and 34% depending on the mixing speed. The main factors contributing to the loss in thermal and mechanical performance were the interaction between sulfate groups (SO_4_^2−^) and MPCMs, as well as the soft nature of the encapsulated material. To overcome this drawback, it is recommended to select materials with a lower sulfur content or to develop an improved encapsulating material.

#### 4.3.3. Nanoencapsulation

Nanoencapsulation involves embedding extensive quantity of PCM particles at nanometric scales, typically ranging from 1 nm to 1 μm. Due to their extremely small size, these particles possess a very high surface area-to-volume ratio, which significantly enhances heat transfer efficiency. Similarly to microencapsulation, nanoescapsulation can be achieved through various chemical processes such as suspension polymerization, emulsion techniques, interfacial polymerization, in situ dispersion, and others [71].

Zhu et al. [30] developed an innovative single-step process for synthesizing polymer–silica hybrid shelled nanoencapsulated PCMs (Nano-PCMs). Their method combined the interfacial hydrolysis polycondensation of alkoxysilanes with the subsequent radical polymerization of vinyl monomers. The study further investigated the effects of incorporating additional components and modifying the SiO_2_ content on the performance of the nano-PCMs. The results demonstrated thermal reliability, improved thermal conductivity after the addition of certain materials, and satisfactory mechanical properties, which were further enhanced with the incorporation of specific components.

Valizadeh et al. [31] demonstrated that the use of PCM nanocapsules reduces the need for floor heating and cooling. Furthermore, Maleki et al. [32] used (NPCM) *n*-dodecanol as a PCM and incorporated CuO and PMMA nanoparticles in the encapsulation process. The addition of CuO contributed to increased thermal conductivity and a reduction in rapid cooling rates, while the encapsulated nanoparticles helped minimize volume changes during melting and solidification, as well as enhance the heat transfer rate. The latent heat capacity was measured at 148.88 J/g, and the encapsulation efficiency was approximately 72.3%. The results indicated good thermal reliability and improved thermal conductivity.

Recently, Aidi et al. [33] investigated the impact of adding nanoparticles on the performance of PCMs, focusing on three types: N-octadecane, N-eicosane, and N-hexadecane. With the incorporation of alumina and copper oxide and CuO nanoparticles, the researchers found significant improvements in thermal conductivity and energy absorption. The results showed that higher concentrations of nanoparticles delayed peak temperature fluctuations by up to 1.5 h and influence viscosity. Among the PCMs tested, N-hexadecane proved to be the most effective for enhancing thermal comfort in winter, while N-octadecane stood out for its favorable melting point properties. The authors suggested that findings like these could be particularly useful for reducing wall thickness in conventional buildings, thereby decreasing the total construction volume and, consequently, increasing the available usable area.

### 4.4. Shape-Stabilized PCM

Shape-stabilized phase change materials (SSPCMs) are innovative solutions to enhance thermal energy storage (TES) systems, addressing the limitations of traditional PCMs. By incorporating support matrices, such as polymers or foams, SSPCMs mitigate issues like leakage and low thermal conductivity, making them suitable for various applications. This method stabilizes PCM within a support matrix, providing better thermal conductivity, high specific heat capacity, and maintaining shape over many phase transition cycles. This technique consists of combining phase change materials (PCMs) with high-density substances—such as high-density polyethylene, styrene, and butadiene—along with support materials like diatomite, expanded graphite, silica fume, kaolin, and recycled glass. The mixture is subjected to high-temperature processing and subsequently cooled, resulting in the formation of stable, solid composites. These materials exhibit the following characteristics [72]:

- Elevated specific temperature.
- Satisfactory thermal conductivity capacity.
- Resistance to shape changes during phase transition.
- Stability in performance after extended periods of heat cycling.
- No need for packaging.
- Capability to be employed as internal linings in buildings.
- A PCM ratio reaching up to 80%.
- The prevention of leakage even when the material is exposed to temperatures above the PCM’s melting point.

The vacuum impregnation method has been proven effective in enhancing the retention capacity of porous construction materials. Among the materials used, polyethylene glycol (PEG) stands out due to its high thermal storage capacity, appropriate melting temperature, minimal volume variation during phase transitions, and absence of supercooling. Moreover, PEG is recognized for being non-toxic and chemically stable over extended periods [73]. In a study conducted by Sarı [17], natural clay and gypsum were impregnated with PEG at concentrations of 22 wt.% and 18 wt.%, respectively, using the vacuum impregnation technique. Differential scanning calorimetry (DSC) revealed melting temperatures of 10.85 for the clay composite and 10.55 °C for the gypsum-based material. The corresponding latent heat values were 28.79 kJ/kg and 24.18 kJ/kg. Additionally, the gypsum–PEG composite achieved internal temperature reductions of 2.08 and 1.47 °C after 60 min and 120 min of exposure to elevated temperatures. For a more cost-effective solution, Sarı et al. [18] also explored the incorporation of PEG into low-cost pumice, achieving a loading of 54 wt.%. The resulting material demonstrated a thermal capacity of 98.39 kJ/kg and a melting temperature of 8.80 °C. The composite PCM also demonstrated good thermal durability and chemical stability. Nonetheless, the polyethylene glycol-based PCM still exhibited leakage and thermal conductivity issues. To address this limitation, Sarı et al. [19] used carbon nanotubes to increase thermal conductivity by 73% to 93%, which resulted in a reduction in the heat storage ability and release period. The implementation of carbon nanotubes also led to additional benefits, such as a significant increase in thermal storage capacity by 5–31%. Moreover, the leakage level decreased and improved thermal and chemical stability were observed.

Hekimoglu et al. [20] investigated the enhancement of thermal conductivity in PCM composites by incorporating advanced carbon-based nanomaterials such as carbon nanotubes, carbon nanofibers, and graphene nanoplatelets. In their study, PEG was embedded within a silica foam matrix, resulting in a composite with a thermal storage capacity of 55–56 kJ/kg and a melting temperature in the range of 14–15 °C. When functionalized with 8 wt.% of the respective nanomaterials, the PEG-based composites exhibited significant improvements in thermal conductivity—136% for the graphene nanoplatelets, 106% for the carbon nanotubes, and 85% for the carbon nanofibers. To reduce material costs, the authors also explored the use of activated carbon derived from apricot kernel shells as an alternative thermal conductivity enhancer. With a 70 wt.% loading of PEG, this composite achieved a thermal conductivity approximately 1.75 times higher than the base PEG PCM.

In another study, Jeong et al. [74] developed a bio-based shape-stabilized PCM, selected for its affordability, high latent heat, and low vapor pressure during phase transition. To address issues of leakage and suboptimal heat transfer, boron nitride was added to improve both structural stability and thermal performance.

Similarly, Yang et al. [75] tackled the challenges of leakage and poor thermal conductivity by formulating a novel ceramsite-based composite PCM, which exhibited a latent heat of 205.9 kJ/kg and a melting point of 21.3 °C. The composite demonstrated excellent thermal and chemical stability, as well as improved mechanical strength, showing a 15% performance increase with the inclusion of LA-SA/Al_2_O_3_/CPCM.

Li et al. [76] used paraffin/mesoporous silica shape-stabilized PCM, which also showed good thermal and chemical stability as well as thermal conductivity. They overcame the problem of storage time, which decreased by 34% due to the addition of mesoporous silica, although the thermal conductivity decreased.

**Figure 7:**
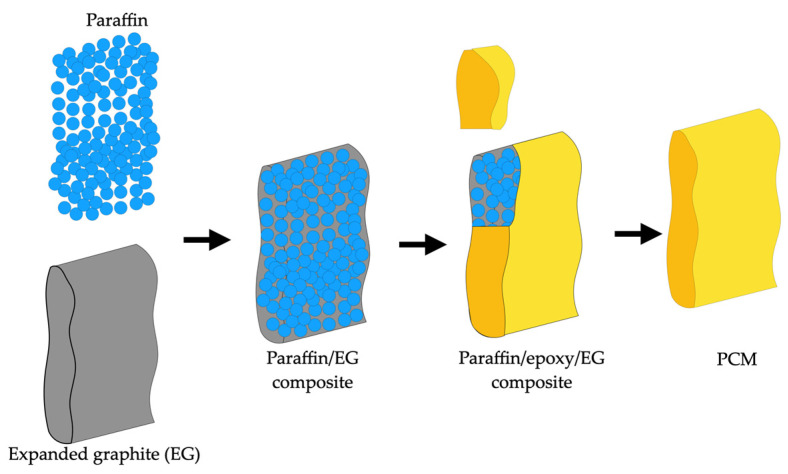
The preparation process of the new ternary material. Adapted from [63].

A novel PCM with a compact three-dimensional network structure and long cycle life was proposed by Wang et al. [77]. In this material, energy could be obtained from the thermal motion of surrounding molecules in the environment. The composite was made from paraffin mixed with an impact structure and shape-stabilized epoxy at 70 °C. Thermal analysis results showed that the paraffin was uniformly distributed in the polymer matrices, and there was no paraffin leakage during the sample preparation. Mechanical tests demonstrated excellent performance in the composites, with mechanical properties increasing as the epoxy content increased. Moreover, when paraffin and epoxy were prepared in a 1:1 mass ratio, the composite could be cut into any shape while maintaining good mechanical properties and thermal stability without leakage. The results indicated that this new composite PCM has good mechanical properties and thermal stability, making it significantly applicable in the long-term effective use of thermal energy storage. The process of preparing the ternary material obtained by the researchers is shown in Figure 7, where the epoxy not only provided a flexible encapsulated structure for the paraffin/EG composite to prevent leakage of the melted paraffin but also maintained a highly compact network morphology.

### 4.5. Form-Stabilized PCM

Form-stabilized PCM is also the result of an advanced method of incorporation. It is a specific type of composite material that retains the maximum amount of one or more types of PCM while preventing leakage at melting temperatures. Although the latter two methods are expensive to implement, they are the most reliable among all alternatives. Reliability means that the PCM cycles (melting/solidification) can be repeated with high performance without degradation, a crucial feature for long-term applications that require consistent efficiency, such as in buildings [78].

Form-stabilized PCMs offer several key advantages that make them highly efficient in thermal energy storage. One of the main advantages is leakage prevention, where the stabilizing matrix effectively prevents the PCMs from leaking when they melt [79]. Additionally, these materials demonstrate shape stability, meaning that the composites maintain their structural integrity even when the PCMs melt [80]. Another significant advantage is enhanced thermal conductivity, as the supporting material improves the PCMs’ ability to conduct heat, thereby facilitating more efficiency heat transfer [40]. Furthermore, form-stabilized PCMs exhibit better mechanical stability compared to pure PCM, making them stronger and more durable for practical applications [43]. These features collectively establish form-stabilized PCMs as an innovative and practical solution for thermal control systems.

Form-stabilized PCMs also have a wide range of applications across various industries due to their unique thermal properties. In the construction sector, they are used in building materials such as walls, ceilings, and floors to regulate indoor temperatures, reducing the need for heating and cooling systems and significantly improving building energy efficiency [80]. In the textile industry, form-stabilized PCMs are incorporated into high-performance clothing to provide thermal comfort by absorbing through excess body heat and releasing it as needed, thereby enhancing wearer comfort [40]. Additionally, these materials play a crucial role in thermal energy storage systems, particularly in industrial processes and solar energy applications, where they capture and store heat energy for later use, improving energy efficiency and lowering operating costs [43]. Moreover, form-stabilized PCMs are increasingly being used in electronics cooling systems, where they help regulate heat in electronic components, preventing overheating and ensuring optimal performance [79].

Form-stabilized PCM (FSPCMs) often incorporate highly porous structures, such as carbon nanotubes and biomass-derived materials, to prevent leakage during phase transitions and enhance thermal conductivity [81,82]. Lamastra et al. [81] developed FSPCM composites using carbon nanotube–decorated diatomite (CNT/DE) and CNT sponges (CNS), demonstrating high thermal reliability and a reduction in cooling requirements in buildings.

On the other hand, Prabhu et al. [82] conducted a comprehensive review on biomass-derived FSPCMs (BFSPCMs), highlighting the impact of these structures on the efficiency of solar thermal energy systems (STES). The study indicated that carbonization at 900 °C and vacuum impregnation are viable techniques for synthesizing these materials, improving thermal performance, temperature regulation, and waste heat recovery.

### 4.6. Encapsulation Performance Parameters

The material of the shell plays a crucial role in the heat transfer performance and mechanical strength of encapsulated PCMs. A shell composed of a material with high mechanical strength and thermal conductivity improves the performance of the thermal energy storage system and increases the number of thermal cycles that the encapsulated PCM can withstand. According to Salunkhe et al. [83], an optimal shell material should possess sufficient structural and thermal strength to endure the phase change process, be resistant to leakage, not react with the enclosed PCM, and have high thermal conductivity to enhance heat transfer between the PCM and its surroundings. The selection of the PCM, the shell material, and the encapsulation method is essential, as these factors significantly impact the overall performance of the encapsulated PCM. The type of PCM determines the most suitable shell material in terms of chemical compatibility and the encapsulation methodology. Additionally, the working temperature of the PCM directly influences the choice of shell material. Polymer-based shells are suitable for low-to-medium working temperatures, while metallic and ceramic shells can be used at low-to-high temperatures. Among them, ceramic shells offer the highest resistance to high temperatures, as metallic shells are susceptible to corrosion and material incompatibility under extreme conditions. High-temperature applications increase the likelihood of chemical incompatibility, thereby narrowing the range of possible shell–PCM pairings. Most high-temperature microencapsulated PCMs are produced using metallic shells due to their superior mechanical strength and thermal stability, despite the potential for corrosion. Ceramic shells have also been studied and are more affordable; however, their porous nature increases the risk of leakage.

Jacob and Bruno [84] compared the thermal conductivity of encapsulated PCMs with different shell materials, including metallic [85], polymeric [86], and inorganic [87] options for high-temperature energy storage. Among the materials evaluated, steel, nickel, silica, calcium carbonate, and titanium dioxide demonstrated the highest potential for future use. Despite the poor thermal conductivity and chemical incompatibility of polymeric shells at high temperatures, more than 50 different polymers were studied as shell materials and were valuable in most cases [88].

To address the limitations of polymeric shells, researchers have explored inorganic materials that offer higher thermal conductivity and mechanical strength. Calcium carbonate [89], titania [90], and silica microcapsules shells have been proposed using interfacial or in situ polymerization techniques. Silica capsules are the most extensively studied, with paraffin–silica micro- and nanocapsules synthesized by spray-drying, interfacial polymerization, interfacial polycondensation, and emulsion polymerization, among other methods [91]. However, the development of inorganic shell materials remains strongly dependent on the core/shell encapsulation methodology. Limited research has been conducted on the encapsulation of high-temperature PCMs, such as molten salt and metals. Some studies have investigated molten salt encapsulation using ceramic precursor resin [92], silica [93]_,_ or alloys like zinc and aluminum–silicon with alumina shells [94]. Smaller microcapsule sizes and narrower size distributions improve heat transfer efficiency by increasing the surface area. Efficient encapsulation with an appropriate core content enhances heat capacity, while shell thickness acts as a thermal barrier, influencing both heat transfer and mechanical stability.

Recent technologies aim to produce PCM microcapsules with smaller diameters to reduce the degree of supercooling [95]. The crystallization temperature of melamine–formaldehyde (MF)/n-dodecane microcapsules is directly affected by capsule size [96]. For microcapsules with diameters between 5 and100 μm, supercooling increases as size decreases, but capsules with diameters between 100 and 1000 μm exhibit similar degrees of supercooling. Larger microcapsules are less suitable for building applications as they tend to rupture during mixing [97]. To mitigate this issue, a nucleation agent is often mixed with PCM before encapsulation.

Yamagishi et al. [98] found that the supercooling temperature of microencapsulated n-dodecane and n-tetradecane decreased with smaller capsule diameters (5–100 μm). Sukhorukov et al. [99] showed that mechanical properties improved when transitioning from micro-scale to nano-scale encapsulation. Nanocapsules with diameters of 10 nm exhibited higher mechanical strength and deformation resistance than 10 μm microcapsules, making them more suitable for long-term operation despite higher pumping power requirements. Nanoencapsulation also offers higher cyclability and faster heat transfer than micro-sized capsules, increasing interest in future developments [100].

Nahak et al. [101] modeled a 3D unstructured packed bed system consisting of spherical encapsulated PCMs with different sphere sizes. The novelty of their work lay in resolving the geometry of the capsules and the flow between them, even with unequal capsule sizes arranged irregularly. By adjusting the mean and standard deviation of sphere size, different distributions were generated. The authors reported that doubling the mean sphere radius while maintaining a constant standard deviation increased the melting rate and melting time by 29.4%. The effect of the standard deviation was less significant compared to that of the mean radius.

Baruah et al. [102] developed a numerical model simulating PCM melting due to the heat transfer fluid flow around the capsule, incorporating a metal foam structure at the pore-scale level. The model, based on the enthalpy method, included four phases: the heat transfer fluid, metal, solid PCM, and liquid PCM. Their parametric analysis confirmed that the metal foam’s internal structure significantly influenced the melting pattern and energy storage capacity due to its large surface area. Lower capsule sizes accelerated melting but reduced the energy stored per capsule. This limitation can be addressed at the system level by increasing the number of capsules or optimizing capsule size. Shell thickness also influences phase change duration and energy storage capacity. Thicker shells reduce phase change time and enhance energy storage performance.

Encapsulation has been proven effective in preventing PCM leakage, reducing environmental reactivity, improving heat transfer rates, and controlling volume changes during phase transitions. For polymer-based encapsulated PCMs (EPCMs), the mass ratio of the polymer influences the encapsulation ratio and leakage rate [103]. Increasing the polymer content decreases the encapsulation ratio, while reducing it weakens the shell, increasing the risk of defects during polymerization [66]. Weak shell regions are prone to cracking under pressure, negatively affecting the leakage rate and posing a challenge for improving both encapsulation efficiency and leakage prevention [104].

Graphene oxide, with an elastic modulus of approximately 208 GPa, has been explored as a reinforcing material to enhance the mechanical strength of polymer shells and reduce leakage. Valizadeh et al. [105] reported that incorporating graphene oxide into polystyrene shells significantly reduced the paraffin leakage rate of nanocapsules—by approximately 86%, 83%, and 81% after 30, 60, and 120 h, respectively. The graphene oxide nanosheets formed a stable emulsion, covering the paraffin microspheres during emulsification and improving shell integrity.

The graphene oxide (GO) nanosheets were produced as a stable emulsion that covered the paraffin microspheres during emulsification. The GO nanosheets appeared to be located at the interface between the shell and the core, forming a protective barrier around the paraffin nanospheres. Once the melting point of the core was reached, the paraffin molecules migrated through the boundary and the interlayer of the GO nanosheets, increasing the path length for leakage due to the dual protective layers. If holes or cracks were present, the leakage would persist until the paraffin was entirely released. The leakage rate of nanoencapsulated phase change materials (NEPCMs) with or without GO increased over time.

Kumar et al. [106] conducted experimental work on nanoencapsulated molten salt for thermal storage applications. They performed leakage tests to evaluate the strength of the shell and its ability to prevent leakage during the solid–liquid phase transition. The samples were heated to 260 °C, and after reaching the melting temperature, no liquid formation was observed in the encapsulated samples, indicating that the phase transition occurred within the silica and titania shells without leakage. The leakage rate test also confirmed the shell’s ability to withstand pressure variations during phase transition.

Min et al. [12] developed an innovative stereotyped PCM with a low leakage rate for green building energy storage. Their research involved binary paraffin wax produced through the hot fusion of solid and liquid paraffin, polypropylene (PP), and waste foamed concrete porous material to create a composite PCM. A waste foamed concrete–polypropylene–paraffin wax stereotyped PCM (FCPCM) with a low leakage rate and good stability was prepared using melt blending and vacuum adsorption methods to meet the energy storage demands of buildings. Morphological stability and heat storage/release performance tests were conducted to evaluate the thermal behavior of the energy storage concrete. The 24 h leakage rate remained below 1%, approximately 44% lower than that of FCPCM using PP as the support material. Furthermore, the thermal performance test showed that, compared to traditional concrete, energy storage concrete exhibited higher heat storage density and improved temperature regulation, offering potential energy savings for buildings.

## 5. Nano-Enhanced PCM Composites

The integration of nanomaterials with high thermal conductivity into PCMs can significantly improve their thermophysical properties [107,108]. Among the different nanomaterials, carbon-based materials have shown superior performance. Many studies have highlighted the effectiveness of incorporating carbon-based nanoparticles into paraffin wax (PW)-based phase change materials to enhance their thermal properties. Fan et al. [109] investigated the influence of multi-walled carbon nanotubes (MWCNTs), carbon nanofibers, and graphene nanoplatelets on the thermal characteristics of paraffin wax-based PCMs. All composite PCMs exhibited higher thermal conductivity than pure paraffin wax; however, this improvement reduced the latent heat storage capacity of the composite. The composite PCM with 5 wt.% graphene nanoplatelets achieved the highest thermal conductivity value, approximately 164% higher than that of the pure paraffin wax.

Shi et al. [110] prepared composite PCMs by mixing different weight concentrations of exfoliated graphite with paraffin wax. The 10 wt.% graphite solution exhibited the highest thermal conductivity of 2.7 W/m·K, significantly higher than the thermal conductivity of the paraffin wax (0.25 W/m·K). However, the authors did not report on the temperature dependence of thermal conductivity or the effect of concentration on heat capacity, melting temperature, and latent heat. Similarly, Li [111] examined the effect of nano-graphite on paraffin wax with a melting point of 30 °C. The inclusion of 10 wt.% nano-graphite increased thermal conductivity by 641% but lowered the latent heat storage capacity by 13.1%. The researcher also found a negligible change in melting temperature with increasing concentration.

Warzoha et al. [112] enhanced the thermal properties of paraffin by mixing it with graphite nanofibers. The thermal conductivity in the solid and liquid phases improved by 386% and 167%, respectively, at a concentration of 11.4%. However, the specific heat in the solid and liquid phases decreased by 63% and 39%, respectively. Additionally, the latent heat storage capacity decreased by 11%, and the melting point increased by 11%. Another study mixed paraffin wax with 1%, 5%, and 10% graphene nanoparticles to improve thermal properties. The composite PCM demonstrated enhanced thermal conductivity and stability, with the 10 wt.% graphene nanoparticle solution exhibiting around 40% higher storage capacity.

Alumina and carbon black nanoparticles were also used to enhance the thermal properties of paraffin wax RT20 and RT25. The latent heat storage capacity of the alumina-based composite PCMs was found to be higher than that of pure paraffin wax, whereas it was slightly reduced for the carbon black-based composite PCMs due to the lower heat storage capacity of carbon black compared to alumina. Nevertheless, the thermal conductivity of the carbon black-based composite PCMs was higher than that of the alumina-based composite PCMs [113]. Despite these improvements, a clear trend for PW-based composite PCMs remains difficult to establish, which hinders the development of a consistent guideline for photovoltaic (PV) cooling applications.

Many studies have highlighted the superiority of both MWCNTs and graphene nanoplatelets in improving the thermal properties of PCMs [114,115,116]. Paraffin wax, due to its suitable melting temperature and latent heat storage capacity, remains a promising choice for PV thermal regulation and latent heat storage. However, its low thermal conductivity has prompted researchers to incorporate high conductivity nanofillers, particularly carbon-based materials. Despite these efforts, no conclusive direction has been established. Therefore, further studies are required to understand the composition and behavior of nanofiller-incorporated PCMs and their potential for improving photovoltaic thermal management while utilizing stored heat for other useful applications in hybrid PV/thermal systems.

Abdelrazik et al. [117] selected MWCNTs and graphene nanoplatelets to enhance the thermal performance of paraffin wax. Different weight concentrations of nanomaterials were mixed with paraffin wax to prepare two distinct sets of nano-enhanced PCMs. Most of the paraffin wax–MWCNT samples showed higher thermal conductivity than the paraffin wax–graphene nanoplatelet samples. However, the paraffin wax–graphene nanoplatelet samples exhibited higher specific heat values. At a 5 wt.% concentration, the paraffin wax–MWCNT showed an increase in thermal conductivity of around 14%, compared to 3.8% for the paraffin wax–graphene nanoplatelet sample at 25 °C. At the same concentration, the specific heat of the PW–GNP was higher than that of the PW–MWCNT by 6.7%. Furthermore, after repeated thermal cycling, the nano-PCMs displayed good thermal stability.

Yan et al. [118] used expanded graphite as a framework to encapsulate erythritol and form a composite PCM using a physical adsorption approach. The erythritol–expanded graphite composite PCMs exhibited a thermal conductivity of 5.97 W/m·K, compared with 0.6 W/m·K for pure erythritol, owing to the honeycomb structure and high thermal conductivity of expanded graphite. The latent heat of fusion and solidification reached 20.52 J/g and 239.52 J/g, respectively, indicating high thermal energy storage and release rates. The compressive strength also increased to 40.41 MPa, demonstrating improved mechanical properties.

Yan et al. [119] prepared expanded graphite–polyethylene glycol (PEG) composites via a physical adsorption method and analyzed the relationship between the mass percentage of expanded graphite and the microstructure, phase change enthalpy, supercooling degree, and energy conversion properties. The composite PCM exhibited exceptional thermal conductivity (2.48 W/m·K, seven times higher than that of PEG) and outstanding melting enthalpy (161.4 J/g). Ouikhalfan et al. [120] produced composite PCMs with up to 2 wt.% of hexagonal boron nitride, achieving a peak thermal conductivity increase of 30% compared to PEG4000.

Advincula et al. [121] encapsulated stearic acid in graphene oxide nanosheets, which prevented leakage during phase transition (encapsulation rate of up to 85%) and enhanced thermal conductivity. Luo et al. [122] demonstrated elevated thermal conductivity (121%) and excellent photo-thermal conversion performance by using epoxy resin as an encapsulation material, PEG as the PCM, and 4 wt.% silver-modified expanded graphite as a thermal conductivity enhancer. Hirschey et al. [123] studied the influence of expanded graphite, milled expanded graphite, and graphite nanosheets on the thermal conductivity of sodium sulfate decahydrate composites. Adding 25 wt.% expanded graphite led to a 583% increase in thermal conductivity.

Aktay et al. [124] measured the thermal conductivity of nitrate salt/graphite lamellar composites from room temperature to over 50 °C above the melting point, confirming an 11% decrease in thermal conductivity beyond the melting point. Yu et al. [125] used the laser flash method to measure the thermal conductivity of expanded graphite-chlorinated salt–silica composites from room temperature to 700 °C. Xiao et al. [126] measured the thermal conductivity of nitrate salt–expanded graphite between 20 and 120 °C, observing a slight decrease with increasing temperature. Ling et al. [127] found that the thermal conductivity of paraffin–EG composites nearly doubled during the phase change temperature range but remained almost constant at other temperatures.

Yang et al. [128] studied the thermophysical characteristics of paraffin from room temperature to 80 °C, finding a nonlinear decrease in thermal conductivity near the melting point. Guo et al. [129] synthesized microsphere structures by embedding PCMs in graphene and measured thermal conductivity at 25 °C and 80 °C. The low thermal conductivity near the melting point was attributed to the variation in specific heat with temperature.

Overall, the thermal conductivity of composite PCMs varies with temperature and requires more detailed investigation, particularly near the melting point, to provide consistent data for improving the thermal performance of low-temperature organic PCMs.

## 6. Economic Analysis of PCMs

The widespread use of PCMs in building applications involves high upfront costs, which may discourage developers and homeowners from adopting them. Additionally, concerns about leakage and long-term durability raise further doubts about their viability, leading to greater investment caution. To offset these initial costs and encourage the adoption of PCM-integrated buildings, a detailed cost–benefit analysis is essential to assess long-term financial savings from reduced energy demands relative to the initial investment. Exploring alternative, cost-effective PCMs and providing financial incentives could also improve the economic feasibility of PCM-based solutions.

Konstantinidou et al. [130] evaluated the life cycle and life cycle cost implications of integrating PCMs in office buildings, assessing their economic and environmental performance through life cycle analysis (LCA) and life cycle cost analysis (LCCA). Their study built on a previous multi-objective optimization analysis of building envelopes, which considered cooling load requirements and thermal comfort as optimization targets. This work extended the previous study by analyzing the economic and environmental impacts of the optimized solutions.

The study first examined whether the environmental impact reduction from operational energy savings compensated for the increased impact from PCM production. The LCA results showed that the overall life cycle impact of the two office units analyzed decreased despite a significant increase in the construction phase impact, due to the high proportion of energy use in the overall impact. However, the LCCA indicated that the energy savings during operation were insufficient to offset the increased life cycle cost (LCC) from construction. In both undivided and subdivided office units, construction and dismantling costs increased significantly compared to the reference case. Material costs led to an overall increase in the discounted LCC of 4.1% for the undivided office unit and 11% for the subdivided unit. When considering a 2% increase in energy and water prices, the real LCC increased by only 0.9% for the undivided unit but by 6.9% for the subdivided unit.

Based on this assessment, PCM integration is economically unfeasible due to high purchase and installation costs, particularly for subdivided office units, which require more PCM. However, the positive impact of PCMs on indoor thermal comfort should not be overlooked, even though it is difficult to quantify economically. The key question remains whether the market is ready to adopt PCMs in commercial buildings. The study suggested that PCM applications could become economically viable if energy prices increase significantly or if PCM costs decrease by approximately 40%, thereby reducing the initial investment.

Saafi and Daouas [131] conducted a sensitivity analysis of the economic benefits of PCM applications and found that cost effectiveness is highly sensitive to inflation and discount rates. They recommend using the dynamic payback period (DPP) methodology, which accounts for the time value of money, to determine the payback period and economic benefits.

Mi et al. [132] studied the effect of PCMs on the energy consumption of office buildings in five Chinese cities (Shenyang, Zhengzhou, Changsha, Kunming, and Hong Kong), representing distinct climatic regions. They simulated energy performance using Energy Plus^®^ over one year. For the economic analysis, they used the static payback period (SPP) and dynamic payback period (DPP), considering different discount rates.

The SPP represents the time required to recover the investment from annual net income (income minus expenditure) and is expressed as

(1) $$SPP=\frac{C_{PCM}}{S}$$

where $C_{PCM}$ is the initial investment in *PCM* and *S* is the annual income from energy savings. However, *SPP* does not account for the time value of money, which leads to underestimating the actual payback period.

The *DPP*, which includes the time value of money, is defined by

(2) $$\sum_{t=0}^{DPP}{S(1+r)}^{-t}-C_{PCM}=0$$

where *r* is the discount rate. Including the time value of money typically results in a longer and more accurate payback period than the *SPP*.

According to the National Bureau of Statistics of the People’s Republic of China, energy prices increased at an average annual rate of 4.01% between 1998 and 2014 [133], and long-term energy price growth is expected to continue in the coming decades [134]. A consistent upward trend in energy prices would enhance the economic benefits of PCM applications in buildings.

To ensure reliable research results, the energy price was assumed to remain constant in the study. Therefore, if the economic analysis of PCM applications showed positive results under constant energy prices, any future rise in energy prices would lead to greater financial benefits from energy savings in real projects.

The economic analysis revealed that energy savings during the summer alone were insufficient to recover the investment in office buildings located in different Chinese cities. However, when considering energy savings during both summer and winter, PCM applications in Shenyang, Zhengzhou, and Changsha showed promising economic value, making the investment attractive. Conversely, at current prices, PCM investments in Kunming and Hong Kong were not recoverable and did not offer economic benefits.

## 7. Applications for PCMs in Construction Materials

The current section describes the background and main advances of the integration of PCMs in construction materials, namely concrete, wallboards, and bricks. This part of the overview will present the main techniques explored for the inclusion of the PCMs and the benefits of this inclusion in properties like mechanical strength and thermal regulation. Finally, the energy consumption savings of these technological solutions are also addressed in cases where the specified construction materials are inserted into innovative green buildings.

### 7.1. Background and Advances in PCM-Integrated Concrete

Although various types of materials can be considered and used as PCMs, not all of them can be incorporated into construction components. The ever-growing demand in the global real estate sector has encouraged the integration of these materials to enhance the thermal and environmental performance of new buildings. Concrete remains the preferred construction material worldwide [13,134,135], which has intensified scientific efforts to develop better solutions and improve the PCMs used in the sector. The PCMs are integrated into concrete to enhance its latent heat storage capacity, assisting in indoor temperature regulation by absorbing and releasing heat during the phase transitions. However, this integration presents challenges, such as PCM leakage, reduced mechanical strength, and encapsulation durability.

The techniques explored for PCM integration into concrete generally involve material encapsulation, which is essential to prevent leakage and maintain the structural integrity of the new composite. Sari et al. [136], for example, demonstrated that using natural zeolite as a host matrix for dodecanol-based PCM effectively eliminated the leakage concerns, while preserving the mechanical properties and efficiently managing thermal performance in cementitious mortars.

**Figure 8:**
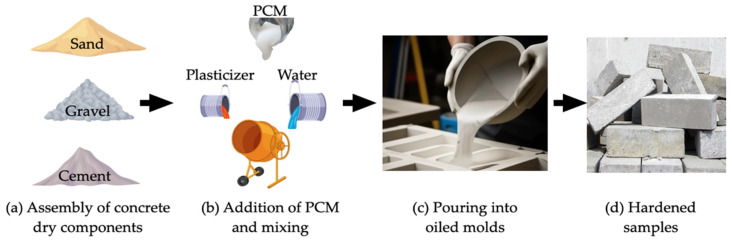
A schematic representation of the preparation of PCM-incorporated concrete used by D’Alessandro et al. [138].

In addition to using zeolites as an encapsulation strategy, other methods have been investigated to improve PCM incorporation in concrete. Ubertini et al. [137] practically evaluated different encapsulation techniques, comparing both traditional micro-PCM-capsules (micro-PCM) and the more innovative macro-PCM-capsules (macro-PCM), both with a phase transition temperature of 18 °C. Figure 8 schematically illustrates the preparation procedures of PCM-incorporated concrete used by D’Alessandro et al. [138].

The results confirmed the thermal benefits of PCM incorporation and indicated that its addition reduced the density of concrete by approximately twice the weight of the incorporated PCM. Although the average compressive strength decreased with increasing PCM content, the coefficient of variation in this property was not significantly affected. Additionally, for a 1% by weight content of micro-PCM and macro-PCM, an increase in characteristic compressive strength was observed, suggesting a filling effect of the PCM and a positive thermal interaction with cement hydration products. The study also demonstrated that macro-PCM-capsules could act as aggregates, contributing to the structural and thermal multifunctionality of concrete, making it a promising material for applications in energy-efficient building envelopes.

PCM can also be incorporated into aggregates, such as surfactant-based PCM aggregates, which are lightweight and do not exhibit leakage or strength reduction during phase transitions, as demonstrated in the work of Song et al. [139]. In that study, the authors developed leak-proof PCM artificial aggregates using an emulsion technique, a cement-free binder, and cold pelletization. Differential scanning calorimetry (DSC) and thermogravimetric analysis (TG) results confirmed that this approach significantly increased the PCM content in the aggregates without leakage. To produce the aggregates, the researchers employed a PCM–water emulsion stabilized by the non-ionic surfactant Pluronic P123, used as mixing water in the synthesis of cement-free artificial aggregates, activated with Ca (OH)_2_-Na_2_CO_3_ and consolidated by cold pelletization. The concrete containing these aggregates exhibited improved thermal performance, with a compressive strength of 18.1 MPa at 28 days and a more stable temperature profile, indicating its potential for applications in energy-efficient buildings by reducing internal thermal fluctuations.

Another possibility is impregnation in mortar, where PCM is incorporated into mortar mixtures. An example of this is PCM-impregnated cork granules, which improve thermal properties while maintaining acceptable mechanical performance [140].

The benefits of PCM-enhanced concrete have been widely demonstrated in the recent literature, highlighting three main aspects: (i) improved thermal regulation, as evidenced in the study by Niall and West [141], in which PCM concrete composites reduced indoor air temperature by up to 16% in temperate climates; (ii) increased energy efficiency, since PCM incorporation reduces the need for auxiliary heating and cooling systems, contributing to energy savings and mitigating internal temperature peaks, as demonstrated by Sarı [136] and Niall and West [141]; (iii) sustainability, a crucial factor in the face of global temperature fluctuations [142]. In the study by Ziga-Carbarín et al. [142], for example, PCMs were incorporated into Portland cement partially replaced with fly ash and nano-silica. This approach delayed cement hydration and improved thermal insulation, reducing temperatures by an average of 4 °C. Additionally, the combination of materials contributed to decreasing carbon dioxide penetration and water absorption, increasing the durability of the composite.

Although PCM-enhanced concrete offers promising benefits for energy efficiency, cost reduction, and sustainability, certain challenges must be addressed, among which the following stand out: (i) mechanical strength, as PCM incorporation can reduce the structural strength of concrete, especially at higher PCM concentrations (this effect requires a balance between improving thermal performance and preserving the composite’s mechanical properties [143,144]); (ii) variability in thermal performance, as the effectiveness of PCM concrete depends on environmental conditions (while it performs optimally in temperate climates, its efficiency may be compromised in high-temperature regions, where heat dissipation can affect thermal storage [145]); (iii) durability and standardization, as there is still a lack of normative methods to assess the long-term behavior of these materials. This reinforces the need for further studies on encapsulation and integration strategies to ensure the long-term stability of the PCMs [143].

### 7.2. PCM in Wallboards

Wallboards and gypsum panels constitute a significant portion of building surfaces, making them effective mediums for thermal energy exchange. Due to their favorable heat transfer properties, these materials are well suited for the integration of PCMs. In a study by Voelker et al. [49], gypsum wallboards were modified through the incorporation of microencapsulated PCM with a melting temperature range of 25 °C to 28 °C. The results indicated a temperature reduction of approximately 3 °C, along with a heating power rate of 2 k/min. Similarly, Hawes et al. [135] utilized the direct incorporation technique to embed 25–30 wt.% of PCM into gypsum wallboards. Their results demonstrated a temperature decrease of 4 °C compared to conventional wallboards, while maintaining acceptable levels of flammability resistance and moisture performance. Furthermore, Schossig et al. [13] applied microencapsulation to integrate PCM into wallboards. Over a three-week monitoring period, it was observed that rooms equipped with PCM-enhanced wallboards exceeded 28 °C for only 5 h, whereas the convectional setup surpassed this threshold for over 50 h. This highlighted the material’s capacity to moderate indoor temperature fluctuations effectively, confirming efficient thermal regulation through PCM integration.

PCMs used in wall panels can significantly reduce energy consumption and carbon dioxide emissions in buildings. Their effectiveness depends on their thermophysical properties, such as melting temperature and transition range, which should be tailored to specific climates to optimize energy performance, as highlighted by the authors Jebaei et al. [146]. The study conducted by the authors found that the use of optimal PCMs could lead to energy savings of up to 18.7% and substantial CO_2_ reductions—up to 15% for electricity consumption and 38% for natural gas use.

Shilei et al. [147] conducted a study over three consecutive winter days in northeastern China, incorporating 26% PCM into gypsum wallboards. Their findings showed that the presence of PCM helped to maintain indoor temperatures above outdoor levels, enhancing thermal comfort. In a separate investigation, Kuznik et al. [148] integrated 60% microencapsulated paraffin PCM into wallboards to assess its thermal performance. The study revealed that a 5 mm-thick PCM layer could store up to three times more energy than conventional wallboards, while also offering a favorable heat transfer coefficient through convection. Furthermore, Zhu et al. [149] proposed a composite double-layer wallboard with shape-stabilized phase change humidity control material (PCHCM) for building applications. Based on a numerical analysis conducted in an office building in Wuhan (China), the effects of the PCHCM on both building energy consumption and the indoor hygrothermal environment were studied. To minimize thermal loads, the optimal melting temperature of the PCHCM wallboards was found to be 25 °C for summer and 17 °C for winter, respectively, achieved by using two layers with different PCMs to optimize the melting temperature of the wallboards in various climate regions. The main conclusions were that the energy savings rate in the PCHCM building was 8.3% in summer and 24.9% in winter, compared to the reference building. The PCM control function was effective during the summer, but the HCM control function was not, though both functions proved useful in winter. During summer, the indoor air temperature in the PCHCM building was nearly the same as in other building types with air conditioners during the day. However, the indoor relative humidity in the PCHCM building was reduced by about 5%, compared to the reference building. In winter, the indoor air temperature was also nearly the same as in other building types during the day, while the indoor humidity was reduced by about 5%, compared to the reference building.

Also, the authors Li et al. [150] studied the inclusion of PCM in construction materials for indoor thermal comfort and energy savings. To adapt to seasonal changes like hot summers and cold winters, a novel hybrid PCM-containing wallboard was proposed (with three different PCMs), featuring a wide phase change temperature range to perform well in different climates. For this purpose, a building equipped with the hybrid PCM wallboard was evaluated to assess its energy-saving capabilities using Energy Plus^®^. To compare different hybrid PCM wallboards, four building models were considered, including Modes 1 through 3, and a gypsum wallboard (BASE). The building’s performance in terms of heating and cooling energy consumption over one year was examined at seven different locations in China, representing various climate regions. The results showed that Mode 2 required less heating and cooling energy than the other models. Further study of the Mode 2 PCM wallboard indicated that the location of the PCM layer also influenced energy savings. The total energy requirements for Mode 2 were lower than those for BASE by around 2.2%, 4.3%, 1.8%, 5.6%, 7.7%, 5.8%, and 0.2% for Shanghai, Beijing, Kunming, Lanzhou, Xining, Harbin, and Hong Kong, respectively. It was concluded that the hybrid PCM wallboard could effectively reduce heat gain through the building envelope throughout the year, offering considerable benefits for buildings located in Shanghai, which experience hot summers and cold winters.

Although gypsum and PCM wallboards offer substantial benefits, their effectiveness depends on proper selection and integration, highlighting the need for customized solutions based on specific construction requirements and environmental conditions.

### 7.3. PCM in Bricks

The integration of PCMs into bricks significantly improves their thermal performance and energy efficiency. PCMs, such as paraffin and calcium chloride hexahydrate, store and release thermal energy, promoting temperature regulation and stabilizing indoor temperatures. This results in reduced energy demand and contributes to energy consumption reduction. This innovative approach not only enhances thermal comfort but also supports sustainability in building design. For example, PCM-infused bricks can absorb excess heat during the day and release it at night, leading to a reduction in temperature fluctuations [151]. Moreover, as observed by [25,152] the thermal storage of these bricks results in a 2 h delay in thermal response compared to that of bricks without PCM, promoting energy efficiency in building materials.

Vicente and Silva [27] conducted an experimental study comparing three wall configurations (30 × 20 × 15 cm^3^): one built with standard bricks, another incorporating PCMs, and a third combining PCMs with an insulation layer. Their findings revealed that the combination of insulation and PCMs helped to reduce thermal peaks by 50% and 80%, respectively, while also delaying the peak by approximately three hours. In a related experiment, Silva et al. [28] evaluated two brick wall setups—one with PCM and one without. The results demonstrated a decrease in peak indoor temperature ranging from 5 °C to 10 °C due to the integration of PCM. Additionally, Gobinath et al. [35] embedded PCM within wall systems and observed a heat load reduction between 10% and 30% when compared to traditional plain walls. The study also noted room temperature reductions of approximately 2 to 4 °C, concluding that modular building units containing PCM provided greater energy efficiency than standard modular constructions.

Furthermore, Tunçbilek et al. [153] investigated the use of a nano-enhanced PCM to improve thermal conductivity. Their findings indicated that incorporating 1% by volume of alumina into a 3 cm-thick PCM layer resulted in a 0.6% reduction in heating energy savings compared to the use of pure PCMs. This negative effect became more pronounced with higher nanoparticle concentrations, reaching a 1.7% reduction in heating energy savings at 3 vol% of alumina. These findings indicate that, despite the improvement in thermal conductivity, the decrease in thermal resistance and latent heat capacity outweighs the benefits, making nanoparticle-enhanced PCMs less effective for building wall applications in the hot-summer Mediterranean climate.

Zhang et al. [154] found that incorporating PCMs into hollow brick walls led to an increase in temperature attenuation ranging from 1.5 °C to 5.0 °C, a rise in the damping factor from 1.0 to 2.5, and an extension in thermal lag by 0.5 to 3.0 h when compared to those of conventional brick walls. In a related study, Zhu et al. [155] analyzed the internal surface performance of such walls. Their results indicated that the attenuation ratio of the internal surface temperature amplitude in PCM brick wall was 3.8 to 4.4 times greater than that of traditional brick walls, while the delay ratio was 8 to 12 times higher. Additionally, the heat flux through the inner surface of conventional brick walls was found to be 3.0 to 3.9 times higher than that observed in PCM-integrated walls. The authors also noted that optimal unsteady-state thermal performance was achieved when the PCM operated within its designated phase change temperature range. Conversely, an increase in the boundary temperature range diminished the effectiveness of the PCM, leading to reduced thermal attenuation and time lag.

Mahdaoui et al. [26] examined the thermal behavior of bricks containing PCMs, focusing on key factors such as latent heat and melting temperature. Their study revealed that these two parameters had the most significant influence on performance. An increase in the PCM content within the bricks notably enhanced the thermal efficiency of the walls. Moreover, while the outer surface’s convective heat transfer coefficient had minimal influence, the inner surface coefficient played a more critical role. Positioning the PCM layer closer to the indoor side yielded results comparable to using dual PCM layers. However, selecting an inappropriate melting temperature could actually raise energy consumption during the summer months. For the Marmara region in Turkey, the ideal melting temperature was identified as 18 °C, which led to an annual energy consumption reduction of 17.6%.

Furthermore, Abbas et al. [156] proposed an innovative plug-and-play wall system, tested using two identical test rooms constructed with PCM bricks in Diwaniya city, under natural environmental conditions. The experimental findings demonstrated that, in comparison to a traditional wall, the PCM brick wall achieved a reduction of approximately 4.7 °C in the inner surface temperature. Additionally, the thermal lag was extended by 2 h, temperature fluctuations were diminished by about 23.8%, and the damping factor reached a value of 70%.

Although the benefits of PCM integration are substantial, challenges such as the initial material cost and the need for precise installation techniques may hinder widespread adoption. However, the potential for energy savings and enhanced thermal comfort makes PCM-enhanced bricks a promising solution for modern construction.

## 8. Performance Parameters for PCMs in Buildings

The thermal behavior of phase change materials (PCMs) in building applications is strongly affected by several key parameters. A comprehensive understanding of these factors is crucial for enhancing the thermal storage capacity and overall energy efficiency of PCMs in the context of building thermal regulation. The most critical influencing parameters are examined in the subsequent sections.

### 8.1. Melting Temperature of PCMs

The melting temperature of PCMs is a critical parameter, as it defines the point at which the material transitions from a solid to a liquid state. This directly influences the charging and discharging cycles that govern PCMs’ capacity for thermal energy storage. For effective performance, the melting temperature must be carefully to suit seasonal variations: it should be low enough to take advantage of limited solar radiation during winter for enhanced heating yet high enough to mitigate excessive solar heat gain in summer, thereby reducing cooling demands. Ideally, the melting point of PCMs used in buildings should be aligned with the indoor thermal comfort range—typically between 20 °C and 26 °C in residential environments. This ensures that PCMs can effectively absorb and release heat in response to daily temperature fluctuations, contributing to passive thermal regulation. The operational temperature range of a PCM must correspond to both the building’s internal thermal conditions and its intended energy efficiency targets. Since the indoor setpoint temperature—usually between 20 °C and 24 °C—varies depending on building function and climate, PCM selection must take into account the specific thermal comfort requirements of the space [157].

Additionally, optimal thermal performance requires that the melting point is matched to both the regional climate and seasonal dynamics. Different building types—residential, commercial, or industrial—may have distinct temperature requirements, influencing the appropriate choice of PCM. As noted by Jelle and Kalnaes [158], the recommended melting temperature varies by application: between 29 °C and 60 °C for water heating, 22 °C to 28 °C for indoor thermal comfort, and up to 21 °C for cooling-focused systems.

The melting point is decided by a few factors including the chemical structure, purity, and phase changing process. For example, organic PCMs such as paraffin waxes have relatively lower melting points compared to inorganic salts based on differences in intermolecular forces. Moreover, impurities in the material may lead to variations in melting point by disorganizing the crystalline structure, thereby producing a broader range of melting. Some PCMs also exhibit congruent melting where the material is fully melted uniformly, while some exhibit incongruent melting wherein a portion of the material is melted with possible phase segregation. The repeated cycling of thermal excitations, say, through solidification and melting cycle, could render the melting temperature to vary by material degradation or microstructural alterations [43].

The accurate measurement of melting temperature is important to identify PCMs. Differential scanning calorimetry (DSC) is the most versatile technique employed and provides detailed data on onset, peak, and end set melting temperatures. Thermogravimetric analysis (TGA) is another technique that can be employed to determine thermal stability and phase change properties of PCMs. The measurements are important to select the proper PCM for a given application. For example, in building energy efficiency, PCMs with melting temperatures close to the target indoor temperature can significantly reduce heating and cooling loads. High-melting-point PCMs in solar storage can store thermal energy during maximum sunlight and discharge stored energy when needed, which strongly enhanced the efficiency of the system [80].

These benefits are, nevertheless, accompanied by limitations like subcooling, phase separation, and low thermal conductivity, which restrict their performance. Subcooling is experienced when a PCM freezes at a temperature below its melting point, thereby reducing the effectiveness of heat release. Phase separation, especially among salt hydrates, can result in non-uniform properties over the duration of the test. To address these problems, scientists are designing composite PCMs and incorporating nanoparticles for increased thermal conductivity and stability. These developments seek to expand the scope of the available PCMs and enhance their performance in different applications [159].

### 8.2. Thickness of PCMs

The thickness of PCM layers plays a crucial role in defining both their thermal energy storage capacity and the rate at which heat is transferred. While increased thickness allows for greater energy storage, it can also hinder the speed of thermal response due to higher thermal resistance [160]. The material used to encapsulate or integrate PCMs significantly influences the optimal thickness, as materials with higher thermal conductivity enhance heat transfer efficiency. In practical applications, the physical constraints of building structures often limit the maximum allowable thickness of PCM layers. Therefore, selecting an appropriate thickness involves balancing available space with performance requirements. In addition to spatial limitations, economic factors must be considered; thicker PCM layers tend to be more expensive, necessitating an evaluation of cost–benefit trade-offs to ensure both energy performance and affordability. Moreover, the placement of PCM within the building envelope has a substantial impact on its effectiveness. Strategic positioning is essential for maximizing the regulation of indoor temperatures and achieving desired thermal comfort outcomes. Finally, it should be noted that the thickness of PCMs needs to be carefully examined and coupled with the impact of the melting point of the PCMs to ensure their beneficial thermal performance without affecting the mechanical strength of the building parts.

One of the most important considerations in determining the optimal thickness of PCMs is the compromise between heat transfer rates and thermal storage. Greater amounts of thermal energy can be stored in thicker PCM layers since there is more volume to undergo phase change. But, thicker layers also provide higher thermal resistance, which reduces heat transfer and decreases the rate of energy absorption or release [161]. This equilibrium is essential in applications such as building envelopes, where PCMs are integrated into walls or ceilings to reduce heating and cooling loads. Experiments have shown that there is an optimum thickness for each application beyond which the benefits of additional energy storage are countered by reduced heat transfer efficiency [162,163].

In thermal energy storage systems, the thickness of PCM affects charging and discharging cycles. Thicker PCM layers require longer times to melt completely or to solidify and may be appropriate for applications that require long-term thermal regulation, e.g., solar energy storage. However, for systems requiring fast thermal response, e.g., electronic cooling, thinner PCM layers are preferable to achieve rapid heat absorption and rejection. The thermal conductivity of PCM and the design of the containment system also play important roles in determining the optimum thickness. For instance, the incorporation of thermal conductivity enhancers, i.e., nanoparticles or metal foams, can overcome the limitations imposed by additional PCM layers. Furthermore, PCMs’ economic feasibility and optimal utilization are also determined by their thickness. Larger thickness requires more material, meaning higher costs and potentially more complicated installation, especially in applications with confined space. Therefore, achieving optimal thickness for maximum thermal performance with the minimum use of material is essential for the widespread application of PCMs. Experimental research and computational modeling are typically employed to determine the optimum thickness for an application, considering the thermal load, climatic conditions, and system design [164,165].

### 8.3. Positioning of PCMs

The positioning of PCMs within the building’s structure significantly influences their ability to regulate indoor temperatures effectively. The optimal location of PCM layers is determined by both the building’s geographic context and the intended function of the PCM—whether it is implemented primarily to reduce heating or cooling loads. Locating PCMs in building components exposed to high thermal loads, such as external walls and roofs, can enhance thermal regulation by enabling the material to absorb surplus heat during the day and release it during cooler periods at night. Numerous studies have indicated that placing a PCM layer closer to the primary heat source increases its effectiveness [166].

Some research suggests that embedding PCMs within the middle of structural elements may yield improved year-round thermal performance [167]. The desired thermal outcome—cooling or heating—also affects the ideal placement: for cooling purposes, PCM layers are generally more effective when positioned on the exterior side of building elements, while for heating applications, they should be located closer to the interior side [168]. In addition, the melting temperature of PCM is a decisive factor in determining its most effective position. For instance, Lagou et al. [169] conducted a study to identify the optimal location and melting temperature of PCMs across various European climates using COMSOL^®^ Multiphysics simulations. The analysis focused on unconditioned wall assemblies with different orientations, building types, and geographic locations. The findings revealed that the interior surface of the wall consistently provided the best performance, regardless of conditions. Moreover, the optimal melting temperature varied with climate zone—approximately 16 °C for central regions, 20 °C for southern areas, and 11 °C for northern European cities—ensuring optimal thermal efficiency in each context.

Moreover, the authors Darvishi et al. [170] numerically studied the best position of PCMs with melting points between 21 °C and 25 °C for two Iranian cities under distinct climatic conditions. The research team concluded that annually placing PCM in the middle or near the interior zone decreased the thermal load and increased the energy savings independently of the climate. In sum, PCM placement should ensure that the thermal regulation contributes to the comfort of the building occupants without causing undesirable thermal fluctuations. Optimizing the thermal performance of PCMs in buildings requires careful consideration of key parameters such as melting temperature, material quantity, and thickness, as well as their spatial positioning within the building envelope. By tailoring these factors to the specific climatic conditions, architectural characteristics, and functional requirements of the building, PCMs can play a vital role in improving both energy efficiency and indoor thermal comfort.

### 8.4. Heat Transfer Fluid

In active thermal management systems, heat is stored and released through mechanical components such as pumps, fans, and blowers, where the role of the heat transfer fluid (HTF) becomes critical. In hot climatic conditions, HTFs are essential for extracting the accumulated heat from PCM and facilitating its solidification for the subsequent thermal cycle. Nighttime, characterized by relatively lower ambient temperatures, is typically utilized for this purpose—particularly in controlled systems—where the cooler night air aids in solidifying the melted PCM [171]. Conventional night cooling system designs generally operate on a single day cycle: the PCM melts during daytime hours due to rising temperatures and re-solidifies at night via exposure to cooler air. However, the effectiveness of such systems is limited in regions with extreme heat, where diurnal temperature variations are minimal. For instance, the efficacy of night cooling declines significantly when the temperature difference between day and night falls below approximately 15 °C [172]. Studies have reported issues such as the incomplete solidification of PCM when relying solely on natural night cooling as the HTF [173]. In such scenarios, high daytime solar radiation and heat retained in building envelope materials can keep the surface temperature elevated throughout the night, preventing full re-solidification of the PCM. Consequently, in the subsequent cycle, the PCM may only partially melt, thereby reducing its thermal storage capacity.

To improve the efficiency of active night cooling, various operational parameters—such as the night air temperature range, extent of PCM solidification, airflow rate, and ventilation duration—must be carefully regulated [174]. In regions with harsh summer conditions, alternative HTFs may be necessary to ensure the complete solidification of the PCM. Passive strategies, including building orientation and shading techniques, have also been found to be effective in minimizing solar gains and supporting more efficient night cooling performance [175]. In cold climates, HTFs are equally important, particularly for enhancing the charging phase of PCMs. Due to limited solar radiation in such regions, passive heat gain is often insufficient to activate PCM. Therefore, active systems incorporating solar collectors—using water or air as HTFs—are required to effectively capture and store solar energy during daylight hours for subsequent thermal use.

HTFs such as water, oil, or molten salts pass through or surround the PCM, collecting heat on discharge and delivering it to where it is needed [176]. HTF choice is influenced by the working temperature range, thermal properties, and compatibility with the PCM and system parts. For instance, water is commonly utilized in low-temperature systems, while synthetic oils and molten salts are utilized in high-temperature systems, such as concentrated solar power plants [177].

The arrangement of the heat exchange system, in which the HTF and PCM are mixed, plays a crucial role in ensuring maximum heat transfer efficiency. In heat exchangers such as shell-and-tube heat exchangers, the HTF flows through tubes while the PCM is kept in the outer shell. This allows for a large surface area for heat exchange, which ensures efficient heat transfer during charging and discharging [157]. Alternatively, in encapsulated systems using PCM, the HTF flows around tiny capsules of the PCM, augmenting the heat transfer due to the enhanced surface area-to-volume ratio. CFD modeling and experimentation are widely utilized to achieve maximum design in such systems with an outlook to low thermal resistance and the highest possible recovery of energy [178].

Improvements in HTF technology have also improved the performance of PCM-based systems. For example, nanofluids, i.e., suspended nanoparticles in HTFs, have been shown to enhance thermal conductivity and heat transfer rates significantly [179]. Such better HTFs can potentially improve the performance of PCM systems, particularly for applications that require rapid heat release, e.g., electronic cooling or industrial process heating. Similarly, the use of phase change slurries, in which PCM particles are suspended in a carrier fluid, has been explored to integrate the heat transfer and energy storage capabilities into a single medium [45].

## 9. Applications for PCMs in Building Thermal Management

The potential of PCMs for thermal management in buildings makes them effective in regulating indoor temperatures, thereby enhancing thermal comfort. These materials help reduce reliance on active heating and cooling systems, which in turn lowers energy consumption. The integration of PCMs into building components can occur in ceilings, walls, floors, among other elements. For example, studies have shown that incorporating PCMs into ceilings significantly reduces indoor air temperature, improving thermal comfort without the need for active cooling systems [180].

PCMs can significantly enhance the thermal inertia of lightweight building structures by increasing their capacity to storage and regulate heat. For optimal energy savings, the selection of an appropriate melting point is essential: in cooling-dominated climates, PCMs with melting temperatures around 26 °C are recommended, whereas heating-dominated climates benefit more from PCMs with melting points closer to 20 °C [181]. One of the major limitations of conventional PCMs is their inherently low thermal conductivity, which can restrict the rate of heat exchange. A promising approach to overcome this drawback involves the incorporation of highly conductive nanostructured additives—commonly referred to as nano-enhanced PCMs. These nanofillers, which may be categorized according to their dimensional structure (0D, 1D, 2D, 3D, or hybrid), significantly improve thermal performance. Among these, carbon-based nanomaterials have demonstrated superior enhancement of thermal conductivity when compared to metal or metal oxide nanoparticles. For example, graphene exhibits an exceptionally high thermal conductivity, often exceeding 2000 W·m^−1^·K^−1^. Depending on their morphology, these nanomaterials—such as nanoparticles, nanofibers, or nanotubes—not only modify the base PCM’s thermal conductivity but also influence other thermophysical properties, including subcooling behavior, phase change temperature and duration, density, and viscosity.

As an example of an evaluation study on the applicability of nano-enhanced PCMs for thermal management of buildings, George et al. [182] conducted a comparative analysis by dispersing both polyaniline and copper oxide in paraffin. The increase in thermal conductivity was approximately 46.7% and 63.5% for polyaniline and copper oxide and the latent heat was also augmented by nearly 8.3% and 7.8% for the polyaniline and copper oxide nanostructures, respectively. Moreover, the researchers Habib et al. [183] obtained an increase of around 61% in the thermal conductivity with single-walled carbon nanotubes added to paraffin, and the authors Sheng et al. [184] used carbon fibers as nanofillers, and the thermal conductivity was drastically increased by nearly 150%. Some researchers have highlighted the pressing need for further works on the synthesis and thermophysical characterization of low-temperature organic nano-enhanced PCMs used in ambient conditions.

**Figure 9:**
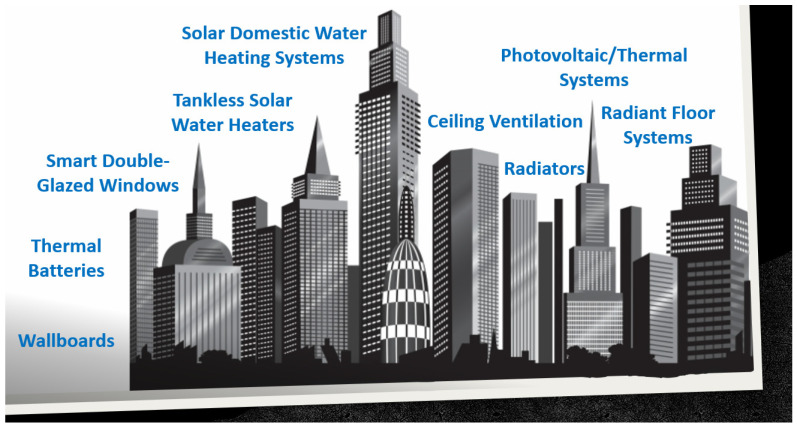
The main technological solutions using nano-enhanced PCMs for the thermal management of buildings.

Figure 9 presents the main technological solutions using nano-enhanced PCMs to be applied in residential buildings’ thermal management.

## 10. Energy Performance and Efficiency in Residential Buildings

**Figure 10:**
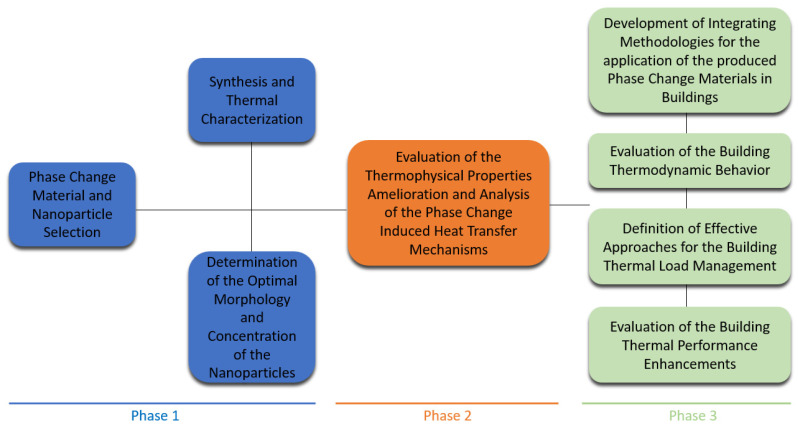
The main phases of the methodology of development and implementation of nano-enhanced PCMs in buildings.

Nano-enhanced PCMs can be used in building structures, both in lightweight and heavyweight buildings, to increase the overall and local thermal mass. They can also be incorporated into heating, ventilation, and air-conditioning (HVAC) systems as energy storage solutions, reducing the mismatch between energy supply and demand by substantially shifting and reducing peak loads. Investigating the thermal performance enhancement of residential buildings using nano-enhanced PCMs is challenging due to the many impacting factors and considerable uncertainties associated with the physics of residential buildings. Furthermore, Figure 10 illustrates a framework for the proper in-lab development and local implementation of nano-enhanced PCMs in building applications. This framework includes material selection; synthesis and thermal characterization; the examination of thermal conductivity enhancement; the underlying mechanisms of phase change energy transport; and the investigation of building thermodynamic behavior and thermal performance improvement when using nano-enhanced PCM.

In the United Kingdom, PCMs integrated into the internal walls of residential buildings, such as detached houses, showed significant reductions of up to 30% in the heat loss in comparison to other types of housing, such as apartments and terraced houses, which had reductions of 8–14% [16].

In the study conducted by Zhang et al. [185], the authors evaluated the energy performance and efficiency of residential buildings using an integrated latent thermal energy storage (ILHTES) system with a PCM-to-air heat exchanger (PAHX). Among the four commercially available PCMs examined—RT27, RT25, RT20, and RT18—RT25 demonstrated superior performance. The researchers concluded that the choice of PCM significantly influenced the Energy Saving Ratio (ESR), with RT25 showing the best performance. In five European cities, the optimized ILHTES system achieved an ESR ranging from 16% in Catania to 44.7% in Stockholm, highlighting the potential for substantial energy savings compared to conventional air conditioning systems.

Another study, performed in [36], evaluated the energy performance in residential buildings using PCMs applied to the building envelope, internal walls, and internal floors. The study was conducted in two locations: Istanbul, representing a temperate humid climate zone, and Diyarbakir, representing a hot–dry climate zone. In Istanbul, the total energy load was reduced by almost 12%, while in Diyarbakir, it decreased by nearly 9.7%. The application of PCMs improved the indoor comfort conditions, with operative temperature variations ranging from 1.04 °C to 3.32 °C in Istanbul and from 0.87 °C to 1.16 °C in Diyarbakir. These results demonstrated higher energy efficiency under different climatic conditions, contributing to the improvement of thermal comfort in the indoor spaces of the buildings. The evaluation was conducted using the Energy Plus^®^ simulation tool, and the calculations were made for the A4.2 alternative, where the PCM was applied to both the building envelope and internal walls and floors, aiming to reduce the energy consumption differences and thermal conditions between the different zones of the buildings.

**Figure 11:**
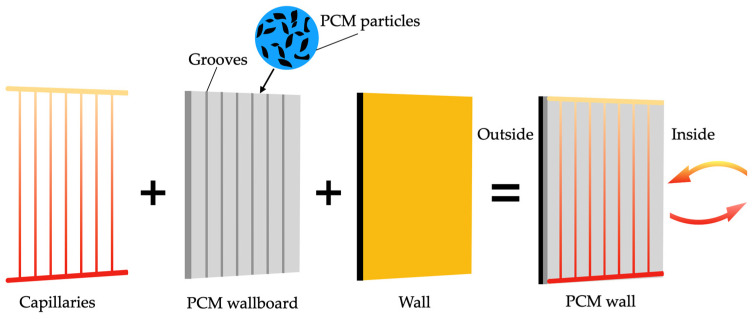
An example of the integration of PCMs in a door of a residential building. Adapted from [186].

Moreover, Kong et al. [186] conducted an experimental investigation into a hybrid thermal management system that integrated an active composite PCM wall with a solar thermal heating unit for wintertime building applications. In their study, the researchers developed an innovative composite PCM wallboards based on expanded perlite, which were subsequently coupled with the solar thermal system via embedded capillary tubes to facilitate heat transfer and enhance system efficiency. Comparative experiments were performed in two real-scale experimental rooms (a PCM room and a reference room). PCM wallboards coupled with the solar thermal system were installed on the internal surface of the PCM room, while the reference room was identical to the PCM room, except that it did not include the PCM wallboards. The results showed that the PCM wallboards, which exhibited improved mechanical strength and thermal properties, performed well when integrated with the solar thermal system. This hybrid system reduced daily energy consumption by approximately 44.2%. Figure 11 schematically illustrates the structure of the developed wallboards.

## 11. Advances in PCM Integration in Buildings for Enhancing Thermal Comfort

**Figure 12:**
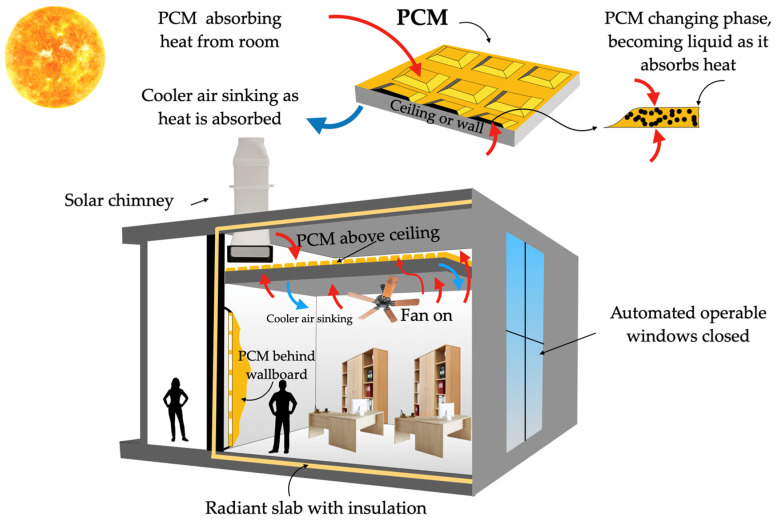
PCMs applied in buildings to enhance thermal comfort.

Figure 12 shows the integration of PCMs in a building to improve indoor thermal comfort. In this example, the PCMs store and release heat during phase transition, contributing to the regulation of the indoor temperature of the building. As has been discussed throughout this work, PCMs can be applied in different building parts such as walls, ceilings, floors, and windows or even, in certain cases, in mobile elements like, for instance, curtains.

### 11.1. Roofs

PCMs, when integrated with a building’s roof, can enhance heat storage efficiency. Several parameters directly influence the thermal performance of a roof containing a PCM, including the quality of the PCM, the thickness and location of the roof, and climatic conditions. In this context, Bhamare et al. [187] introduced a novel evaluation metric—referred to as the Measure of Key Response (MKR) index—to support the selection of PCMs and to assess the influence of their thermophysical properties on the thermal performance of PCM-integrated roofs in various climatic zones across India, specifically in Delhi, Ahmadabad, and Kolkata. The metric incorporates parameters such as time lag, ceiling temperature, heat gains, and the decrement factor, all of which are affected in the integration of PCMs into roofing systems. The study employed both experimental approaches and numerical simulations to evaluate PCM performance under distinct climatic regions. The Response Surface Methodology (RSM) was used to design and analyze the experiments, allowing the identification of the most suitable PCM based on the maximization of the MKR index. The results demonstrated that the PCM properties exerted a climate-specific influence on thermal performance, underscoring the necessity of customized PCM selection for each region. Among the various thermophysical parameters analyzed, thermal conductivity and melting temperature were identified as the most significant factors impacting the MKR index, surpassing others such as density, latent heat, and specific heat. In the composite climate zone (Delhi), thermal conductivity was found to be the dominant parameter. Conversely, in the hot climate of Ahmedabad and the warm–humid climate of Kolkata, melting temperature emerged as the most critical property, followed by thermal conductivity. The optimal MKR index was achieved with the highest thermal conductivity and the lowest melting temperature. In summary, enhancing the thermal performance of a PCM-integrated roof requires careful selection of PCM properties. PCMs with a melting temperature of 28 °C and a thermal conductivity of 0.39 W/m·K were identified as the optimal choice.

Luo et al. [188] conducted a study using climatic data from a typical June in Shanghai to evaluate the heat transfer performance of a porous brick roof system incorporating PCM. The PCM used was paraffin wax with multiple phase transition temperatures. The researchers employed the Multiple-Relaxation-Time Lattice Boltzmann Method (MRT-LBM), optimized through GPU acceleration to leverage its high flexibility and parallel computational efficiency. Their findings showed that at transition temperatures of 25 °C and 26 °C, the PCM underwent complete melting throughout the day, whit a lower melting points resulting in a higher proportion of the liquid phase. A notable midday temperature difference was observed between roofs filled with PCM and those containing only air, demonstrating the significant thermal buffering capacity of the PCM system. Specifically, at a phase change temperature of 27 °C, the PCM-filled roof reduced heat flow to 67.8 kJ/m^2^, compared to 73.4 kJ/m^2^ in the air-filled counterpart. Furthermore, the air-filled porous brick roof exhibited the highest temperature fluctuation, reaching up to 8.6 °C, whereas the inclusion of PCMs considerably improved thermal inertia and contributed to a decrease in energy demand for air-conditioning systems. Additionally, the study explored the integration of thermo-chromic coatings and PCM layers into building roofs to dynamically modulate solar radiation absorption and heat transfer. Simulations demonstrated that roofs utilizing thermo-chromic materials, PCMs, and a combined thermo-chromic–PCM approach achieved energy consumption reductions of 13%, 15%, and 17%, respectively, when compared to conventional asphalt roofs. The impact of increasing the thermo-chromic coating thickness from 10 mm to 50 mm was also assessed, showing cooling load reductions of 7% (5.1 kWh/m^2^) in Beijing, 8% (3.0 kWh/m^2^) in Heilongjiang, 5% (5.2 kWh/m^2^) in Nanjing, 5% (7.0 kWh/m^2^) in Guangdong, and 10% (5.1 kWh/m^2^) in Kunming. These findings underscore the potential of TC–PCM hybrid roofing systems to provide seasonally adaptive thermal management and enhance overall building energy efficiency.

Triano-Juárez et al. [189] studied heat transmission through concrete roofs by incorporating an intermediate PCM layer in a Mexican city with a warm and humid climate. Different thicknesses, placements of the PCM layers, and roof solar absorptance values were analyzed. The authors observed that placing the PCM layer on the internal surface of the roof reduced the temperature by 6.4 °C and heat gains by approximately 22%. In the case of white reflective roofs, only slight differences were observed in the peak interior surface temperature and the associated heat gains when varying the thickness and placement of the PCM layer. However, as the PCM layer was positioned closer to the interior surface and its thickness increased, notable changes in its phase change cycles were identified. The integration of a white reflective coating was particularly effective, leading to a reduction in the peak interior surface temperature by approximately 14.7 to 15.4 °C and achieving a substantial decrease in cooling load, ranging from 58.1% to 62.7%.

**Figure 13:**
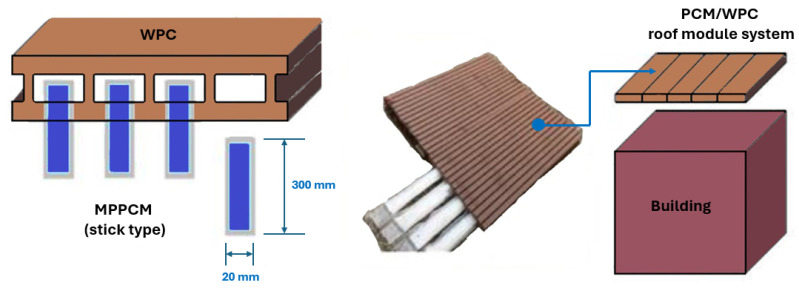
PCM/wood–plastic composite roof developed by Chang et al. [190]. Adapted from [190].

Chang et al. [190] proposed a PCM/wood–plastic composite roof to lower the temperature of a conventional roof and improve indoor thermal comfort. The proposed system reduced surface temperatures by approximately 7.4 °C. Additionally, an increase in PCM thickness was found to correlate with a greater reduction in surface temperature. Among the tested materials, PCMs with a phase transition temperature of 30 °C demonstrated the most pronounced decrease in surface temperature. However, PCMs with a transition temperature of 20 °C proved to be more effective in improving both thermal comfort and overall energy efficiency. The proposed composite roof configuration is illustrated in Figure 13.

Elawady et al. [191] investigated the thermal performance of PCM-coated roofs by evaluating various PCM types and layer thicknesses. To simulate the phase change process, both the enthalpy–porosity method and a simplified thermal model were utilized, with the latter deemed more suitable for long-term simulations. The study focused on the summer climate of Aswan, Egypt, assessing the impact of PCM integration on reducing internal heat flux and maintaining wall surface temperature within comfort thresholds. A four-month simulation revealed that a conventional roof led to an average side-wall temperature of 32.5 °C, whereas a roof incorporating PCM achieved a lower average of 29.4 °C. Specifically, the inclusion of a 40 mm RT31-PCM layer resulted in a 40% reduction in energy gain. Furthermore, the presence of natural ventilation extended the PCM solidification period by 19–41%. The overall cooling load was reduced by approximately 6.9%, while thermal comfort duration improved by an additional 50 min. The most favorable outcome was obtained when the PCM layer was coupled with an air gap and used without natural ventilation, yielding a peak indoor air temperature reduction of 2.5 °C.

Rangel et al. [192] examined the synergistic effects of PCMs and natural ventilation on the thermal performance of roof in semi-arid climates. The study evaluated various configurations, including a continuous roof structure (Case A) and systems incorporating air gaps (Case B), both with and without the presence of natural ventilation. Key parameters analyzed included surface temperature, heat flux, time lag to peak temperature, and indoor air temperature. The findings showed that the PCM systems produced a peak time lag ranging from −10 to 70 min and enabled a reduction in internal air temperature of approximately 3.94% to 7.02%. Natural ventilation was found to increase the PCM solidification duration by 19–41%, while the maximum reduction in cooling load was recorded at 6.85%. Additionally, thermal comfort was extended by 50 min. The configuration yielding the best thermal performance was combined with a 30 cm air gap, in the absence of natural ventilation. Another approach involved using PCM foamed cement roofs (FCRs) to enhance thermal performance. The study evaluated two scenarios: one with a high-reflectivity film applied to the external surface of the roof (Case 1) and another without the film (Case 2). The results showed that the PCM FCRs lowered the interior surface temperature by 2.0 °C in Case 1 and 2.9 °C in Case 2, whereas traditional FCRs reduced temperatures by 1.1 °C and 2.5 °C, respectively. Regarding heat gain reduction, the PCM FCRs and traditional FCRs achieved reductions of 48.5% and 19.4% in Scenario 1, while Scenario 2 resulted in average reductions of 59.0% and 51.2%, respectively. Among the three roof types analyzed, the PCM FCRs demonstrated the most significant impact in reducing interior surface temperature and heat gain.

Sedaghat et al. [193] carried out experimental and numerical investigations on bio-based PCMs and reflective roof coatings as energy-saving strategies in three hot-climate regions. Two identical portable cabins were meticulously constructed and tested under two cooling scenarios. Since their thermal similarity was experimentally validated, these rooms provided an ideal environment for evaluating energy-saving approaches. A six-zone model was developed in TRNSYS to simulate the performance of bio-PCM-integrated walls. The study demonstrated that incorporation of bio-PCMs in three different wall and roof positions led to energy savings of 22% in Kuwait, 23% in Australia, and 53% in India. Furthermore, simulations revealed that sandwich panels could reduce energy consumption by 35.7% in Kuwait and 43.8% in Australia when compared to autoclaved aerated concrete blocks. However, in Kodaikanal (India), the use of sandwich panels resulted in a notable increase in energy consumption, reaching 126.2%.

### 11.2. Ceilings

Basher et al. [194] investigated a PCM as a thermal insulation solution for building walls and ceilings. An energy and weather simulation software supplied climatic data for Kut City (Iraq) during their analysis. The ESP-r software modeled two identical rooms: a reference room for baseline comparison and a test room for experimentation. The study assessed different PCM thicknesses and installation locations (ceiling, west wall, east wall, south wall, and north wall). The results indicated that PCM insulation effectively reduced indoor temperature, cooling demand, and overall energy consumption. The findings emphasized the role of PCM thickness in thermal regulation; thicker layers offered enhanced insulation, ensuring lower internal temperatures. Additionally, the orientation of the PCM significantly influenced performance, with the south-facing wall showing the most notable temperature drop due to direct solar exposure. Ultimately, the PCM proved effective in decreasing cooling loads, contributing to potential energy savings.

Yasin et al. [29] developed a TRNSYS-based room model for a chilled ceiling system integrating PCMs. The PCM–chilled ceiling configuration employed TRNSYS Type 399, which was designed to simulate large thermo-active building components embedded with PCMs. The root mean square errors were ±0.3 °C and ±0.6 °C for the simulated and validated deviations in operational and PCM temperatures, respectively. The simulation of the ceiling’s cooling capacity with PCMs showed a higher variation, with a root mean square error of ±0.19 kW. Despite this, the model can be used to optimize the design and operation of such systems, improving thermal comfort and energy performance.

Abden et al. [195] created a composite form-stable phase change material (FSPCM) by directly impregnating gypsum board with methyl stearate and diatomite. Thermogravimetric analysis and thermal cycle tests demonstrated that the FCPCM was dependable and robust for long-term temperature regulation. To evaluate its performance, an energy-efficient test ceiling was created by integrating the FSPCM into a gypsum board. A small test room was modeled with a ceiling composed of gypsum board embedded with FSPCM and its thermal and energy efficiency, as well as cost effectiveness, were compared against conventional gypsum boards without FSPCM under real climatic conditions. The results showed that the incorporation of FSPCM in gypsum board ceilings resulted in a 16.2% decrease in cooling demand.

Velasco-Carrasco et al. [196] analyzed the thermal performance of buildings featuring S23 ceiling panels. The test room was artificially heated to establish the melting point of the PCM panels. The S23 panels absorbed heat, increasing the ambient temperature by 5 °C. Furthermore, PCM ceiling tiles helped maintain favorable indoor temperatures during cooling periods. After six hours of cooling, the temperature of the S23 panels fell below their melting point, completing the thermal cycle. Additionally, the S23 panels effectively encapsulated PCM and improved heat transfer within the thermal environment, making them a viable option for building applications. The results also demonstrated that the thermal conductivity of the S23 panels varied from 0.19 to 0.24 W/m·K, offering a superior alternative compared to other commercially available PCMs. The room temperature of the PCM dropped to 14.6 °C during the initial six hours, later stabilizing at 22.1 °C, which remained below the PCM’s melting point.

Additionally, Bogatu et al. [197] assessed a macroencapsulated PCM panel with integrated piping, comparing its efficacy to commercial radiant cooling systems as an efficient ceiling cooling solution. Owing to the built-in heat-storage capacity, the cooling load could be shifted to off-peak hours when the PCM was fully discharged. At 20 °C and a mass flow rate of 140 kg·h^−1^, the specific cooling power of the macroencapsulated PCM panel (MEP) on the active ceiling surface was 11.3 W·m^−2^, with values spanning from 5.3 to 27.7 W·m^−2^. The findings indicated that the overall heat capacity of the PCM within a single MEP, for temperatures between 16 and 31 °C, was approximately 361 Wh·m^−2^, with 242 Wh·m^−2^ falling within the melting range. For 83% of the occupied duration, the MEP’s performance was comparable to that of radiant ceiling panels, delivering a thermal environment categorized as Category II under the EN-16798 standard. In comparison to effectively cooled gypsum panels—where MEP pipes were in direct contact but not embedded—this configuration represented a notable improvement.

Skovajsa et al. [198] explored the potential of PCMs in cooling-integrated ceilings to decrease energy use and minimize temperature fluctuations. The authors developed a PCM-based cooling ceiling and carried out an experimental validation of TRNSYS-based transient simulations. Based on the findings, comprehensive simulations were conducted under the climatic conditions of Czechia. The suggested cooling ceiling system lowered temperature peaks by 3.2 °C and achieved energy savings of 27%, depending on the air exchange rate.

Moreover, Mousavi et al. [199] examined a PCM-integrated Radiant Chilled Ceiling (PCM-RCC) in a test cabin situated in Melbourne. To assess the thermal and energy efficiency of the system, the authors examined the transient thermal conduction of the PCM panels during charging and discharging cycles, along with electrical peak demand, indoor thermal comfort, and other performance indicators. The proposed PCM-RCC system can provide adequate comfort and load shifting when operated under a well-designed strategy. The design incorporates multiple variables to maximize the overnight recharge efficiency of the PCM. If the system does not overheat before midnight, 6–8 h should be sufficient for recharging. Nevertheless, the existing panel configuration encounters difficulties due to the limited heat transfer efficiency of the water capillary tubes and the insufficient thermal interface between the PCM and these tubes. The PCM-RCC system maintains interior temperatures within the Class C range defined by the ISO 7730 standard [200] for 58 to 70% of occupancy duration. Therefore, this research emphasized that the adoption of operational and control mechanisms is essential to fully exploit the capabilities of PCM-RCC solutions.

Mustafa et al. [201] improved a radiant chilled ceiling system by embedding PCMs and carried out an in-depth thermal evaluation in Tabuk (Saudi Arabia). The PCM was embedded in the radiant chilled ceiling by incorporating air gaps. Consequently, the annual heat exchange from the ceiling was lowered by 57.6%, and by 22.6% during the April-to-October period, assuming adequate PCM placement and air gap configuration. For uninterrupted operation throughout the day, the PCM had to re-solidify during the night. Moreover, the cool external wind assisted in cooling the water, while a nanofluid-based heat exchanger aided in PCM freezing. The initial strategy accelerated PCM solidification by 78.6%, whereas the second and third strategies enhanced the solidification rate by 16.5%. When chilled airflow was introduced into the internal HVAC system, electricity usage dropped by 26.1% in April and 25.3% in October; however, no notable reductions were found during July and August.

Khattari et al. [202] assessed the potential benefits of using paraffin C13 PCM in a cooling ceiling system under Moroccan climate conditions, representing three Köppen–Geiger climate classifications. The controlled cooling power maintained indoor air temperature within a narrow thermal comfort range without excessive energy waste. By incorporating real ambient temperature data from Marrakech (BSh climate), Fez (Csa climate), and Ifrane (Csb climate), a comprehensive 2D transient simulation was conducted using computational fluid dynamics to solve the governing physical equations. The paraffin C13 PCM effectively reduced indoor air temperature fluctuations in all three climate types and achieved cooling power savings of approximately 17.1% and 16.3% in the Csa and Csb climates, respectively.

Yang et al. [203] investigated conjugate heat transport in a system comprising oleic acid, capric acid, and lauric acid as PCMs, along with the panel shell and surrounding air. The PCM was encapsulated in acrylonitrile–butadiene–styrene (ABS) plastic thin-shell enclosures, designed for esthetic ceiling panels. Using full-scale experimental data, a computational fluid dynamics (CFD) model was developed to analyze the discharge behavior and optimize the design of PCM ceiling panels. Results indicated that pyramid-array panels exhibited superior thermal performance compared to tetrahedral panels, with an average melting rate 20.8% higher at a PCM volume of 250 mL. Additionally, the active heat transfer area and natural convection conditions within the panel significantly influenced its thermal performance, particularly when modifying its geometric parameters. Finally, suspending the panel from the ceiling enhanced heat transfer more effectively than direct attachment.

Arivazhagan et al. [180] conducted a comparative analysis of two building models to assess the impact of PCM in ceilings for thermal management. In their work, the organic PCM OM-30, with a melting point of 31.1 °C, was encapsulated in high-density polyethylene. The investigation examined PCM melting behavior in different temperature conditions—above, within, near, and distant from the PCM layer. The results showed that the presence of PCM led to reductions in indoor air temperature of approximately 1.7 °C, 5.8 °C, 2.3 °C, and −2.9 °C, corresponding to PCM temperature differentials of around 8.8 °C, 1.6 °C, 1 °C, and 0.4 °C under free-floating ambient conditions. Furthermore, compared to a conventional reference room, the PCM-enhanced room utilized approximately 3.2%, 31.4%, 6.9%, and 12.3% of the latent heat energy to achieve the respective temperature variations of 1.7, 5.8, 2.3, and −2.9 °C.

**Figure 14:**
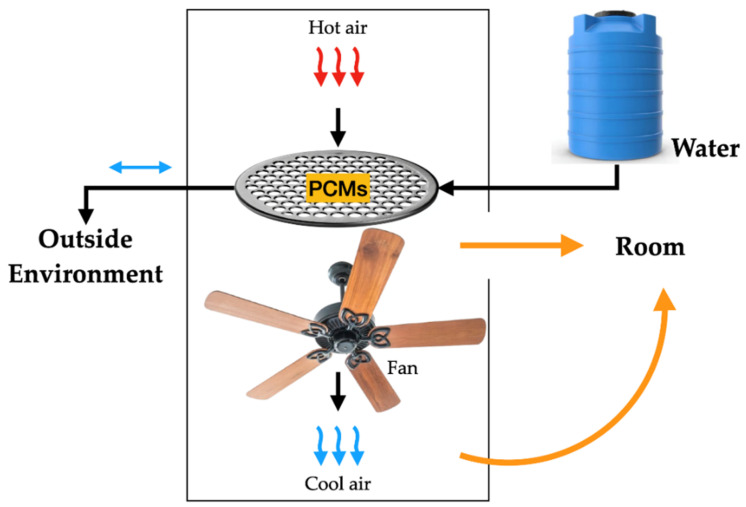
Ceiling fan configuration incorporating PCM for cooling. Adapted from Stalin et al. [204].

Stalin et al. [204] designed a ceiling fan integrated with a PCM system. A spherical PCM disk made of paraffin wax, positioned 30 cm above the fan, is illustrated in the schematic shown in Figure 14. The PCM disk incorporates small aluminum tubes that enable the flow of water from the building’s tank to an outlet directed toward the environment. This study serves as a reference for validating the outcomes of a numerical simulation model for PCM-based cold storage in buildings. The modified ceiling fan demonstrated effective cooling performance.

**Figure 15:**
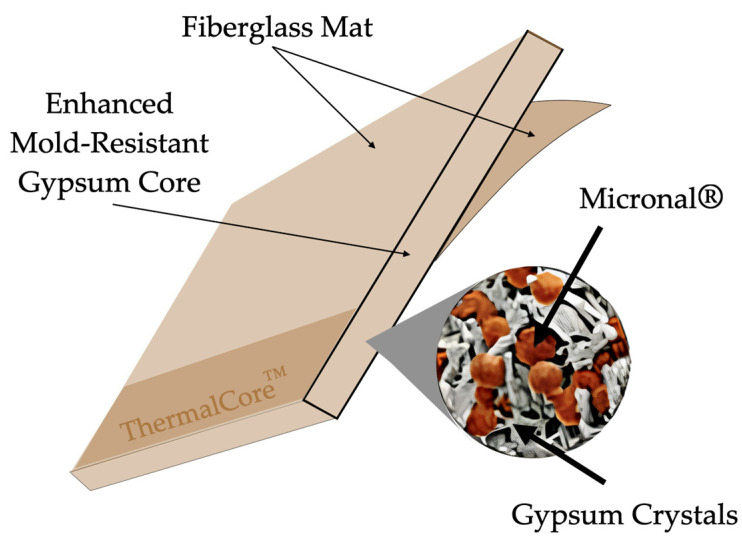
Phase change drywall with ThermalCore from National Gypsum.

A gypsum board company, National Gypsum (National Gypsum Company®, Charlotte, NC, USA), produced wall panels with Micronal PCM. These panels are known as National Gypsum ThermalCore Panels and have a latent capacity of 22 BTU/ft^2^ and a melting point of 23 °C. A schematic of their design is shown in Figure 15.

Figure 14 and Figure 15 illustrate examples of efficient solutions for maintaining indoor thermal comfort in residential, commercial, and other building environments. The ceiling fan with an integrated PCM system (Figure 14) and the phase change drywall panels (Figure 15) demonstrate effective thermal regulation through latent heat storage, contributing to improved energy efficiency and enhanced comfort.

### 11.3. Walls

The integration of PCMs in building walls enhances thermal storage capacity and improves energy efficiency. Several studies have demonstrated the effectiveness of PCMs in regulating indoor temperature and reducing energy consumption.

- Radiant Wall Integration:

Plytaria et al. [205] implemented PCMs within the radiant wall structure of a solar-powered cooling system in a building in Athens (Greece) to analyze energy demand and total system investment. The cooling system relied on evacuated tube solar collectors in conjunction with a single-effect absorption chiller. PCMs were integrated across all exterior-facing walls and their performance was examined under different operating scenarios, including selective application on certain facades. A range of solar collector surface areas and thermal storage volumes were investigated to identify the most efficient system configuration. The results demonstrated that PCM inclusion boosted the thermal storage potential, thereby minimizing the operational frequency of the chiller and enhancing overall system performance by decreasing reliance on auxiliary energy. Positioning the PCM layer on the south-facing wall yielded the greatest benefits, with a 30% cut in supplementary energy usage, a 3.8% rise in solar energy utilization, and a 3% drop in system expenditure. All configurations were found to maintain satisfactory thermal conditions. Although the financial savings were relatively modest, the strategic integration of PCMs had a positive effect on the system’s energy and economic efficiency.

- Multi-Layer Wall Systems:

Sun et al. [37] constructed three PCM wall prototypes featuring distinct configurations to assess their thermal behavior during both summer and winter seasons. The experimental results were used to validate a mathematical simulation by comparing the thermal response of the PCM-enhanced walls against a control wall. The analysis focused on internal surface temperatures, daily heat transfer rates, and the walls’ thermal inertia. During the summer, the maximum variation in interior surface temperature was lowered by 21.4%, while in the winter, a reduction of 23.9% was observed. Thermal inertia increased by 60.3%, from 1.24 to 2.0. PCM spheres incorporated into the first, second, and third layers led to annual energy savings of 17.7%, 20.2%, and 23.1%, respectively. The third layer was the most efficient in summer, the fourth was the most effective in winter, and the third provided the best performance overall.

Kant et al. [206] performed a computational investigation of a building wall, examining the melting rate of the PCM and the heat flux on the interior surface. To effectively decrease heat ingress into the indoor environment, the PCM layer was recommended to be positioned between fiberglass insulation layers, featuring a melting point aligned with the indoor temperature and a greater thickness. Simulations that utilized actual weather data over a period of three days revealed that the implementation of the PCMs RT-25, RT-26, RT-28, and n-octadecane led to heat transfer reductions of 33.18%, 33.94%, 34.40%, and 37.13%, respectively.

- Numerical Simulations:

Khan et al. [207] evaluated the impact of a PCM on building wall heat transfer and indoor thermal comfort by fabricating two building-wall-fragment models. One model assessed various PCM layer placements to determine the optimal configuration, while another tested materials (PCM, air gap, sand, and brickbat) individually. The results showed that positioning the PCM layer closer to the heat source resulted in a lower temperature rise within the cold-water bath, compared to when it was placed near the heat sink. With the PCM, temperature increases in the cold-water bath were minimized and even showed a decreasing trend, highlighting that PCMs effectively reduce heat transfer through building walls and improve indoor thermal comfort.

Yu et al. [208] analyzed a selection of PCMs for application on the inner surface of solar-passive, sun-exposed building walls, using Energy Plus^®^ simulations that were calibrated with real-world data from Hebei Province (China). The simulation results identified the mmGH-37PCM panel as a viable choice, featuring a phase transition range from 37.4 °C to 43.5 °C and a latent heat of 227.5 kJ/kg. The use of PCM raised the internal surface temperature by 1.3 °C at night and 2.7 °C on a monthly average. Heating demand was lowered by 6.4 GJ in spring, 5.8 GJ in autumn, 3.4 GJ in summer, and 2.9 GJ in winter. Additionally, steam consumption declined by 4.7% annually, notably reducing the building’s heating load.

Kishore et al. [209] conducted a computational analysis to determine the optimal parameters for minimizing heat gains in the cooling season and losses in the heating season through PCM integration into walls. The study covered five U.S. cities across different climate zones and found that improper PCM application could increase energy consumption. However, optimized PCM integration could reduce annual heat gains by 3.5 to 47.2% and heat losses by 2.8 to 8.3%, depending on the climate.

- PCM Placement and Performance:

Zaid et al. [210] applied PCM to clay–straw walls in Drâa-Tafilalet (Morocco) to reduce building energy consumption. Heat storage and transfer were assessed by measuring the temperatures of the walls’ internal and external layers. The results showed that incorporating PCMs into the clay–straw walls lowered surface temperatures by up to 3 °C and reduced heat flow by approximately 32% compared to those of conventional walls. Walls with external PCM layers demonstrated a 26.5% reduction in maximum heat flux, while PCM-integrated walls achieved an intake heat flux of about 14.2 W·m^−2^. Placing PCMs closer to the heat source resulted in a 1 °C reduction in surface temperature compared to that with outer wall positioning.

- State-Space Model for PCM Walls:

Ouhsaine et al. [211] proposed a state-space model for a solar-effective wall incorporating PCMs, offering a straightforward configuration of internal nodes and input/output settings. A key advantage of this approach was its computational efficiency, avoiding complex numerical procedures. The model was applied to a multi-layer wall with PCM wallboards positioned between the interior and the external environment, enabling the development of a novel wall design. Initial experiments focused on the emissivity of the radiative plate and the homogeneous temperature distribution of the MHP. A mathematical model of the RC-PCM wall was then formulated, and numerical analyses evaluated the effects of external wind speed, PCM thickness, and radiative plate emissivity. The results showed that the inner surface temperature of the RC-PCM wall was inversely related to wind speed and positively correlated with emissivity. The cooling load of the RC-PCM wall was 25% lower than that of a brick wall of a similar thickness, reaching a 42% reduction under optimal conditions, demonstrating the effective use of PCM. The model effectively predicted the system’s thermal behavior and the impact of PCM placement on the inner wall surface, significantly stabilizing indoor temperatures and enhancing thermal comfort.

- Shape-Stabilized PCM in Plaster:

Hattan et al. [212] investigated the effect of shape-stabilized PCM (SSPCM) on the thermal behavior and mechanical strength of masonry walls in Iran. PEG 600 was vacuum-impregnated into porous silica fume to stabilize its shape. Cement mortars with SSPCM were applied to plaster masonry walls, showing that although compressive strength decreased, it remained within acceptable limits for plastering. The SSPCM-plastered walls reduced peak temperatures and indoor temperature fluctuations, improving thermal performance.

Yu et al. [213] tested the radiative plate emissivity and simulated the thermal behavior of MHP-RC-PCM walls. While the PCM’s latent heat had a minor effect on cooling load reduction, the phase transition temperature significantly influenced the liquid fraction and surface temperature. At 31 °C, the cooling load reduction was 4% higher than it was at 26 °C. The MHP-RC-PCM wall achieved 18.2% greater annual energy savings compared to a conventional brick wall and 0.4% more than a PCM-integrated wall in Guangzhou, China.

Alharbey et al. [214] evaluated bio-PCMs made from plant-based materials, which are more sustainable than petroleum-based PCMs. Incorporating bio-PCMs reduced annual energy consumption by 28%, with Q21 and Q23 reducing cooling energy by 23.4% and 22.1%, respectively. Considering metabolic rates between 99 and 180 W per person, the PCMs significantly lowered overall energy demand.

Imafidon and Ting [215] studied honeycomb PCM in retrofitted construction walls in Ottawa, Canada. A 1 cm-thick PCM with a melting point of 20 °C reduced heat losses by 96% and heat intake by 41%, maintaining stable indoor temperatures even with outdoor variations between −14 °C and 26.5 °C.

Nizovtsev and Sterlyagov [216] examined the effect of a PCM layer in foamed polyurethane walls. A 4 vol.% paraffin layer reduced heat flux variations on the internal surface by up to 13 times, depending on PCM positioning. The highest reduction occurred when the PCM was placed at the wall’s center, increasing the time lag between peak outdoor temperature and peak heat flux by up to 8 h.

Gencel et al. [21] developed PCM-impregnated wall sheets with dodecyl alcohol. The treated walls maintained comfortable temperatures for up to 10 h, with a 2 °C difference between test and reference rooms.

### 11.4. Windows and Shutters

**Figure 16:**
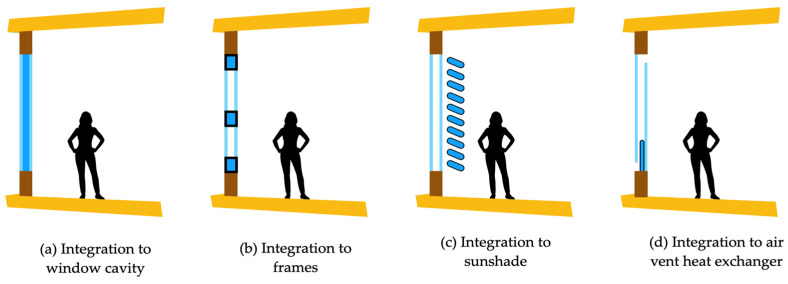
Possible routes to integrate PCM in windows and shutters.

There are several possible routes to integrate PCMs into windows and shutters. Figure 16 illustrates some of those.

The authors Sudha et al. [217] conducted an experimental study to evaluate the thermal behavior of a twin glass window in comparison with a PCM-integrated twin glass window unit, in which PCM (OM420) was introduced into the cavity between the glass panes. The study demonstrated that embedding PCM within the twin glass structure could notably minimize indoor heat gain, thereby promoting a more consistent indoor temperature and reducing reliance on air conditioning systems, leading to potential energy savings. The peak indoor air temperature was lowered by 5.6 °C, and the time to reach this maximum was postponed by up to 2 h. These results indicated that twin glass windows incorporating PCM represent a viable strategy for energy-efficient building design.

Furthermore, the authors Jiang et al. [218] carried out a study on the thermal efficiency of windows equipped with PCM-filled frames in rural homes situated in extremely cold regions of China. The thermal behavior of both the glazing and the frame was simulated and evaluated using Energy Plus^®^, and the heating energy usage of different window configurations was compared. The investigation also explored the influence of various window sizes for both southern and northern orientations, in addition to assessing frame structure variations on energy performance. Four types of frames were analyzed: plastic, aluminum alloy, plastic–steel, and plastic–steel with PCM integrated into the frame cavities (PSFP). These were paired with windows of varying window-to-wall ratios. The PSFP frame configuration proved efficient in lowering energy use. The incorporated PCM functioned by absorbing excess thermal energy during the day and releasing it at night through phase change, aiding in indoor heating. Findings showed that integrating PCM into window frames could decrease heating energy consumption by up to 20% in harshly cold climates. Additionally, a short payback period of just 5.2 years rendered the PSFP design a highly attractive solution for improving building energy efficiency.

Silva et al. [219] conducted a full-scale outdoor experimental investigation on the use of PCM shutters to improve energy performance and indoor thermal comfort. The peak indoor temperature in the compartment with the PCM shutter was measured at 37.2 °C, which was 16.6 °C lower than that of the reference compartment. The results indicated that the inclusion of a PCM-based shutter could efficiently stabilize indoor temperature and lower thermal peaks. The authors emphasized that tailoring the PCM’s phase change temperature to the specific regional climate is crucial, as extended periods in a fully melted state may compromise its insulating properties and liquid phase, which could negatively impact thermal insulation. This underscores the importance of adapting PCM characteristics to local conditions to maximize energy savings and indoor thermal comfort.

In addition, Bianco et al. [220] developed a solar shading system incorporating PCM for thermal regulation and daylight control, while also improving the thermal mass of the transparent building envelope. The system featured alveolar polycarbonate panels embedded with PCM, and two prototype versions were tested in Turin, Italy. Results showed that the PCM shading solution could significantly diminish solar heat gains, achieving a daily energy saving of up to 40% compared to a non-PCM system. Furthermore, during the hottest hours of the day, the internal surface temperature of the PCM-integrated shutter was 4 to 5 °C lower. These outcomes confirm that applying PCMs in dynamic shading systems offers an effective strategy for controlling indoor climate and reducing building energy demand.

King et al. [221] examined the thermal performance of a double-glazed window with paraffin PCM, which was placed in the existing gap between the double-glazing units for an entire representative day. The research team analyzed indoor temperature, inner glass temperature, energy consumption, and the transmittance of the glass combined with PCM. The authors reported that the transmittance remained above 0.7 during daytime operating temperatures, allowing a significant amount of light energy into the room. They also found that incorporating PCM in the double-glazed window reduced indoor temperature fluctuations, maintaining values between 21 °C and 11 °C. Furthermore, it lowered the indoor peak temperature by 9 °C. The inner glass temperature was reduced by 8.5 °C with PCM, leading to an approximate 3.8% reduction in energy consumption through the double-glazed window.

Furthermore, the researchers Xu et al. [222] introduced a leak-resistant phase change window system utilizing a gel-based PCM with high latent heat, formulated from n-octadecane and styrene-b-ethylene-b-styrene. This composite gel demonstrated superior sealing performance and improved thermal cycling stability when compared to pure n-octadecane. A double-glazed unit was infused with this gel to fabricate a novel phase change gel window (PCGW). Unlike traditional PCM windows, the gel in the PCGW remained contained during the phase change, while maintaining comparable light transmittance in its liquid state.

The findings revealed that the PCGW offered enhanced thermal insulation relative to standard PCM glazing. Moreover, the thermal regulation performance of the system was influenced by both gel layer thickness and solar radiation intensity. Increasing the gel thickness from 4 mm to 8 mm led to a 5.9% reduction in the maximum inner surface temperature after 120 min of exposure to 700 W/m^2^ of solar radiation. In contrast, raising the solar intensity from 600 W/m^2^ to 800 W/m^2^ caused the peak surface temperature to rise by about 3.3 °C.

The maximum temperature of the PCGW’s inner surface was 1.3 °C lower than that of the conventional PCM window (PCMW) under 800 W/m^2^ of solar radiation intensity for 120 min. The indoor temperature with the PCGW was approximately 1.7 °C lower than that of the PCMW. Moreover, the time required to reach 37 °C for the PCMW was nearly 21% shorter than that for the PCGW, demonstrating the latter’s superior thermal regulation performance.

**Figure 17:**
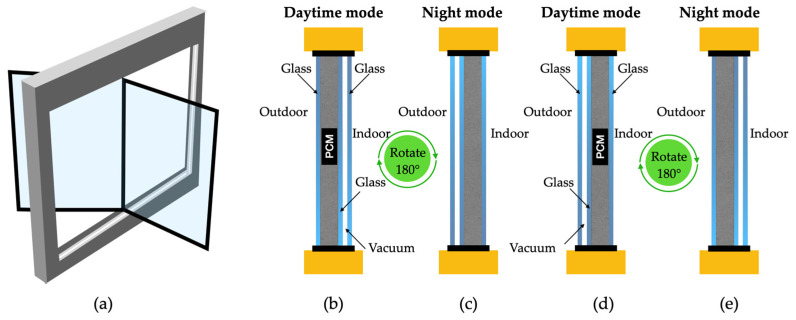
Rotating window integrating PCM: (**a**) window; (**b**–**e**) possible operational modes of rotating window with PCM. Adapted from [223].

Zhang et al. [223] evaluated the thermal performance of a rotatable dynamic window integrated with PCM and vacuum glass layers. The dynamic PCM window (DPCMW) could adjust the relative position of the PCM and vacuum glass layers according to the time of day, enabling the direction of heat flux to be altered to reduce the building’s thermal load. The authors concluded that an excessively high phase transition temperature in winter was not conducive to phase change heat storage, while a phase transition temperature that was too low in summer hindered the outdoor heat transmission of the DPCMW at night. The optimal phase change temperature throughout the year ranged between 23 °C and 25 °C. Additionally, the study indicated that a thicker PCM layer enhanced both the thermal resistance and heat storage capacity, resulting in a greater energy-saving effect. The heating loads of DPCMWs with PCM layer thicknesses of 6, 9, 12, 15, and 18 mm were approximately 49.4, 46.1, 43.5, 41.7, and 40.3 kW·h/m^2^ in winter, respectively, while the cooling loads were approximately 5.8, 4.1, 2.9, 1.9, and 1 kW·h/m^2^ in summer. The authors also reported that expanding the PCM phase change range could decrease the cooling load in summer but increase it in winter, with a more pronounced influence on cooling loads. This occurred because a lower PCM melting temperature was not conducive to the exothermic solidification of the DPCMW. The optimal PCM phase transition range throughout the year was between 21 °C and 25 °C. The results demonstrated that the DPCMW exhibited better energy-saving performance in both winter and summer. The heating load of the DPCMW was approximately 40.5 kW·h/m^2^, nearly 5.7 kW·h/m^2^ lower than that of the static PCM window (SPCMW), representing a reduction of 12.4% in winter. In summer, the cooling load of the DPCMW was approximately 0.7 kW·h/m^2^, 10.9 kW·h/m^2^ lower than that of the SPCMW, corresponding to a reduction of 93.8%. Under annual operating conditions, the DPCMW reduced the building thermal load by 28.7% compared to the SPCMW. Figure 17 schematically represents the studied rotating window integrating PCM.

**Figure 18:**
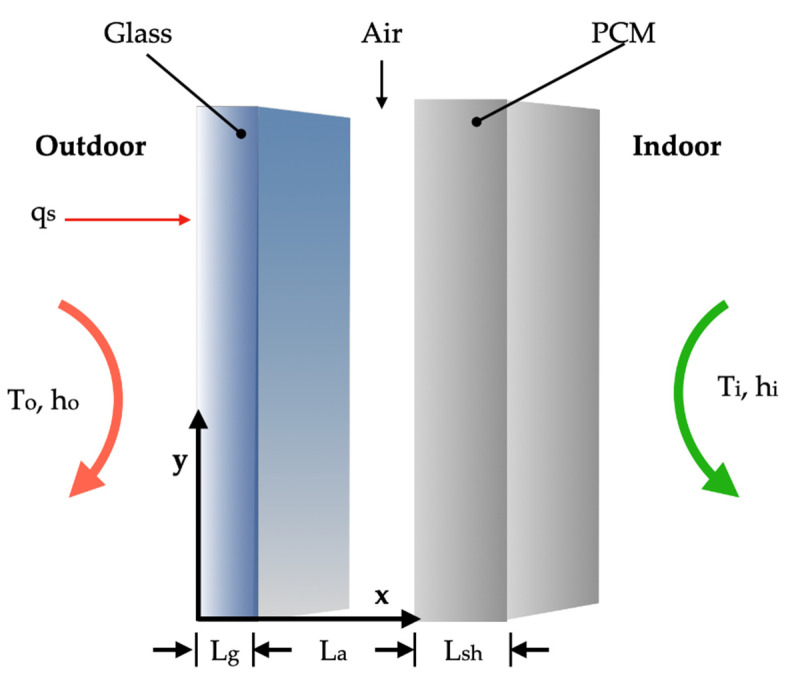
A schematic of the window system with a PCM curtain, as used by Wang and Zhao [224].

Figure 18, in sequence, illustrates the window system with a PCM curtain, highlighting dimensions and variables such as the thickness of the glass, curtain, and air gap and the PCM melting temperature, which were tested by Wang and Zhao [224] to assess the energy efficiency of buildings. In the diagram, the left side of the window system (glass) is exposed to external conditions, while the right side (curtain) is subjected to indoor conditions. The outdoor air temperature (T_o_) and indoor air temperature (T_i_), heat transfer coefficients for the outer surface (h_o_) and inner surface (h_i_), and the intensity of solar radiation (q_s_) are also indicated. L_g_ is the thickness of the glass (5 mm), and L_c_ and L_a_ are the thicknesses of the curtain and air gap, respectively.

The results obtained by the researchers showed that using a PCM curtain within the window system could significantly reduce solar heat gain during the summer. The main observed effects were the following: (i) Air Gap Effect: Natural convection was enhanced with thicker air gaps, which negatively impacted the reduction in heat gain. (ii) PCM Melting Temperature: The PCM melting temperature was crucial for the system’s effectiveness and had to be optimized based on simulation results. The proper selection of the melting temperature was essential to maximize heat gain reduction. (iii) PCM Layer Thickness: Thicker layers of PCM, with the correct melting temperature, helped reduce heat gain. It was observed that on the hottest summer days in Shanghai, implementing a 15 mm-thick PCM layer with a phase change temperature of 29 °C led to a reduction of up to 30.9% in the average heat flux entering the indoor environment.

These findings imply that the proposed technique presents a promising strategy for minimizing solar heat gains in buildings throughout the summer season.

The researchers Lu et al. [225] improved the energy efficiency and photo-thermal performance of a double-layer PCM glazing window (DP). To further enhance its performance, multi-layer PCM glazing windows integrating DP with additional installations and low-emissivity (low-e) coatings were developed. However, the energy efficiency of a multi-layer glazing window is not always higher than that of DP across all climate zones. The selection of the optimal DP configuration—i.e., the most energy-efficient multi-layer glazing window—should consider the specific climatic conditions. To investigate the thermal and energy performance of these configurations, physical heat transfer analyses and mathematical models were developed and simulated using Ansys Fluent across different climate zones in China. Evaluation indexes were established for each climate zone, and the energy-saving potential of various PCM glazing window designs was compared. The most energy-efficient configuration in each climate zone was identified as the optimal DP strategy. The results demonstrated that DP with an external silica aerogel layer was the optimal strategy for severe cold zones, achieving an energy savings of 40.28%. However, this configuration increased energy consumption in mild zones, as well as in regions with hot summers and warm winters. Conversely, DP with an external air layer and internal low-e coating was identified as the optimal strategy for cold zones, hot-summer and cold-winter zones, and hot-summer and warm-winter zones, with energy-saving potentials of approximately 40.7%, 46.4%, and 47%, respectively. However, this configuration led to increased energy consumption in mild and cold zones. Additionally, DP was found to exhibit the lowest energy consumption in mild zones, making it the most suitable choice for such climates.

Al-Yasiri et al. [34] analyzed the impact of natural night ventilation (NNV) through a single window on enhancing the thermal performance of a room with PCM, taking into account both window orientation and the window-to-wall ratio (WWR). The research explored the efficacy of NNV via a one-directional window setup to demonstrate the potential of passive cooling strategies during summer conditions. Using an Energy Plus® simulation, various orientations and WWR values ranging from 8.75% to 20% were evaluated to determine the optimal indoor thermal performance. The outcomes showed that window orientation had a minimal effect on NNV in PCM-equipped rooms, regardless of prevailing wind direction. Nevertheless, windows facing the northeast delivered the greatest indoor temperature drop, reaching up to 31%, along with a thermal load balancing improvement between 9% and 17%. Furthermore, increasing window size enhanced thermal comfort, with a 20% WWR lowering the average indoor temperature by 1.14 °C compared to the reference room (WWR = 8.75%) without ventilation. At the largest window configuration, the operative temperature dropped by as much as 22% during nighttime. The study concluded that although NNV is advantageous, its performance is limited in extremely hot climates, where alternative or supplemental cooling methods may be required.

Wijesena et al. [226] developed shape-stabilized PCMs using chitin nanofibers incorporated into polyethylene glycol. The addition of chitin nanofibers to polyethylene glycol resulted in the formation of stabilized composites, which were then encapsulated in an optical device to achieve temperature-dependent transparency. In the optimized formulation, the device remained opaque (~3.5%) below the composite’s melting point and gradually became transparent as the temperature increased, ultimately stabilizing at a transmittance value of approximately ~88%.

Zhang et al. [227] investigated the development of thermally responsive smart windows with passive dimming and enhanced thermal energy storage capabilities. By simulation three-dimensional networks of polymer hydrogels, thermally responsive PCMs (TRPCMs) were specifically designed for energy-efficient windows. In this approach, tetradecanol and a thermochromic dye were incorporated in situ into poly (*n*-butyl isobutyrate) (PBB) networks. The TRPCMs converted solar energy into thermal energy through the color change in the dye, which transitioned from blue to colorless and transparent at approximately 45 °C. Simultaneously, the tetradecanol PCM absorbed heat and transitioned to a a transparent liquid state. As a result, the TRPCMs shifted from an opaque state at room temperature to a high-transparency state upon melting (~75%). Moreover, these materials exhibited a significant thermal storage capacity, with a phase transition enthalpy exceeding 161.9 J/g. The as-prepared TRPCMs enabled simultaneous photothermal conversion, thermal energy diffusion, latent heat storage, and liquid leakage resistance at the phase transition interface between the opaque and transparent states, offering new possibilities for the design of energy-saving buildings.

### 11.5. Floors

**Figure 19:**
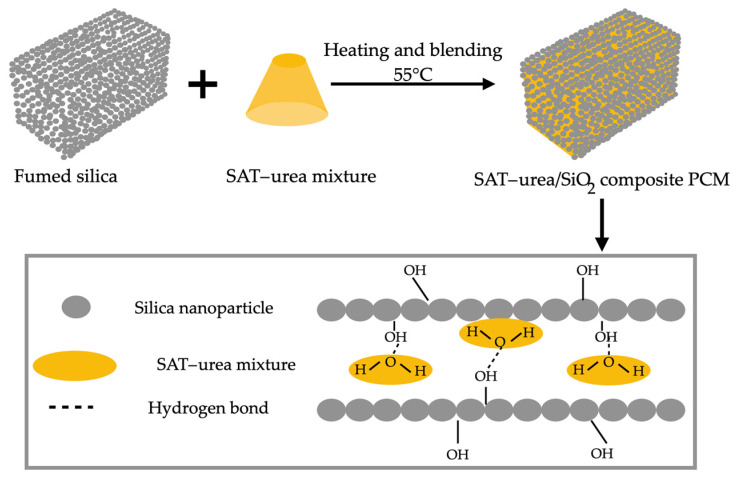
Fundamental steps in the preparation method of the composite PCM. Adapted from [228].

Fu et al. [228] synthesized a phase change temperature-tunable composite PCM using sodium acetate trihydrate, urea, and fumed silica. The phase change transition temperature of the composite PCM was adjustable to approximately 34.4 °C and 48.5 °C, effectively reducing supercooling and preventing leakage. With silica content of 30 wt.%, the composite PCM exhibited a favorable melting temperature of around 35.8 °C, a high latent heat of 151.6 kJ/kg, and a low supercooling degree of 1.14 °C. Additionally, the composite PCM demonstrated stable form retention and good thermal conductivity, indicating its enhanced suitability for application in radiant floor heating. Figure 19 illustrates the main steps involved in the preparation of the composite PCM.

Tang et al. [229] proposed a PCM floor by incorporating paraffin and expanded graphite into building cement and evaluated its thermal performance in a solar–heat pump hybrid radiant heating system. The mass fraction of the composite PCM was optimized at 35%, considering thermal storage capability, thermal stability and conductivity, leakage resistance, and mechanical strength requirements. The application potential of the PCM floor was further assessed through experimental measurements and TRNSYS simulations. The results demonstrated that the thermal comfort satisfaction rate of the room equipped with the PCM floor reached 96.8%.

González and Prieto [230] conducted a computational fluid dynamics (CFD) analysis of hydronic radiant heating floors with PCM bands embedded in a concrete core, considering multiple configurations based on the bandwidth and position relative to the heating pipes. The findings indicated that the PCM-integrated radiant floors enhanced thermal energy storage by up to 243% and reduced heat flux by 10–18%, depending on the configuration. Moreover, these floors released heat gradually after the heating system was turned off. The time-averaged heat flux during the solidification process ranged between 31.4 and 44.6 W/m^2^, with the solidification phase lasting over 24 h, allowing sufficient time to initiate a new charging cycle, such as one using solar energy.

Ju et al. [231] investigated a heating configuration featuring a PCM-integrated radiant floor system combined with a horizontal ground-source heat pump (PCM floor–HGSHP, Case 1) to ensure indoor thermal comfort and enhance energy performance. The PCM floor was simulated using a TRNSYS module, and a rule-based control strategy was designed, incorporating time-of-use electricity pricing to optimize system efficiency. A case study was performed on a single-family residence to evaluate the system’s applicability in colder climates across China. Several representative cities were selected, and two reference scenarios were defined for comparative analysis using key performance indicators. Results demonstrated that, when compared to a traditional radiant floor system using an HGSHP (RFHS-HGSHP, Case 2), the PCM-enhanced setup in Case 1 improved indoor thermal stability by 8.6%, enhanced load-shifting capability by 18.1%, and lowered operating costs by 13.8%. Additionally, when compared to a system integrating PCM flooring with an air-to-water heat pump (PCM floor–AWHP, Case 3), substituting the AWHP with the HGSHP led to a 9.6% longer duration of thermal comfort and a 33.7% decrease in energy usage. These findings confirmed the system’s strong performance in terms of both thermal comfort and cost efficiency, verifying its technical feasibility and practical benefit in various regions.

Park and Kim [232] analyzed a PCM-based radiant floor heating system utilizing hot water as a heat source, which could be integrated with the widely adopted wet construction method. Their study identified the optimal PCM melting temperature for the proposed system to be between 35 °C and 45 °C, considering a floor thickness of 70 mm and a PCM layer of 10 mm. Mock-up test were conducted to compare the performance of the radiant floor heating system with and without PCM. The results indicated that the PCM-integrated room maintained a temperature 0.2 °C higher than that of the room without PCM. This improvement was attributed to the PCM’s ability to storage and release heat, reducing heat loss to the underside of the hot water pipe when PCM was present.

Babaharra et al. [233] conducted a numerical evaluation of the thermal performance of a heated floor embedded with microencapsulated PCM. The system consisted of hot water circulating through tubes buried beneath multiple ground layers. Thermal waves propagated from below, heating the floor and subsequently transferring heat to surrounding space. A two-dimensional computational model, based on the enthalpy method and the finite-volume numerical approach, was developed to assess the PCM-enhanced underfloor heating system. Various parameters, including microcapsule integration, mass fraction, tube spacing, and PCM type, were analyzed to determine their impact on thermal performance. The results demonstrated that the optimal configuration involved 15% microcapsules positioned above the heating tubes, leading to an increase of 4 °C in temperature amplitude and a phase shift of 5 h and 30 min compared to a conventional heated floor.

Lu et al. [234] developed an innovative PCM floor integrated with a solar water heating system (PFCSS). A TRNSYS simulation model of the PFCSS building was established and validated using full-scale experimental data. Two experimental buildings—a reference building and a PFCSS-equipped building—were constructed in Ninghe County, Tianjin (China). A comparison between experimental and simulated data for the PFCSS building showed a mean relative error of 0.6%, while the Bland–Altman consistency analysis yielded a 95.1% agreement within a 95% confidence interval. To assess thermal performance, a model of the reference building exhibited lower temperature fluctuations and an extended heating duration compared to those of the reference building. When maintaining an indoor temperature of 20 °C, the PFCSS achieved energy savings of approximately 5.9% relative to the reference building.

Sun et al. [235] investigated the suitability of double-layer radiant floors incorporating two inorganic PCM layers positioned at different locations to regulate indoor thermal comfort in both winter and summer conditions. To enhance thermal conductivity, hydrated salts with distinct melting temperatures were compounded with expanded graphite. In the proposed floor system, Na_2_HPO_4_·12H_2_O (melting at 31.3 °C) was used for the heat storage layer, while CaCl_2_·6H_2_O (melting at 20.2 °C) served as the cold storage layer. Under winter conditions, the room with the radiant floor system—featuring an upper heat storage layer and a lower cold storage layer—maintained thermal comfort for a duration 2.2 times longer than that of the reference room, which had a pebble-filled floor. Notably, reversing the positions of the two layers improved thermal comfort in summer, extending its duration to 8.1 h—1.7 times longer than that in the reference room. Furthermore, optimizing the thermal conductivity of the PCM composites contributed to energy savings by reducing the operating time of active heating and cooling systems. The study suggested that double-layer radiant floor systems can lower economic costs by effectively shifting peak loads.

## 12. Dynamic Energy Modeling of PCM-Enhanced Passive Buildings

The use of whole-building energy simulation is an essential step in evaluating and analyzing the performance of PCM-enhanced buildings. These tools can numerically assess the thermal performance of buildings passively enhanced with PCMs. Currently, a variety of validated whole-building energy simulation tools are capable of conducting dynamic performance analyses for multiple applications [236]. Nonetheless, only a limited number of these tools have been verified to assess both the thermal behavior and indoor comfort conditions of buildings integrated with PCM-based passive strategies.

### 12.1. Software

This section outlines the most widely adopted and impactful simulation platforms utilized for PCM-based passive cooling design. Tools such as Energy Plus^®^ [237], TRNSYS [238], ESP-r [239], and Ansys Fluent [240] have been extensively applied by researchers to investigate PCM performance in building environments, with multiple studies carried out to verify the reliability of these simulation tools [241].

#### 12.1.1. Energy Plus

Energy Plus^®^ is an open-source, multi-platform building energy performance modeling tool that incorporates the most common capabilities of DOE-2.1E and BLAST, along with many advanced characteristics. Additionally, it allows for the development of innovative modules and control approaches, which can be integrated into the program as subroutines. Through its energy management system, Energy Plus functions as a dedicated software that can be programmed to regulate building energy systems, including cooling, ventilation, heating, hot water, and interior and exterior lighting, as well as shading devices, windows, shutters, and double-facade elements [242,243]. Moreover, the FMU or Functional Mock-up Unit for the co-simulation import interface enables Energy Plus^®^ to conduct co-simulation with various simulation programs packaged as FMUs [244]. Another key skill of this software include fenestration analysis, envelope performance evaluation (considering both exterior and interior surface convection algorithms), advanced infiltration and ventilation modeling, room air and multi-zone airflow determinations, environmental emissions assessment, and economic evaluations that encompass overall energy costs and life cycle costs. Furthermore, compared to other simulation tools, Energy Plus^®^ integrates several advanced human thermal comfort algorithms for analyzing the thermal comfort of the occupants and quality of the indoor air. Additionally, multiple graphical interfaces are available to facilitate the use of this software [245].

Zhuang et al. [246] conducted a study titled “Validation of Veracity on Simulating the Indoor Temperature in PCM Lightweight Building by Energy Plus”. This work examined the construction solution algorithm and heat balance method in Energy Plus^®^, presenting the conduction finite-difference solution algorithm and enthalpy–temperature function features. It also described the implementation of the module and validated the experimental data. The highest relative difference between the simulation and experimental values was 12.41%, while the lowest was 0.71% over a 36 h period under Condition A. Under Condition B, the highest relative difference was approximately 8.4%, and the lowest was around 0.3% over a 72 h period. The results obtained showed good agreement with well-established simulation tools and demonstrated that the algorithm incorporated into Energy Plus^®^ can effectively simulate PCM in building construction.

Moreover, Pandey et al. [247] stated that BES or building energy simulation features are fundamental tools for dimensioning PCM-based systems in the built environment and analyzing their impact on thermal comfort and energy demand. However, due to the complex solidification and melting phenomena of PCM, BES tools have limitations in accurately predicting their performance in buildings. Computational fluid dynamics (CFD) tools have also been used to model PCM behavior, but these models are not integrated with building simulation models. The work aimed to devise a co-simulation framework between the BES tool Energy Plus^®^ and the CFD tool Ansys Fluent to model PCM-integrated buildings and compare its estimation precision accuracy with that of Energy Plus^®^. Three different scenarios were modeled for prediction accuracy assessment: the active use of PCM, passive use of PCM under natural convection, and passive use of PCM under forced convection. The root mean square error (RMSE) was employed alongside temporal temperature variations for comparison. The obtained results highlighted that the developed co-simulation framework provided superior prediction accuracy compared to the BES tool for both the active and passive usage of PCM under forced convection. However, for modeling the passive use of PCM under natural convection, the BES tool alone was recommended.

#### 12.1.2. TRNSYS

TRNSYS is a flexible transient system simulation program with a modular structure. It is a component-based simulation tool in which users select the components that make up the energy system and interconnect them, employing adequate input and output ports. The TRNSYS library is composed of diverse components designed for the simulation of buildings, HVAC systems, ventilation, solar energy, thermal energy storage, and routines to support the input of local weather information or other time-dependent functionalities, as well as the output of simulation results. Additionally, it allows for the integration of innovative mathematical models not originally incorporated in the software, enabling them to be coupled with the existing components. TRNSYS has become a widely used tool in the research community, capable of simulating low-energy buildings; solar, thermal, and photovoltaic systems; HVAC; renewable energy systems; fuel cells; cogeneration; and PCM-regulated systems [248].

Al-Saadi and Zhai [249] developed a new validated TRNSYS module for simulating latent heat storage walls. In their work, a new TRNSYS type was designed and validated for simulating PCM-enhanced walls. Using the validated module, they reported that the best PCM configuration was achieved when it was placed in direct contact with the indoor regulated environment. Furthermore, a wide range of PCM thermal properties were simulated under typical U.S. climates to evaluate thermal performance and identify the most suitable thermophysical characteristics. The results showed that annual cooling savings ranged from 0.8 to 15.8%, depending on the climate. In heating-dominated climates, the savings on annual heating loads were generally below 4%. The reduction in peak loads showed greater potential than annual load saving in some climates. The maximum savings in peak cooling loads ranged from 6.8 to 13.3%, while peak heating load savings varied between 7 and 10.5%. For maximum savings in zonal loads, the optimal thermal properties of PCM were found to be close to the operational thermostat setpoints.

#### 12.1.3. ESP-r

ESP-r is a general-purpose, multi-domain simulation program for building thermal performance, inter-zone airflow, intra-zone air movement, HVAC systems, and electrical power flow. It allows users to make good use of the complex relationships between the form, envelope, airflow, plant, and control systems of a building. ESP-r is based on a finite-volume conservation approach, where the problem is represented as a number of conservation equations that are integrated over consecutive time steps in response to climate conditions, occupant behavior, and control system influences [250]. In ESP-r, PCM can be modeled, exploring the special materials facility concept [251].

Heim and Clarke [252] proposed a numerical model and thermal simulation of PCM–gypsum composites using ESP-r. Their goal was to refine the ESP-r system by incorporating PCM modeling. The performance of PCMs was simulated using ESP-r’s special materials facility. The phase transition effect was incorporated into the energy balance equation as a latent heat generation term, following the so-called effective heat capacity method. The numerical simulations were conducted for a multi-zone, naturally ventilated passive solar building, where PCM-impregnated gypsum plasterboard was used as an internal room lining. Air, surface, and room temperatures were compared with a reference case without PCM, and the latent heat storage effect was analyzed. While this effect did not significantly reduce diurnal temperature fluctuations, the PCMs effectively stored solar energy during transition periods. Additionally, the energy requirement at the beginning and end of the heating season was estimated and compared with ordinary gypsum wallboard. In this comparison, the PCM composite solidification temperature was 22 °C, 2 degrees higher than the room’s heating setpoint. The results indicated that solar energy stored in the PCM–gypsum panels reduced heating energy demand by up to 90% at certain times during the heating season.

#### 12.1.4. Ansys Fluent

Ansys Fluent is a globally well-known and used software, being of general purpose in heat transfer and temperature distribution fields and the determination and interpretation of thermophysical properties of materials. It is also very useful in evaluating the thermal performance of PCMs in buildings.

Al-Mudhafar et al. [253] used this software to evaluate the potential of integrating PCMs in a residential building envelope in Iraq to diminish the cooling energy consumption. A three-dimensional building model was used to study the effect of using PCMs on reducing heat gain in the interior of the building. Two buildings were simulated for cases with and without PCMs. The results showed that a building integrated with PCM had better thermal performance than that without PCM. The internal air temperature when PCM was used lower than that without PCM. Additionally, the peak interior air temperature reduced and shifted by around 5 h, thereby reducing the heat gain and electric energy consumption. Moreover, a two-dimensional wall model was employed to study the integration of PCMs in the external wall. The wall model consisted of three layers. Different types of PCMs were used to discover a suitable PCM for selected weather data. The results showed that PCM with a higher melting temperature was suitable for hot-weather conditions. When an external layer of PCM with a 35 °C melting temperature was used, the peak internal wall surface temperature and peak heat gain reduced, which demonstrated it to be more efficient for locations with hot weather including cities like Baghdad and Basra.

Rucevskis et al. [254] used the CFD Ansys Fluent software for the parametric analysis and design optimization of a PCM thermal energy storage system. The design for a new space cooling system proposed a TES system composed of stand-alone PCM storage units incorporated into the building interior under the ceiling slab. The active control of the thermal energy storage was realized by night cooling of a PCM by the means of cold water flowing within a capillary pipe. The objective was to determine the influence of the system’s main parameters including the PCM layer thickness, number of parallel pipes, diameter of pipes, night cooling duration, cooling water inlet temperature, and velocity under Baltic summer weather conditions.

Hadjadj et al. [255] explored Ansys Fluent to study new passive techniques and PCM integration in building envelopes for improved energy efficiency in desert regions. The passive techniques included PCMs, thermal insulation materials, and low-emissivity coatings—all integrated into hollow clay bricks to enhance the bricks’ thermal storage capacity and insulation resistance under the hot climatic conditions of Bechar, Algeria. Using a two-dimensional computational model, Capric acid, RT-42, and RT-27 PCMs encapsulated in steel capsules were studied, alongside EPS insulation and low-emissivity coating directly integrated into the brick cavities. The authors concluded that integrating capric acid PCM in hollow clay bricks considerably enhanced the thermal performance, decreasing the temperature and heat flux by 3.2 °C and 28.7%. Lowering emissivity significantly decreased the inner surface temperature by up to 6.4 °C, enhancing thermal resistance and stability, while also reducing inner heat flux by 57.3%, thereby improving insulation and energy efficiency. Filling cavities with EPS insulation proved highly effective, reducing inner surface temperatures by 3.9 °C and internal heat flux by around 35% and delaying peak heat flux by 2 h compared to those in conventional bricks. Combining capric acid at the bricks’ centerline with EPS in upper cavities and low-e coatings in lower cavities reduced the inner surface temperatures by nearly 6.6 °C and heat flux by 58.4% in comparison to in cavities filled with air, resulting in a huge energy saving of around 56.2%.

Kheradmand et al. [256] employed Ansys Fluent to numerically evaluate a hybrid PCM (composed of three distinct PCMs) embedded in plastering mortars for facade walls, aiming to achieve enhanced thermal performance in buildings. A numerical simulation model was explored to validate the capability of simulating the temperature evolution within the prototype with hybrid PCM and to understand the contribution of hybrid PCM to the overall energy efficiency of buildings. The authors reported that the inclusion of the hybrid PCM into plastering considerably decreased the cooling and reduced heating demands for maintaining the interior temperature within comfort levels in comparison to conventional mortars or mortars with only one type of PCM.

Al-Obaidi et al. [257] investigated the thermal behavior of a PCM in an optimized design of a cooling building envelope. The authors studied this behavior in a building envelope in the tropics and optimized the cooling effect by manipulating the melting temperature, thickness, and position and type of PCM. Ansys Fluent modeled the 2D cross-sectional area of the steel roof and brick wall. The results indicated that the ideal melting point of the PCM was 29 °C for the brick wall and 31 °C for the steel. When compared to a common day, the PCM reduced the indoor surface temperature by up to 4 °C.

Suhendri et al. [258] investigated the impact of a solar chimney and PCM cooling ceiling on the air flow inside a naturally ventilated building. This impact was numerically evaluated through the Ansys Fluent software. The results showed how the buoyancy effect was used to drive ventilation when using the solar chimney. Cooler air appeared from ceilings and cooled the room down. Meanwhile, warm air was concentrated at the top of the chimney and cooler air was concentrated in the bottom part of the chimney, generating wind flow across the building. With certain boundary conditions, the building could provide around 0.7 m^3^/s of fresh air.

### 12.2. Mathematical Modeling of PCMs in Building Energy Simulation Tools

The analysis of energy regulation and thermal comfort in buildings incorporating PCMs for passive cooling is highly influenced by factors such as the material’s melting point, quantity, thermophysical characteristics, placement within the building envelope, local climate, boundary conditions, and architectural design. Mathematical modeling plays a key role in optimizing system configurations and selecting the most appropriate PCM for passive applications [259]. Different numerical modeling strategies have been implemented in whole-building simulation tools to evaluate the thermal behavior of PCMs. These approaches are typically classified according to their mathematical formulation, representation of PCM, and spatial–temporal discretization techniques. Furthermore, numerous experimental, analytical, and comparative studies have contributed to the development of robust, validated models that enhance predictive accuracy.

### 12.3. Machine Learning

Artificial Neural Networks (ANNs) are powerful modeling techniques for both linear and nonlinear interactions, capable of capturing and representing complex input–output relationships [260]. The learning process involves minimizing the mean square error across all training patterns. Users can define an ideal outcome and compare the network’s performance against a target training dataset. In recent years, ANNs have been widely applied in various research areas, including the thermal performance of buildings.

**Figure 20:**
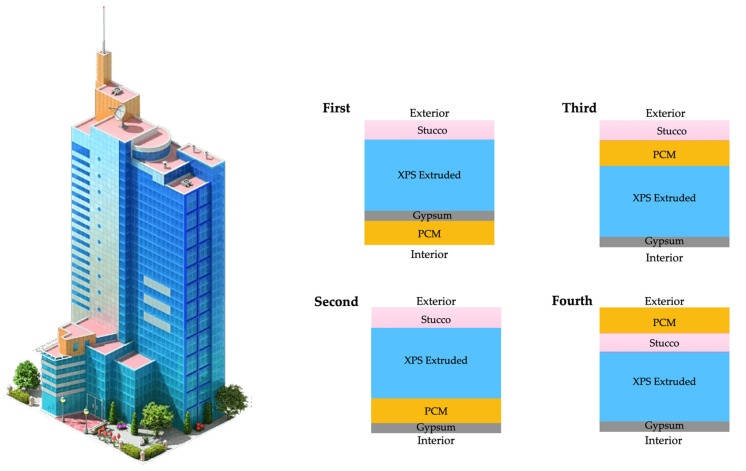
Four different cases of the composition of the walls and ceilings of the building. Adapted from [236].

Hai et al. [261] investigated the effectiveness of PCM placement within a budling envelope using an ANN. Their study employed a passive approach, where thermal dissipation from the building in winter and energy absorption in summer were reduced. The methodology involved installing a 20 mm-thick layer of SP21-EK PCM. The walls and the ceiling were made of the same materials to isolate the effect of radiation, consisting of gypsum, XPS, and stucco layers. The lower thermal conductivity, combined with the greater thickness of XPS within the ceiling and walls, resulted in significantly high thermal resistance. SP21-EK PCM was installed in four different locations. In the first case, the PCM was positioned in the innermost layer, directly exchanging heat with the interior. In the fourth case, it was placed in the outermost layer, in direct contact with the outside air. The results demonstrated that PCM placement in the first and second locations (before the insulation) yielded the most favorable outcomes. In contrast, when PCM was installed in the third and fourth locations (after the insulation), energy savings decreased significantly. For the second case, where PCM was embedded inside the wall/roof, energy savings of 14.9 kWh/m^2^ for walls and 19.6 kWh/m^2^ for the roof were reported. When considering the total building area, the overall energy savings reached 15.9 kWh/m^2^ (walls + roof). However, a significant reduction in energy savings was observed in the third and fourth configurations. The annual energy consumption of the building was approximately 64.236 kWh/m^2^, while the ANN model predicted 64.209 kWh/m^2^, with an error margin of less than 0.04%. A monthly analysis further showed that the prediction error for monthly energy consumption remained below 1.5%. Figure 20 shows the composition of the walls and ceilings for the four different possible cases for the building.

Abbasian-Naghneh and Kalbasi [262] investigated the application of an ANN and genetic algorithms (GAs) in optimizing energy savings and reducing carbon dioxide emissions in buildings incorporating PCM. Their study analyzed various setpoints, PCM types, and installation locations as input variables, assessing their effects on output variables such as cooling and heating loads, as well as annual energy consumption. The ANN model was used to establish correlations between input and output variables, trained with over 200 solutions. The results demonstrated that the effectiveness of PCM placement was highly dependent on the location of the thermal insulation installation. In most cases, when PCM was installed in close proximity to the thermal insulation (L3 position), a significant reduction in cooling load was observed. Furthermore, the findings indicated that the PCM should generally have been positioned closer to the indoor environment rather than the exterior. The genetic algorithm analysis further revealed that, to minimize annual energy consumption, the optimal indoor temperature should have been set at approximately 24.2 °C, with PCM 22 installed in the L3 position. Under these conditions, carbon dioxide emissions were reduced by 23.6%

Zhussupbekov et al. [263] employed machine learning techniques to predict the energy demand of residential buildings integrated with PCMs in Mediterranean climate zones. The study introduced a predictive model that simultaneously considered PCM melting point, building characteristics, and environmental parameters as input variables—an approach not previously explored. To develop the database, energy simulations were conducted for nine building typologies across seven cities within the Mediterranean region. The authors applied Multiple Regression (MR), a Support Vector Machine (SVM), and an Artificial Neural Network (ANN) to build and compare model performance. Parametric and sensitivity analyses were then carried out to identify the most influential design factors. Results revealed that optimal PCM performance for annual energy savings ranged between PCM-25 and PCM-27. Among the design parameters, the building shape factor showed a notable impact on both heating and cooling loads. Statistically, the SVM and ANN achieved the highest predictive reliability, with R^2^ values exceeding 0.99. External validation further confirmed that the ANN model delivered superior accuracy in estimating energy consumption. Sensitivity analysis identified cooling and heating degree days, building volume, shape factor, and PCM melting temperature as the most critical variables influencing energy demand.

Nazir et al. [264] predicted thermal performance of PCM-incorporated buildings by exploring optimized linear kernel and tree-based machine learning methods. The authors highlighted the need to develop the best-performing machine learning prediction models that are less complex, involve fewer parameters, and have a greater ability to generalize hidden patterns among the dependent and independent variables. To address these shortcomings, the linear kernel and tree-based prediction models were formulated for PCM-incorporated buildings located in eight cities with a subtropical highland climate, considering variations in their hyperparameters. The evaluation and validation of the formulated models were performed using several statistical metrics. The results demonstrated that the ensemble-boosted tree-based prediction model (EBT_20_) exhibited superior efficacy in estimating the energy consumption of PCM-integrated buildings, with an average R^2^ > 0.93; NMBE < 4%; and CV-RMSE < 8%. The developed model showed lower complexity and better generalization capability with optimized values of the ensemble-boosted tree hyperparameters: the minimum leaf size = 32; the number of learners = 99; and the learning rate = 0.1. A comprehensive evaluation conducted to assess the interpretability of the prediction model found that building orientation and PCM melting temperature were the most influential parameters for achieving energy savings of up to 33% in the selected climate zone.

Salihi et al. [265] predicted the cooling/heating energy consumption for PCM-integrated residential building envelopes using machine learning. In this study, five machine learning models were used: Multiple Linear Regression (MLR), Support Vector Regression (SVR), an Artificial Neural Network (ANN), a Generalized Additive Model (GAM), and Decision Tree (DT). These models considered variations in PCM thermophysical properties, location, and thickness. The dataset was generated through a parametric analysis using Energy Plus^®^ and JEplus simulation tools. The prediction computations were conducted using a Python-based computer program. Model performance was then assessed using three metrics: R^2^, the MAE, and the RMSE. The results indicate that the ANN model outperformed the others, achieving the lowest RMSE and MAE and the highest R-squared value, exceeding 0.99. Moreover, this study’s findings emphasized the potential of the ANN model in predicting energy consumption and offered valuable insights for stakeholders aiming to optimize heating and cooling energy consumption in PCM-incorporated residential buildings.

Mikhailnova et al. [266] developed a novel hybrid optimization and machine learning technique for energy storage in smart buildings using PCMs. The authors used Energy Plus^®^ software and a novel hybrid multilevel particle swarm optimization and convolutional neural network (H-MPSO-CNN) model. The performance of PCM in the walls and ceilings of buildings in the climates of Namangan, Uzbekistan, and Najran, Saudi Arabia, was investigated in this study. The study assessed the impact of variables such as melting temperature and the optimal location of PCM on heating and cooling load consumption. The results demonstrated that PCMs with melting points of 23 °C and 25 °C had the greatest impact in the Namangan climate, whereas the PCM with a temperature of 25 °C had the greatest impact in Najran. The study also evaluated the most suitable locations for PCM on walls and roofs. It was determined that such a system is better suited to Najran’s hot and dry climate. Heating and cooling loads in Namangan could be reduced by approximately 12.4 and 16%, respectively, by installing PCM systems in the roof and walls of the building. Similarly, a single-layer PCM system in Najran could reduce heating and cooling energy consumption by around 10% and 12%, respectively.

### 12.4. Gaps Between Simulations and Experiments

The key gaps between simulations and experiments in PCM-based passive design arise from the following factors:

- Material Properties: Simulations usually rely on generalized PCM properties, which may not account for practical real-world fluctuations such as aging, impurities, and fabrication inconsistencies.
- Boundary Conditions: Experimental setups may have complex boundary conditions that are difficult to replicate accurately in simulations.
- Thermal Hysteresis: Many simulations tools struggle to model the thermal hysteresis of PCMs, which affects their phase change behavior.
- Dynamic Interactions: Real-world interactions between PCMs and other building materials, such as thermal bridging or moisture transfer, are usually simplified too much in simulations.
- Occupant Behavior: Simulations rarely account for variability in human behavior including door and window opening or thermostat adjustments, which considerably impact energy consumption.
- Climatic Data: Simulations should use historical or average climatic information, which might not reflect microclimatic variations or future weather patterns.

It should be highlighted that simulation methodologies should improve to meet practical building design requirements. In this sense, here are some practical suggestions for simulation improvement:

- Enhanced Material Databases: Develop comprehensive databases with detailed PCM properties, including variations caused by aging and environmental factors.
- Advanced Modeling Techniques: Incorporate models to simulate thermal hysteresis and dynamic interactions more accurately.
- Integration of Real-Time Data: Use real-time monitoring data from experimental setups to validate and refine simulation models.
- Combined Simulations: Combine thermal, moisture, and structural simulations to capture the holistic behavior of PCM-integrated systems.
- User-Centric Design: Include stochastic models to simulate occupant behavior and its impact on the building thermal performance.
- Future-Proofing: Integrate predictive climate models to evaluate the long-term performance of PCM-based designs under changing weather conditions.

These improvements can help bridge the gap between simulations and experimental results, making simulations more reliable for practical building design.

## 13. Results and Discussion

The results from the reviewed studies demonstrate the potential of phase change materials (PCMs) to enhance thermal comfort and improve energy efficiency in residential buildings. The key findings and associated challenges are discussed below.

### 13.1. Thermal Performance and Energy Savings

The reviewed studies highlight that integrating PCMs into building elements such as walls, ceilings, and floors effectively reduces indoor temperature fluctuations and heating/cooling loads. Sun et al. [37] reported that PCM-integrated walls reduced the internal surface temperature amplitude by 21.4% in summer and 23.9% in winter, thereby improving overall thermal comfort. Kishore et al. [209] demonstrated that optimized PCM configurations reduced annual heat gains by up to 47.2% and heat losses by 8.3%, depending on climate conditions.

Energy savings have also been confirmed by several studies. Yu et al. [208] showed that PCM use in solar passive walls reduced heating demands by up to 6.4 GJ in spring and decreased steam usage by 4.7% annually, significantly improving energy efficiency. Similarly, Khan et al. [207] found that placing PCMs closer to the heat source minimized indoor temperature rise, confirming the importance of strategic positioning to maximize energy performance.

### 13.2. Challenges and Limitations for PCM Usage

Despite the promising results, the large-scale adoption of PCMs in residential buildings faces several challenges:

- Low Thermal Conductivity: Organic PCMs, such as paraffin, have low thermal conductivity, limiting the rate of heat transfer and reducing overall system efficiency. Strategies such as incorporating metallic foams and nanoparticles (e.g., CuO, Al_2_O_3_) have shown potential to enhance conductivity.
- Phase Separation: Inorganic PCMs, including salt hydrates, are prone to phase separation, leading to reduced thermal stability over time. Encapsulation techniques, such as microencapsulation and composite materials, have been explored to address this issue.
- Compatibility with Construction Materials: PCM leakage during phase transition can compromise the structural integrity of construction materials. Advanced encapsulation and composite materials have been proposed as solutions.
- Thermal Hysteresis: PCMs can exhibit different melting and freezing points, leading to inefficiency in heat storage and release.
- Durability: Prolonged use with repeated phase transitions may lead to degradation, reducing their effectiveness over time.
- Compatibility with Existing Materials: Incorporating PCMs into building structures (e.g., walls, ceilings) demands careful design and compatibility with existing materials.
- Limited Usability: Certain applications might limit their usability in specific climates or building types due to temperature range constraints.
- Standardization: The lack of universal standards or guidelines for PCMs’ selection and implementation in real-world scenarios complicates their adoption.
- Cost: The production costs of advanced encapsulated PCMs and composite solutions remain high, limiting market penetration.
- Life Cycle Costs: Maintenance and replacement costs are significant due to PCM aging or performance degradation over time. Cost effectiveness can vary, depending on factors like local energy tariffs and climate conditions.
- Return on Investment: Energy savings provided by the PCMs may take prolonged periods to offset the initial investment costs, particularly in the regions with moderate climates where passive heating/cooling benefits are limited.
- Material Sourcing: Natural PCMs (e.g., paraffin wax or salt hydrates) can have environmental impacts during extraction and processing like resource depletion.
- Manufacturing Energy Use: The production of synthetic PCMs, especially for advanced applications, often involves energy-intensive processes contributing to greenhouse gas emissions.
- Waste Management: The disposal of aged or damaged PCMs raises concerns, particularly with synthetic types, which may not biodegrade or could release harmful substances.
- Net Benefits: On the positive side, PCMs significantly reduce operational energy consumption in buildings and decrease reliance on fossil fuels and overall carbon emissions during their usage phase. When implemented sustainably, they can have a net positive environmental impact.

Enhancing the thermal performance of PCMs requires addressing these limitations. Studies have explored the use of nanocomposites and metallic foams to improve conductivity and phase stability. Maleki et al. [32] reported a 72.3% increase in thermal conductivity with CuO-based nanocomposites, while Aidi et al. [33] demonstrated that adding alumina and CuO increased heat transfer efficiency and delayed peak temperature fluctuations by up to 1.5 h.

Another challenge highlighted by [267] is the limited information regarding the thermal properties and phase change behavior of the PCMs in building elements. This uncertainty in PCM model parameters can affect the reliability of simulation results. For example, in building applications such as PCM-enhanced pipe insulation and multi-layered wall elements, the lack of accurate data can hinder effective design and optimization, ultimately impacting thermal efficiency and energy demand reduction.

A further disadvantage pointed out by Hu et al. [268] concerns nonlinear thermal properties and temperature hysteresis, complicating computational modeling. The phenomenon of hysteresis can lead to discrepancies in energy performance predictions, as it varies with measurement methods and the intrinsic properties of the material. Additionally, the phase change in the PCM occurs over a temperature range, which further complicates simulations. The authors emphasize the need for precise measurement techniques and a better understanding of hysteresis to increase the efficiency of PCMs in building systems, particularly under variable climatic conditions.

Cabanová et al. [269] also highlight challenges in using PCMs in buildings, including hysteresis, phase segregation, subcooling, and cycling stability issues. These thermophysical factors can hinder the effective discharge of stored energy. For instance, if a PCM does not transit smoothly between the phases, it may not release the heat efficiently during the night, reducing its effectiveness as a passive thermal energy storage technological solution. Addressing these limitations is crucial for optimizing PCM integration into building components, particularly in transparent glazing systems.

Adesina et al. [270] pointed out that challenges in using PCMs in concrete include reduced mechanical properties, reinforcement corrosion, and the unavailability of suitable PCMs on the market, which are often expensive. Additionally, the lack of long-term data on the impact of PCMs on concrete durability hinders acceptance among stakeholders. These limitations raise significant barriers to the widespread application of PCMs in construction, despite their potential sustainability benefits.

Schmerse et al. [271] states that one significant challenge in using PCMs in buildings is the variability in occupant behavior, such as ventilation habits and occupancy patterns, which can affect thermal performance. Research highlights that insufficient data on these behaviors complicate accurate simulation results. While PCMs can enhance energy savings and comfort, their effectiveness depends on understanding and predicting how occupants interact with the building environment, making this a critical limitation in PCM applications.

One of the limitations of PCMs is their relatively low thermophysical properties. In terms of thermal conductivity, it can be said that segmented encapsulation enhances the thermal conductivity of a PCM, but the value remains insufficient. Therefore, additional materials must be incorporated to improve properties such as thermal conductivity, specific heat, and latent heat. Numerous attempts to improve thermal conductivity have been made by various researchers. For example, Fan and Khodadadi [272] explored structures like fins or honeycombs made from high-thermal-conductivity metals, including copper or aluminum. Similarly, Al-Abidi et al. [273] improved a triplex tube heat exchanger by integrating inner and outer fins, which significantly enhanced the system’s thermal conductivity—achieving an increase of 86%—and shortened the charging time of the organic paraffin-based PCM RT82. Another approach to boosting thermal conductivity is incorporating thermally conductive additives into PCMs [274]. Li et al. [111] added 5% graphite in a nanocomposite form, resulting in a12-fold increase in the thermal conductivity. However, this also led to a decrease in natural convection and latent heat capacity (this will be discussed further in the section on thermophysical properties’ enhancement). In other studies, bio-PCMs were used due to their self-nucleating ability, reduced vapor pressure during melting, elevated latent heat, chemical stability, safety, and affordability [275]. Moreover, Choi et al. [276] implemented bubble injection techniques within PCM to limit thermal penetration and enhance its latent heat performance. When PCM is injected into bubbles, the difference in densities between the PCM and the bubbles causes the bubbles to move upward, which eliminates temperature stratification within the PCM. This results in decreased temperature penetration and an increase in latent temperature. To evaluate these effects, the authors experimented with PCM alone and PCM with injected bubbles, finding that the latter increased the latent temperature by 11% and reduced the temperature penetration by 28%.

Supercooling refers to the delay in the solidification process of phase change materials (PCMs) even after they have reached their freezing point [277]. This phenomenon negatively impacts the thermal performance of PCMs and is particularly observed in inorganic types [278]. Various strategies have been explored to mitigate supercooling [279], such as modifying the size of the microcapsules, adding nucleating agents or metallic particles prior to encapsulation, using microencapsulation techniques, and refining the formulation and architecture of the capsule shells.

The addition of nanoadditives (e.g., nanoparticles) has been employed to eliminate supercooling in various PCMs systems [280]. The size and shape of the nanoparticles are known to enhance the nucleation process during the crystal growth of PCMs. Furthermore, the nucleating agents should be close to the lattice parameter of the base material. Crystal growth initiates at extremely small nucleation sites, and to trigger crystallization, impurity particles or small crystals acting as seeds can be introduced into the liquid or formed during PCM synthesis. An increased surface area and strong polar OH-OH bonds on the surface are also effective in reducing the degree of supercooling. However, no effective method for completely preventing the supercooling and phase segregation of inorganic PCMs has been found so far. Consequently, several researchers have attempted to address the limitations associated with supercooling in PCMs. For example, Sutjahja et al. [281] investigated the influence of additives like SrCl_2_·6H_2_O, BaCO_3_, and K_2_CO_3_ on the supercooling of CaCl_2_·6H_2_O PCM, with SrCL_2_·6H_2_O showing the best performance in preventing supercooling.

He et al. [282] reported that titania nanoparticles in a BaCl_2_ aqueous solution decreased the supercooling degree of a PCM by more than 84.9%. Similarly, Ma et al. [283] found that nano-titania reduced the supercooling of Al_2_(SO_4_)_3_·18H_2_O by almost 88.4%. Metal nanoparticles, metallic oxide nanoparticles, and metal nitride nanoparticles—such as copper, alumina, and aluminum nitride—have also been shown to stimulate crystallization and reduce supercooling during solidification [284].

Zhang et al. [285] dispersed hydrophobic silica nanoparticles as nucleating agents into n-octacosane emulsions, reporting that a composite with 0.3 wt.% of silica nanoparticles was the most effective in preventing supercooling. Li et al. [286] studied the behavior of graphene, silica, and titania nanofluids in reducing the supercooling degree of pure water. Graphene was found to be the most effective, and even at a low concentration of 0.02 wt.%, it could eliminate supercooling in pure water. Salt-based or hydrate-based nanomaterials are preferred due to their lower additive volume and greater efficiency. According to the available literature, the supercooling of hydrated salts can be reduced by adding nucleating agents such as alumina and silica [287]. Xu and Ke [288] verified that using Na_2_B_4_O_7_·10H_2_ O as a nucleating agent effectively reduced the supercooling degree of sodium acetate trihydrate (CH_3_ COONa·3H_2_O). Other studies have reported that incorporating between 2 wt.% and 3 wt.% of Na_2_B_4_O_7_·10H_2_ O in Na_2_SO_4_·10H_2_O reduced the supercooling degree to below 3 °C. However, the non-homogeneous distribution of nucleating agents within the PCM core can lead to inconsistent supercooling reduction.

To address the supercooling issue, Wang et al. [289] developed composite PCMs using three-dimensional graphene (3D-rGO) and Na_2_B_4_O_7_·10H_2_O synthesized by vacuum impregnation. Compared to traditional modification methods, the supercooling degree was further reduced with 3D-rGO. With a Na_2_B_4_O_7_·10H_2_O content of 1%, the supercooling degree of the composite PCM was reduced to 0.1 °C—a reduction rate of 99.2% compared to that of pure hydrated salt PCM. The improvement was attributed to the uniform dispersion of the nucleating agent within the composite PCMs containing 3D-rGO, which enhanced the nucleation rate and crystallization effect.

Supercooling can also be reduced through porous matrix optimization in PCMs. Researchers have argued that the most effective way to minimize supercooling and phase separation is to incorporate a porous structural confinement. Porous materials with varying pore structures serve as ideal support for PCMs, helping to control the relationship between phase separation and supercooling through a nanopore-confinement approach. Manufacturing a porous support tailored to the specific PCM is essential for this purpose [290].

Calcium carbonate with a porous structure, which is non-toxic, inexpensive, and environmentally friendly, has been identified as a promising supporting material for PCMs. Two- or three-dimensional porous materials with high surface areas have gained significant attention from the research community. Small molecules or ions accumulating in the pores of these materials introduce region-dependent surface reactivity. Porous structures can be fabricated through sol–gel methods with soft or hard templates, anodization, and the Kirkendall effect.

Zhang et al. [291] fabricated CaCO_3_ with pore sizes ranging from the macro- to the microlevel by calcining at different heating rates. A PCM composed of CaCO_3_ with 55 nm macropores and Na_2_HPO_4_·12H_2_O showed improved performance compared to Na_2_HPO_4_·12H_2_O alone or commercially available CaCO_3_. The composite PCM exhibited a single exothermal peak at 16 °C, whereas pure Na_2_HPO_4_·12H_2_O showed three peaks at 11 °C, 6.5 °C, and −18 °C. Non-porous CaCO_3_ also exhibited three peaks at 9 °C, 3 °C, and −19 °C, confirming that macroporous CaCO_3_ effectively reduced supercooling compared to normal CaCO_3_ or pure PCMs.

Liu et al. [292] prepared porous alumina ceramics with porosities ranging from 62% to 83% using a freeze-casting method. These porous ceramics were used as supporting frameworks for sugar alcohol PCMs, resulting in effective, leakage-proof, and shape-stabilized composite PCMs. The impact of different additives and alumina content on the porosity of the ceramics was also studied. The thermal conductivity of ET-APC-56 reached approximately 4.8 W/(m·K), with a substantial increase of 690% compared that of to pure erythritol. The supercooling degree of ET-APC-28 was reduced from 59.1 °C to 8.9 °C.

Gypsum plasterboards are widely utilized as interior finishing materials in buildings, primarily due to their acceptable fire-resistant properties [293]. Banu et al. [294] evaluated the fire behavior of gypsum wallboards integrated with roughly 24 wt.% of paraffin-based PCM. The results revealed that these modified boards did not pass the flammability test and performed worse than traditional gypsum boards. This shortcoming was attributed to the evaporation and leakage of the PCM during high-temperature exposure, which led to the emission of flammable vapors under standard fire conditions [295]. Several studies have addressed this issue. Sittisart and Farid [296] conducted a study on form-stable PCMs with various organic PCMs containing fire retardants to minimize fire hazards. The results showed that the intumescent fire retardant was the best option to reduce the flammability risk with minimal alteration to the thermophysical characteristics of the PCM.

Phase segregation is a phenomenon that occurs when PCM composites are not in the same phase. This can lead to instability in PCMs [297]. It often occurs in multi-component PCMs due to density differences, where the components separate because of gravity, causing the melting process to occur at different times. Phase segregation can also result from the incongruent melting of salt hydrate PCMs [298]. Ryu et al. [299] studied the effect of thickening agents to prevent phase segregation and found that incorporating between 3 and 5 wt.% of Na_2_SO_4_10H_2_O as thickener eliminated phase segregation.

After addressing some of the defects in PCM technology and proposing solutions, it should be noted that too many modifications and additions to PCMs can significantly increase their overall cost. In a study examining low-cost and environmentally friendly alternatives with satisfactory heat capacity and good stability, Boussaba et al. [300] used composite bio-PCMs that showed stability after 2000 cycles and passed toxic and flammability tests.

### 13.3. Optimal Placement and Configuration

The positioning of PCM layers within the building envelope significantly affects thermal performance. Kant et al. [206] observed that placing PCMs between layers of fiberglass insulation reduced heat transmission by up to 37.13%. Ouhsaine et al. [211] showed that the inner surface temperature of PCM-based walls was inversely related to wind speed and positively correlated with emissivity, indicating that higher emissivity surfaces maximize the cooling effects of PCMs.

Moreover, integrating PCMs into solar thermal systems and building facades, in combination with shading devices, has shown promising potential for optimizing energy savings. For example, Velasco et al. [212] demonstrated that PCMs placed in the facades of buildings could reduce energy consumption by 35%, particularly when coupled with proper ventilation strategies and window coatings.

### 13.4. Comparative Analysis of PCM-Based Solutions

To provide a comprehensive overview of the different PCM-based solutions for thermal management in residential buildings, Table 2 summarizes the main types of PCMs and their thermal properties, advantages, disadvantages, and typical applications. This comparative analysis highlights the strengths and limitations of each solution, offering valuable insights for optimizing the use of PCMs in various building configurations.

Furthermore, to illustrate the practical implementation of PCM-based solutions, Table 3 presents a detailed comparison of studies that explore the use of PCMs in different building elements, such as roofs, ceilings, walls, windows, and floors. This table provides some specific examples from the literature, highlighting the achieved thermal performance improvements and energy savings.

## 14. Recommendations for Further Studies

Based on the research conducted by the authors presented herein, several challenges and areas for future development are identified. Future research should focus on the following:

- Developing high-conductivity composite PCMs through nanoparticle integration.
- Enhancing encapsulation methods to prevent phase separation and leakage.
- Designing cost-effective manufacturing processes to facilitate large-scale adoption.
- Investigating the long-term stability of PCMs under cyclic thermal loads.

In addressing these challenges, several specific recommendations are proposed to enhance the effectiveness of PCMs in improving the thermal performance of residential buildings:

- Numerical simulation has been proven to be a powerful tool for investigating the thermal properties of nano-enhanced PCMs. These methods allow for the accurate description of thermal properties at the molecular scale, providing insight into energy transport mechanisms and helping to understand the enhanced thermal conductivity resulting from the dispersion of nanomaterials into the base PCM.
- Among the most detailed approaches is molecular dynamics simulation, which computes the motion of individual molecules. This technique has been widely applied to understand the microscopic behavior of materials. For example, Babaei et al. [301] employed molecular dynamics simulations to explore the thermal conductivity enhancement of paraffin when combined with carbon-based nanofillers.
- Further studies should focus on improving the accuracy of these simulations, using real-time measurements of heat flux and temperature distribution in PCM-integrated building systems. Moreover, long-term experimental investigations are essential to better understand the aging behavior and reliability of PCMs over extended periods. To make PCMs more accessible for large-scale implementation, cost-effective and scalable manufacturing processes must also be prioritized.
- Currently, the most suitable climates for the use of PCMs are those presenting considerable temperature changes within a day, but with little temperature variations across the year. Further research to develop PCMs with less supercooling could help to expand their potential areas of application.
- While phase change materials are most investigated for applications in outward-facing building parts like walls and roofs, recent research has also discussed their possible application in floors, storage heaters, and blinds. Further studies on the matter should contemplate full lifetime cycle assessments to infer the effectiveness of PCMs in green buildings.
- The risk of leakage is one of the main challenges linked with PCM, which can be overcome via encapsulation. The incorporation of nanomaterials in these encapsulating polymers has also been demonstrated to increase thermal conductivity. The usage of nanoparticles playing the role of nucleating agents can also aid in reducing supercooling, enabling their application in regions with lower daily temperature gradients.
- The elevated flammability of PCMs when applied in residential buildings is also one of the most relevant concerns and challenges. Further research on the incorporation of fire retardants in the PCMs composites should, at least, partially overcome the flammability limitation. Fire retardants like melamine-based compounds should be further examined in practical situations.
- More and more efficient composites should be studied at the laboratory scale and in real practical applications in buildings. For example, the inclusion of PCMs and nanocomposites has even been found to improve the compressive strength of cement. It should be stated that further research would be able to mitigate the challenges associated with the exploration of PCM composites.
- It is suggested to perform more experimental works on the incorporation of fatty acids and corresponding composites such as eutectic PCM into cement by the vacuum method to decrease the indoor temperature during the melting and solidification periods. Also, the inclusion of PCMs in roofs can reduce their temperature and heat gain.
- The usage of PCMs in passive cooling systems in warm-temperature climates has been more studied than that in other climates, and considerable cooling energy savings have been shown. Nonetheless, it is recommended to perform more experimental and numerical works on heating-dominant climates to optimize the melting temperatures of PCMs to attain higher total annual savings.
- To further optimize the design and implementation of PCM curtain windows, approaches to depress/suppress natural convection in air gaps should be studied. Also, a cascaded PCM layer with distinct melting points in the vertical direction may be used. One shortcoming of PCM curtains is that the transparency of windows will be lost when adding an opaque layer, and this will require extra lighting.

## 15. Conclusions

To contextualize our findings, it is useful to compare this review with other recent studies on PCM applications in buildings. For instance, Reddy et al. [302] provide a comprehensive overview of PCM integration across various building components, including roofs, walls, floors, and windows, with a focus on material composition and energy efficiency in both residential and non-residential contexts. In contrast, our study specifically targets PCM applications in residential buildings, offering a detailed analysis of thermal performance metrics, such as energy savings of 15–30% in walls and floors, and practical challenges, including leakage, cost, and climate-specific optimization. By narrowing the scope to residential settings, we highlight opportunities for tailored PCM solutions, such as shape-stabilized PCMs for floors and nanoencapsulated PCMs for windows, which are critical for enhancing thermal comfort in homes. This focused approach complements broader reviews like Reddy et al. [302], providing stakeholders with actionable insights for residential energy efficiency.

- PCMs can be categorized into organic, inorganic, and eutectic materials. The organic class can also be divided into paraffin and non-paraffin materials, and the inorganic category can be separated into salt hydrates, metallic PCMs, and eutectic PCM s. Encapsulated PCMs must be encapsulated by macroencapsulation, microencapsulation, or nanoencapsulation.
- The selection of the most adequate base PCMs and nanomaterials is essential to develop effective nano-enhanced PCM s. For building applications, PCMs used should have a melting temperature in the desired range of the practical operation of the buildings. The first approximation proposed by the authors Peippo et al. [303] could be useful for determining the ideal melting temperature of PCMs to be used in building envelopes. PCMs to be considered for building applications should have a high latent heat of fusion, elevated specific heat capacity, high thermal conductivity, and minimum sub-cooling and should be chemically stable, non-toxic, non-flammable, and non-corrosive.
- The use of PCMs in building components can strongly benefit the energy efficiency of a building, primarily by temperature modulation in both hot and cold climates, leading to lowered energy consumption, as well as a shift in electrical load.
- There are many possible applications of PCMs in residential buildings like those in bricks, concrete, walls, wallboards, gypsum, and plaster and many other applications. To choose the adequate PCM, thermophysical and chemical properties and economic parameters should be considered, among other factors.
- Researchers have explored the integration of PCMs in various building elements, including vertical walls, partitions, floors, ceilings, and attic floors, as well as components of green and cool roofs. Among these applications, PCM-enhanced wallboards have gained significant attention due to their ease of incorporation on interior walls and ceiling surfaces, cost effectiveness, and proven ability to moderate indoor temperatures and reduce cooling energy demand. Additionally, recent studies have investigated the use of PCMs to improve the performance and durability of cool roofing systems, highlighting their potential for broader implementation in passive thermal regulation strategies.
- Further numerical investigations are essential to explore the benefits of coupling PCM-based passive systems with natural night ventilation, aiming to enhance their overall thermal performance. This review of the available literature showed that there is no detailed work on such synergistic systems using more sophisticated numerical methods.
- To overcome the actual challenges closely linked with the incorporation of PCMs in building materials like thermal conductivity, cooling time, flammability, and phase segregation, the addition of certain materials to PCMs can form composite materials with enhanced characteristics; for example, the inclusion of copper oxide can prevent low thermal conductivity.
- The changing of temperature during the day should be regulated by PCMs; in the cooling period, PCMs should be solidified during the night when the ambient temperature is lower to afford cooling during the day when the ambient temperature is higher. Accordingly, PCMs should melt during periods of elevated ambient temperature to afford the required heating capability in the heating period. Consequently, the melting point of PCMs must be carefully selected, relating to the average temperature during the year, to become solidified and melted every day. A too-high or too-low melting point results in phase stability that eliminates energy storage. Thus, the optimization of the location is vital to attain the most suitable interaction with the surrounding conditions.

## Figures and Tables

**Table 1:** Summary of key findings from reviewed studies on PCM applications in residential buildings.

PCM Type	Integration Method	Building Element	Thermal Performance	Challenges	References
Organic (e.g., Paraffin)	Microencapsulation	Walls	Reduced indoor temperature fluctuations by 2–9 °C; energy savings up to 30%	Low thermal conductivity; high encapsulation cost	[12,13,14,15,16]
Organic (e.g., Paraffin)	Shape-stabilization	Floors	Latent heat capacity of 98–148 J/g; improved thermal inertia by 1–2 h	Leakage issues; reduced mechanical strength	[17,18,19,20]
Organic (e.g., Dodecyl Alcohol)	Shape-stabilization	Wall Coverings	Enhanced thermal regulation; energy consumption reduced by 15–25%	Limited long-term stability; cost of novel materials	[21]
Inorganic (e.g., Salt Hydrates)	Macroencapulation	Bricks/ Walls	Enhanced thermal storage density; temperature control improved by 1.5–3 °C	Supercooling; corrosion with metals	[22,23,24,25,26]
Inorganic (e.g., Salt Hydrates)	Direct Incorporation	Ceilings	Energy consumption reduced by 15–20%; stable after 5000 cycles	Phase separation; compatibility issues	[27,28,29]
Eutectic Mixtures	Nanoencapsulation	Windows	Increased thermal conductivity by 73–136%; delayed peak temperature by 1.5 h	High production cost; limited scalability	[30,31,32,33,34]
Organic (e.g., Paraffin)	Microencapsulation	Roofs	Reduced cooling load by 10–20%; improved thermal comfort in hot climates	Climate-specific optimization needed; high initial cost	[35,36,37]

**Table 2:** Comparison of PCM solutions.

Type of PCM	Incorporation Method	Thermal Properties	Advantages	Disadvantages	Applications
Organic (paraffin and non-paraffin)	Direct, encapsulation (micro-, macro-)	High latent heat (150–250 kJ/kg)	Has good thermal stability, is non-corrosive	Low thermal conductivity	Walls, ceilings, floors
Inorganic (hydrated salts)	Direct, immersion, encapsulation	High thermal conductivity, possible supercooling	Is low cost, has good conductivity	Corrosion issues, phase segregation	Walls, concrete
Eutectic mixture (organic–organic, inorganic–organic)	Encapsulation, shape stabilization	Customized melting point	Combines properties of different PCMs	Chemical compatibility challenges	Facades, roofing
Shape-stabilized PCM	Shape stabilization with polymers or nanotubes	Good thermal stability, leakage prevention	Prevents leakage, maintains structural integrity	High production cost	Internal coatings, thermal clothing
Encapsulated PCM (micro-, macro-, nano-)	Chemical or physical encapsulation	Increased heat exchange area	Prevents leakage, has higher thermal conductivity	High cost, impact on mechanical properties	Concrete, mortar

**Table 3:** A comparison of studies on the use of PCMs in different building elements for thermal performance and energy savings.

Principal Use	Main Points	Details
PCM Integration	Use of PCMs in various parts of buildings	Application in walls, ceilings, floors, windows, and even movable elements (such as curtains) can regulate indoor temperature.
Roofs	Thermal performance of roofs with PCM	Integrating PCMs into roofs improves heat storage efficiency, depending on the PCM properties.
	Bhamare et al. [187] study (India)	The MKR index is introduced to select the ideal PCM for different climatic zones in India.
	Sedaghat et al. [193] study (hot climates)	PCM integration in walls and roofs results in up to 53% energy savings, depending on the climate region.
Ceilings	Thermal performance of ceilings with PCMs	Multiple studies show that PCMs can reduce cooling load and improve thermal comfort in buildings.
	Basher et al. [187] study (Iraq)	PCM thickness and orientation in ceilings affect indoor temperature reduction and energy consumption.
	Velasco-Carrasco et al. [196] study (S23 panels)	PCM ceiling tiles that absorb heat and stabilize temperature provide an efficient alternative for thermal comfort.
Walls	PCM use in radiant walls	PCM in radiant walls can reduce energy consumption and improve cooling system efficiency.
	Plytaria et al. [205] study (Greece)	PCM in walls increases thermal storage capacity and reduces the need for the frequent operation of absorption chillers.
Windows and Shutters	Integration of PCMs in windows and shutters	Using PCM windows can significantly reduce indoor heat gain and improve energy efficiency.
	Sudha et al. [217] study (thermal performance of windows with PCM)	PCM windows reduce indoor temperature and air conditioning demand, delaying peak temperature by up to 2 h.
	Zhang et al. [223] study (smart windows with PCM)	Thermally responsive PCM windows that change color to control sunlight and store heat enhance thermal efficiency.
Floors	PCM use in floors	PCM floors help regulate thermal comfort and improve the performance of heating and cooling systems.
	Fu et al. [228] study (Adjustable composite PCM)	Composite PCM with an adjustable melting point for radiant floors improves thermal comfort and reduces supercooling.
	Tang et al. [229] study (PCM floor with expanded graphite)	Radiant PCM floors with expanded graphite improve thermal comfort, achieving a high satisfaction rate.
	Sun et al. [235] study (double-layer radiant floor system with PCM)	Double-layer PCM radiant floors provide extended thermal comfort in both winter and summer conditions.

## Data Availability

No new data were created or analyzed in this study. Data sharing is not applicable to this article.
